# Isolating Brain Mechanisms of Expectancy Effects on Pain: Cue-Based Stimulus Expectancies versus Placebo-Based Treatment Expectancies

**DOI:** 10.1523/JNEUROSCI.0050-25.2025

**Published:** 2025-07-28

**Authors:** Elizabeth A. Necka, Titilola Akintola, Qingbao Yu, Carolyn M. Amir, Olga Oretsky, Lauren Y. Atlas

**Affiliations:** ^1^National Center for Complementary and Integrative Health, National Institutes of Health, Bethesda, Maryland 20892; ^2^National Institute of Mental Health, National Institutes of Health, Bethesda, Maryland 20892; ^3^National Institute on Drug Abuse, National Institutes of Health, Baltimore, Maryland 20852

**Keywords:** analgesia, expectancy, fMRI, learning, pain, placebo

## Abstract

Clinical trials, laboratory experiments, and neuroimaging studies provide converging evidence that pain is highly sensitive to expectations, whether based on the psychosocial context surrounding treatment (e.g., placebo analgesia) or transient cues that provide information about painful events (e.g., pain-predictive cues). We asked whether placebo analgesia and pain-predictive cues modulate pain through the same mechanisms or dissociable brain pathways. Forty healthy volunteers of both sexes rated pain in response to noxious heat during functional magnetic resonance imaging. We crossed pain-predictive cues, which induce expectations for high or low pain on a trial-by-trial basis, with administration of an inert placebo cream or a control cream. Behavioral analyses revealed a significant interaction, such that predictive cues had weaker effects on pain during placebo blocks than control blocks. This interaction was accompanied by interactions in the insula, pons, and other brain regions. We also observed distinct neural substrates when we compared pure cue effects with pure placebo effects, and only predictive cues modulated responses in nociceptive regions and the neurologic pain signature (Wager et al., 2013). The only regions that were influenced similarly by both types of expectations were the rostral anterior cingulate and dorsomedial prefrontal cortex. These results indicate that cue-based expectations about stimulus intensity and placebo-based expectations about treatment outcomes are distinct and that pain researchers should differentiate between sources of expectations. Furthermore, cue-based expectations were associated with more consistent effects than treatment-based expectations, suggesting that clinicians should be particularly mindful of how they present information about impending pain.

## Significance Statement

This study measured the impact of pain-predictive cues and placebo effects on expectations, pain, and brain responses to noxious heat. Expectations were influenced by both cues and placebos, and we observed interactions between the two types of expectations on both pain and pain-related brain responses. We also observed dissociations in the two types of expectancy effects on brain responses and found that placebo effects were more variable than cue effects across individuals. This suggests that different types of expectations are associated with different underlying mechanisms. To maximize patient outcomes, clinicians should carefully frame both information about painful procedures and information about analgesic treatments.

## Introduction

Expectations shape perception across domains, including pain. Placebo analgesia—pain reduction in response to an inert treatment—is attributable to expectations associated with the psychosocial treatment context. Meta-analyses of clinical trials that compared placebo-treated groups with natural-history control groups indicate that placebo effects are the largest in pain and subjective outcomes (Hróbjartsson and Gøtzsche, 2001, 2004). Reported pain reductions in placebo analgesia are accompanied by changes in endogenous opioids (Levine et al., 1978; Zubieta, 2005; Wager et al., 2007; Eippert et al., 2009a) and shifts in brain activation in response to noxious stimulation (Atlas and Wager, 2014). However, recent large-scale studies indicate that placebo analgesia is accompanied by shifts in circuits related to value and decision-making (Botvinik-Nezer et al., 2024), rather than nociceptive circuits as measured by a brain-based nociceptive pain classifier, the neurologic pain signature (NPS; Wager et al., 2013; Zunhammer et al., 2018).

Pain is influenced not only by expectations about treatments but also by expectations about painful events, e.g., noxious stimulus intensity. For example, a dental patient's expectations can reflect experiences with local anesthetics such as lidocaine (treatment expectations) as well as the dentist's forewarning that the lidocaine injection will only hurt a little bit (stimulus expectations). Stimulus expectancies have been primarily studied through pain-predictive cues, in which classical conditioning and/or verbal instructions pair neutral cues with painful outcomes to induce cue-based expectations. Pain-predictive cues elicit reliable modulation of subjective pain (Keltner, 2006; Brown et al., 2008) and shape brain responses in nearly all regions that respond to noxious stimuli (Atlas et al., 2010). Thus, cues about upcoming noxious stimulation can induce flexible expectations that dynamically shape pain on a trial-by-trial basis. In contrast to placebo analgesia, pain-predictive cues can modulate NPS expression (Woo et al., 2017).

Although many discuss these areas interchangeably, we have hypothesized that placebos and pain-predictive cues operate through separate mechanisms (Atlas et al., 2010; Atlas and Wager, 2012, 2014) for several reasons. First, placebo-based expectations about treatments are likely to induce response expectancies—expectations about how one will feel—but not stimulus expectancies, expectations about what will occur in the environment (Bolles, 1972; Kirsch, 1985, 1997). Cue-based expectations induce both stimulus and response expectancies, as they generate predictions about something that will occur as well as the pain it will evoke. Second, placebo analgesia and cue-based expectations have distinct effects on pain-related brain responses based on neuroimaging meta-analysis (Atlas and Wager, 2014) and NPS expression (Woo et al., 2017; Zunhammer et al., 2018). We have hypothesized that they rely on dissociable neuromodulatory systems, such that only placebos would engage endogenous µ-opioid signaling, which is associated with long-lasting descending pain modulation, while predictive cues should elicit phasic dopamine prediction errors, which can vary rapidly and flexibly update expectations. Although formal comparisons are lacking, dopamine agonists do not modulate placebo analgesia (Wrobel et al., 2014; Kunkel et al., 2024), consistent with hypothesized dissociations. Finally, a behavioral study that crossed predictive cues with open and hidden administration of the µ-opioid agonist remifentanil (Atlas et al., 2013) indicated treatment-based expectancy effects (i.e., open vs hidden drug administration), and cue-based expectancy effects were additive, consistent with separate mechanisms. Thus, both stimulus expectancies and treatment expectancies can shape pain during analgesic treatment. Understanding the mechanisms by which treatment versus stimulus-based expectations modulate pain is critical for identifying how to leverage expectations to ameliorate pain.

**Figure 1.**
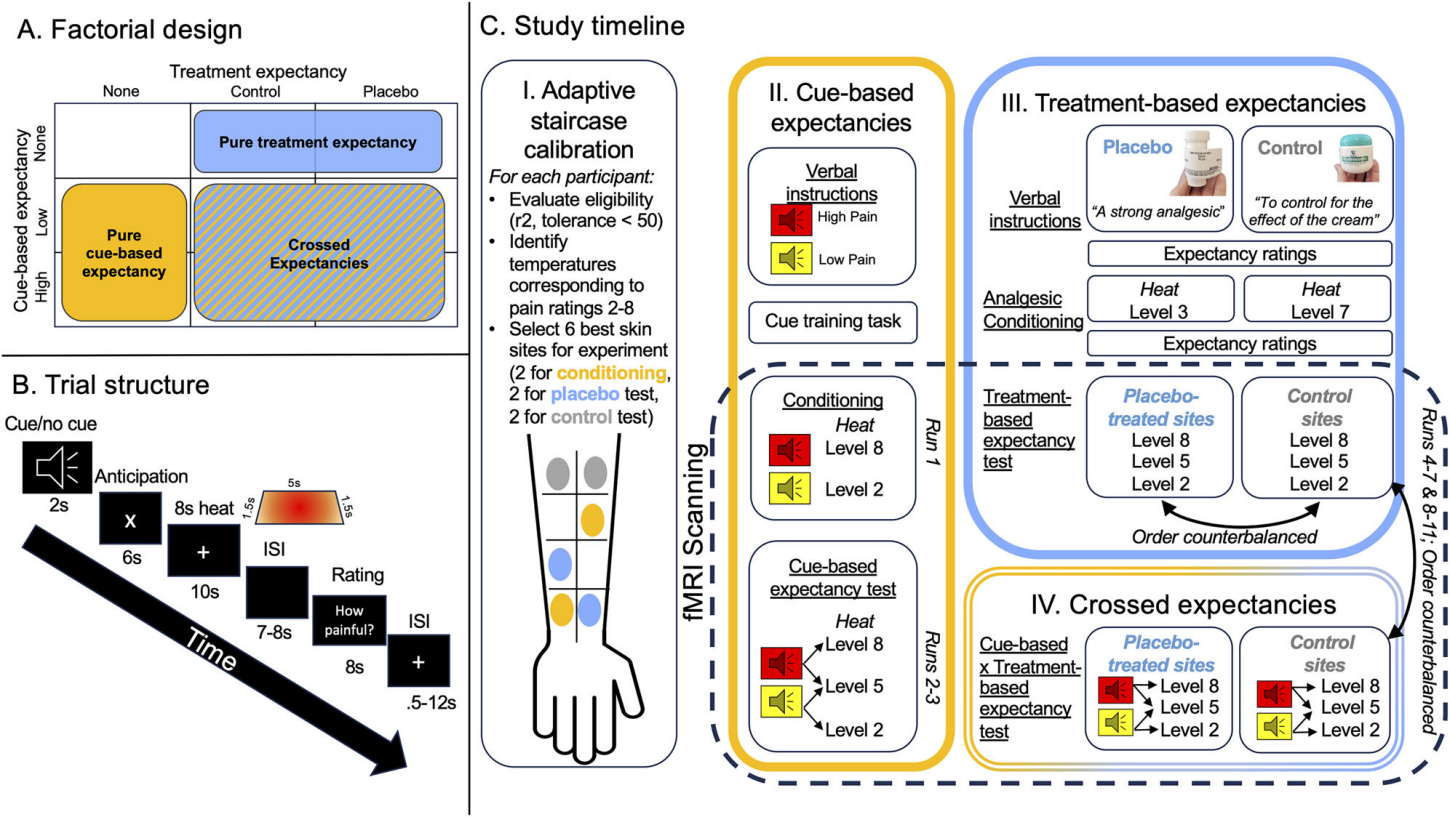
Experimental design. ***A***, Quasi-factorial design. Forty participants underwent a quasi-factorial design in which treatment expectancy (placebo vs control) was crossed with cue-based stimulus expectancies (high vs low pain-predictive cues). We also tested treatment expectancy in the absence of stimulus expectancies and stimulus expectancies in the absence of treatment expectancies. ***B***, Trial structure. Each run of fMRI scanning consisted of eight 39 s trials. On cued blocks, trials began with a 2 s auditory cue (counterbalanced across participants), followed by 6 s of anticipation, and then 8 s of heat from a thermode was administered during a 10 s fixation window. Following an interstimulus interval (ISI), participants rated their pain on that trial using a VAS. A variable ISI preceded the next trial. Uncued trials were initiated with a 2 s fixation rather than an auditory cue. ***C***, Study timeline*.* ***I***, All participants underwent an adaptive staircase calibration (Amir et al., 2022) prior to fMRI scanning to evaluate eligibility and identify temperatures and the six most reliable skin sites for use during the fMRI experiment. Two sites were used for cue-based expectancy runs and analgesic conditioning (orange), two sites were used for placebo test runs (blue), and two sites were used for control test runs (gray). Skin sites varied across participants. ***II***, Following the calibration, eligible participants (*n* = 40) were instructed about cue contingencies and underwent a cue training task to reinforce verbal instructions. They were then situated in the MRI scanner and underwent a conditioning run, in which low cues were followed by heat stimulation calibrated to elicit low pain (pain threshold or Level 2 on the VAS), and high cues were followed by stimulation calibrated to elicit high pain (pain tolerance or Level 8 on the VAS). Following conditioning, participants underwent two runs of scanning in which each cue was followed by a temperature calibrated to elicit medium pain (Level 5) rather than a temperature calibrated to elicit high or low pain, on 50% of the trials, which provided a test of cue-based stimulus expectancies. ***III***, After the third run, the scanner bed was moved out of the bore, and participants underwent a treatment expectancy manipulation, in which they were given verbal instructions that an inert topical cream (the placebo) was a strong analgesic and that a second cream (control) would be applied to control for effects of the cream. They then underwent analgesic conditioning, in which participants were told that two skin sites would be stimulated with a Level 7 stimulus to test if the treatment was working, and a reduced temperature (Level 3) was applied to one placebo-treated skin site. Following the treatment manipulation, participants were returned to the isocenter and underwent eight runs, half on the placebo-treated skin site, and half on the control site (order counterbalanced across participants). Within each condition, half the trials were uncued, which provides pure tests of treatment expectancy effects on pain. ***IV***, The other half of trials in each treatment condition included pain-predictive cues, which allowed us to test formal interactions between placebo-based treatment expectancies and cue-based stimulus expectancies.

We tested the preregistered hypothesis that treatment expectancies and stimulus expectancies modulate pain through independent neural mechanisms. We used a quasi-factorial design to cross stimulus and treatment expectancies during functional magnetic resonance imaging (fMRI) scanning (Fig. 1) and evaluate additivity or interactions. We hypothesized that cue effects on pain would be mediated by neural systems involved in nociceptive pain (i.e., NPS) and value-based learning (e.g., striatum), while treatment expectancies would modulate neural systems involved in top–down control [e.g., dorsolateral and ventromedial prefrontal cortex (VMPFC), periaqueductal gray (PAG), rostral anterior cingulate]. We expected both types of expectancies to modulate pain-related regions and that the degree of involvement might vary based on both stimulus and treatment expectations. Finally, we hypothesized that prefrontal regions and pain-processing regions would show opposing interactions between stimulus and treatment expectancies during noxious stimulation.[Table T1]

**Table 1. T1:** Behavioral results on fully crossed trials^a^

Predictors	Pain rating		*p*
	Estimates	CI	
(Intercept)	3.47	3.14–3.80	<0.001
Stimulus expectancy	1.21	0.95–1.47	<0.001
Treatment expectancy	−0.27	−0.58–0.05	0.097
Stimulus expectancy × treatment expectancy	−0.4	−0.77 to −0.03	0.036
Random effects			
*σ* ^2^	1.33		
*τ*_00__Subject_	1.01		
*τ*_11__Subject.Cue_centered_	0.34		
*τ*_11__Subject.Analgesic_centered_	0.64		
*ρ*_01_	0.55		
	−0.13		
ICC	0.48		
*N*_Subject_	40		
Observations	602		
Marginal *R*^2^/conditional *R*^2^	0.131/0.552		

a This table presents results of a multilevel model examining effects on medium trials that were fully crossed with treatment expectancy (control > placebo) and stimulus expectancy (high cue > low cue). Predictors were mean-centered prior to analysis. Analyses were implemented using lmer from the R package lme4 (Bates et al., 2015).

## Materials and Methods

All procedures were approved by the Institutional Review Board of the National Institutes of Health (NIH; NCT02446262) and followed the ethical guidelines for human subjects research in the Declaration of Helsinki.

### Participants

Participants were recruited from a community sample at the NIH Clinical Center as part of a larger study on psychological factors influencing pain perception (clinicaltrials.gov NCT02446262, “Neural and psychological mechanisms of pain perception”). All participants completed an initial screening visit that included a clinical exam and quantitative sensory testing to establish eligibility for multiple experiments. Participants were invited to enroll in the current study if they (1) were right-handed and exhibited no contraindications for MRI, (2) had no history of chronic pain or regular or recent use (i.e., within 24 h) of medication affecting pain, (3) had no history of major neurological or psychiatric conditions or conditions affecting pain sensitivity or somatosensation, (4) exhibited no dermatological conditions on their left forearm (the site of somatosensory testing), and (5) had not previously completed a study of placebo analgesia at the NIH. Sixty-two participants enrolled in the current study and provided informed consent at the NIH FMRI Facility (FMRIF). We verified eligibility prior to fMRI scanning (see below, Procedures). Twelve participants were ineligible based on a pain calibration procedure (see below). Data collection was incomplete for 10 additional participants due to technical issues (*n* = 6), discomfort (*n* = 3), or inability to comply with the instructions (*n* = 1) leaving a final sample of 40 participants (age, *M* = 32.01; SD = 8.5; range, [20–49]; 55% male; 45% White; 20% Black/African American; 12.5% Asian; 12.5% Hispanic/Latino; 7.5% more than one race; 7.5% unknown) who completed the task and were included in analyses.

### Materials

#### Thermal stimulation

Participants received thermal stimulation to their left volar (inner) forearm delivered via a 16 × 16 mm Peltier thermode (TSA-II, Medoc), with temperatures ranging from 32 to 50°C. Each stimulus lasted 8 s, with 5 s at peak temperature and a 1.5 s ramp-up and ramp-down period. Temperatures were selected based on an adaptive staircase calibration procedure (Amir et al., 2022).

#### Pain ratings

Participants rated pain using a visual analog scale (VAS) ranging from 0 (no sensation at all) to 10 (most pain imaginable). Participants were additionally instructed that pain threshold corresponded to a rating of 2, moderate pain corresponded to a rating of 5, and 8 denoted pain tolerance (i.e., the most pain they could tolerate). During scanning, participants provided ratings and expectancy ratings using an fMRI-compatible track ball (Current Designs). On each trial, the 0–10 VAS scale appeared for 3 s. Participants then had 5 s to use the trackball to move an arrow along the scale and indicate their rating. We included mousetracking during this period, which was used to recover missed trials (see below, Behavioral analyses).

### Procedures

#### Informed consent

Upon arrival to the FMRIF, participants provided informed consent, during which they were informed that the purpose of the study was to test how different treatments can modify pain and the brain's response to pain and that they would receive a strong topical analgesic during the study. Consistent with recommendations for the ethical use of deception in placebo studies (Miller et al., 2005), participants were informed that at some point during the experiment they might receive inaccurate or misleading information and that if they were assigned to that condition, researchers would debrief them at the end of the study. (In actuality, all participants were informed that an inert substance was an analgesic and were debriefed following study completion.) All participants provided a urine sample (to test for recreational drug use and pregnancy) and completed an fMRI safety screening form. Participants also completed a series of questionnaires, which may be analyzed separately in future research.

#### Adaptive staircase calibration (Fig. 1CI)

Temperatures delivered to participants during the main task were individually calibrated to each participant based on their responses in an established heat pain calibration task (Amir et al., 2022). Across three rounds of testing, participants received heat stimulation to each of the eight skin sites on the arm and provided verbal pain ratings on a 0–10 scale, as described above. We used iterative linear regression to determine which temperatures were associated with pain threshold (2), moderate pain (5), and pain tolerance (8), and each site was tested once with each level (24 total trials); see Amir et al. (2022) for complete details. When low-, medium-, and high-intensity heat failed to elicit reliable differences in pain ratings for at least six skin sites, additional trials of heat stimulation were administered to ensure that participants could tolerate the temperature predicted to elicit Level 8 pain and could detect the temperature predicted to elicit Level 2 pain. Following calibration, a linear fit for the effect of temperature on pain (i.e., *R*^2^) was computed, and average residuals for each site were calculated. Participants were dismissed following the calibration when (1) the maximum pain tolerance was >50°C (*n* = 2); (2) *R*^2^ was <0.4 (*n* = 3); or (3) fewer than six skin sites exhibited differential responses to low-, medium-, and high-intensity heat (*n* = 7). For all other participants, the temperatures predicted to elicit low pain (Level 2; *M* = 42.84°C; SD = 2.32), medium pain (Level 5; *M* = 45.63°C; SD = 1.79), and high pain (Level 8; *M* = 48.14°C; SD = 1.33), and the six most reliable skin sites (based on the lowest average residual) were selected for use in the main task. As described below, discrete skin sites were used for the cue expectancy runs (the best site and fourth most reliable site), the first treatment condition based on counterbalanced order (second and fifth best sites), and the second treatment condition (third and sixth best sites). We also identified temperatures corresponding to Levels 3 (*M* = 43.75°C; SD = 2.14) and 7 (*M* *=* 47.33°C; SD = 1.50) for use during the treatment expectancy manipulation phase, which was applied to the same sites used for the pretreatment cue expectancy runs.

#### Cue training task (Fig. 1CII)

Participants underwent a cue training task before being situated in the MRI scanner. They were instructed that during the main task, they would experience different levels of heat and that they would “sometimes hear cue sounds before the heat which indicate how painful the heat may be.” Participants then heard two sounds (a cymbal and a door chime), one of which they were told would be associated with high pain and the other of which they were told would be associated with low pain. Sounds lasted 2 s and were counterbalanced across participants. Participants then completed a cue training task in which they heard a sound, classified it as a low or high pain cue by pressing the L or H keys on a keyboard, and received feedback. Participants completed 24 trials in a random order and had to correctly classify at least 21 trials to proceed to the main task (all participants succeeded).

#### fMRI task design

Following calibration and cue training, participants were situated in the MRI scanner and completed 11 functional runs that lasted 5 min and 22 s each. The thermode was placed on a different skin site prior to each run. As addressed in more detail below, the first three runs measured cue-based expectancies, while Runs 4–11 tested treatment-based expectancies and interactions between cue-based and treatment-based expectancies. Physiological data (eyetracking, skin conductance, heart rate, respiration) were recorded throughout the study and will be analyzed separately in future research.

#### Cue-based expectancies (Fig. 1CII)

Participants were told that Runs 1–3 were to establish “a baseline measure of how you respond to the heat pain stimuli.” They were also reminded about cue–outcome contingencies (e.g. “The sound you are about to hear is associated with LOW pain”) prior to functional scans. During Run 1, high and low pain cues preceded high (Level 8) and low (Level 2) intensity thermal stimulation, respectively, to further reinforce instructed cue-based stimulus expectancies through conditioning. During Runs 2–3, medium (Level 5) intensity thermal stimulation was surreptitiously administered on 50% of trials preceded by a high pain cue (high cue + medium heat or “HM”) and 50% of trials preceded by a low pain cue (low cue + medium heat or “LM”). Because these trials were matched on intensity (temperature) and varied only on the cue that preceded the temperature, these trials critically permit investigation of cue-based expectancy modulation of pain, consistent with prior work (Atlas et al., 2010). Before each run, participants heard each cue and were asked to rate how much pain they expected using the same scale that they used during the experiment.

#### Treatment expectancy manipulation (Fig. 1CIII)

After Run 3, the bed of the scanner was removed from the scanner bore, and the anterior head coil was removed. Participants were instructed to remain still and refrain from shaking or nodding their head; participants remained supine throughout the manipulation, except for in exceptional cases where a subject sat up for miscellaneous reasons (e.g., to reposition head pillows due to discomfort; *N* = 3). Participants were informed that a nurse practitioner would apply a “strong topical analgesic” that is “similar to lidocaine” to three skin sites and a research assistant would apply skin lotion to three other skin sites to control for “the effect of having a cream on their skin.” In actuality, both creams were an identical inert cream manufactured by the NIH pharmacy. The nurse practitioner then showed participants the placebo cream (which was labeled with a pharmaceutical sticker with the participants’ name and “NIH Compound 821 L; Cream; 15gm”), applied the cream, covered skin sites with the cream with medical dressing, and waited 2 min before removing the dressing. Participants were told that the dressing was important to help stimulate the effects of the medication. During the waiting period, participants verbally rated how effective they expected the analgesic to be in reducing their pain on a 0 (not at all effective) to 10 (completely effective) scale.

After the researcher applied the control cream, participants were told that it was necessary to “test that the medication was working” before the nurse practitioner left. Participants were told that for this test, they would receive heat at the same Level 7 intensity to one analgesic-treated site and one control site and that they should rate the pain that they felt. In actuality, participants received Level 7 thermal stimulation only on the site with the control cream and received Level 3 thermal stimulation on the site with the placebo cream. The treatment conditioning was applied to the same skin sites that were used during the first three runs in the same order, i.e., the first treatment (placebo or control, based on counterbalanced order) applied to the most reliable skin site. Participants completed eight trials of analgesic conditioning (four to each site, interleaved) and then verbally rated how effective they expected the analgesic using a 0–10 verbal scale. Following expected efficacy ratings, participants were returned to the isocenter in the scanner bore.

#### Crossing treatment-based expectancies with cue-based expectancies (Fig. 1CIV)

Following the placebo manipulation, participants completed four runs in which thermal stimulation was administered to two skin sites treated with the placebo cream and four runs in which it was administered to two skin sites treated with the control cream (counterbalanced); these sites were distinct from those that were used during the treatment expectancy manipulation so as to avoid carryover effects between conditioning and test, consistent with prior work (Wager et al., 2004). Half of the runs omitted cues to test pure treatment-based expectancy effects, while half of the runs included predictive cues to test interactions between cue-based and treatment-based expectancies (Fig. 1*CIV*). Noxious stimuli were equivalent across all runs following the treatment expectancy manipulation: medium (Level 5) intensity thermal stimulation was administered on 50% of trials, and low (Level 2) and high (Level 8) intensity thermal stimulation were each administered on 25% of trials. As with the baseline portion, on runs with cues, medium intensity thermal stimulation was preceded equally by high and low pain cues. The temperatures delivered on each trial of each run were pseudorandomized, with the only restriction being that the first trial of each run could never be a medium intensity stimulus. At the start of each run, participants were always instructed on-screen and verbally on which site the thermode was placed and whether they would hear cues prior to stimulation (e.g., “In the next few blocks, we will administer heat stimuli to a site with the analgesic cream. You will have cues before each trial.”). On runs with cues, participants were also asked about expected pain prior to the run onset.

#### Trial structure

Each run consisted of eight 39 s trials (Fig. 1*B*). On cued blocks, participants saw a 2 s cue followed by a 6 s anticipatory period during which a fixation cross was presented centrally on the screen; on uncued blocks, the anticipatory period lasted 8 s. Following the anticipatory interval, thermal stimulation was delivered for 8 s (5 s at peak) at the target temperature determined during calibration. Following thermal stimulation, there was a jittered 7–8 s delay, after which the words “How painful?” and an image of the VAS used during calibration appeared on the screen for 3 s. Next, an arrow appeared, and participants used the track ball to move the arrow across the scale and rate their pain. Participants had 5 s to make a pain rating, after which there was a 0.5–12 s interstimulus interval before trial conclusion.

#### FMRI data acquisition

MRI data were acquired with a 32-channel head coil on two 3 T Siemens Skyra scanners at the NIH FMRI and NMR Facilities. Functional images were acquired with a T2*-weighted, multiecho pulse sequence with a repetition time (TR) of 2,500 ms and echo times (TE) of 11, 22, and 33 ms (ascending interleaved acquisition; field of view 70 × 64 × 40; flip angle, 90°). We collected axial slices using an in-plane resolution of 3.286 × 3.286 mm and 3 mm slice thickness. Slices were tilted 30° relative to horizontal (i.e., the anterior commissure–posterior commissure) to recover dropout in orbitofrontal regions (Deichmann et al., 2003). In some participants, this placement led to a lack of coverage in superior parietal and occipital lobes, meaning our findings are agnostic as to the contributions of these regions. We thus created a mask to identify voxels in which at least 80% of participants had valid data, which was used in group-level analyses as discussed below. Each run lasted 337.50 s (135 TRs). Whole-brain T1-weighted high–resolution anatomical images were acquired (192 1 mm slices, FOV = 256 × 256 mm; TE, 2.07 ms; TR, 1,900 ms; flip angle, 9°). We conducted a second localizer scan in cases when head position changed during the treatment expectancy manipulation (*N* = 3) and accounted for this during preprocessing, as discussed below.

### Experimental design and statistical analyses

#### Study design, sample size determination, and analytical strategy

Study design, approach for sample size determination, and analytic strategy were reviewed by the NINDS Scientific Review Committee prior to approval by the NIH IRB, and the clinical protocol (15-AT-0132) was registered prior to data collection on clinicaltrials.gov (identifier NCT02446262).

Our study included three within-subject factors: heat intensity (high, medium, low), cue (high, low, no cue), and treatment (no treatment, control, placebo). The design was quasi-factorial, as we did not cross no treatment and no cue conditions (Fig. 1*A*), and only the medium level of heat intensity was crossed with all levels of cue (Fig. 1*C*), consistent with prior work (Atlas et al., 2010). Following the placebo manipulation, we counterbalanced two factors across participants: placebo versus control and cue versus no cue. Runs 2–3 (cue + no treatment) served as pure tests of cue-based expectations on pain, four runs served as pure tests of treatment-based expectations on pain (two no cue + placebo, two no cue + control), and four runs crossed cue-based expectations and treatment-based expectations [(cue + placebo) vs (cue + control)] to test for additive or interactive effects. As all factors were crossed on medium trials, our strongest tests of cue- and treatment-based expectations evaluate pain and brain responses on medium heat trials. We also report main effects of heat intensity and evaluate exploratory interactions between treatment and heat intensity. We considered order effects separately due to our complex quasi-factorial design.

The protocol specifies that each fMRI substudy will include 20 participants per group, unless power analyses based on pilot studies require >20 participants per group. A pilot study of 12 participants (six per group based on the counterbalanced order of placebo and control) indicated that a sample size of 17 would have sufficient power to detect a cue effect on behavior, based on the magnitude of the cue effect combined across placebo and control blocks (Cohen's *d*z = 0.96; the sample size computed in G*power; Faul et al., 2009). As this number is less than our clinical protocol’s a priori estimate, data collection was considered complete when we had 20 participants per group. Forty participants completed the experiment and were included in behavioral analyses. Three participants were excluded from FMRI analyses due to technical issues (*n* = 2) or excess movement during scanning (*n* = 1).

Because our protocol described planned imaging analyses and hypotheses at a high level, we preregistered detailed analysis plans and specific regions of interest after collecting data but before conducting neuroimaging analyses (https://aspredicted.org/IIX_EQG, registered on 2018/09/12 after final data point was collected on 2018/08/04). We describe any deviations from preregistration in Extended Data Figure 3-4.

### Behavioral analyses

Behavioral analyses of participants' pain ratings were conducted in *R* using linear mixed models implemented with the function “lmer” from the R package lme4 (Bates et al., 2015). To facilitate interpretation of model intercepts and coefficients, all within-subject factors were mean-centered relative to each participant, and all between-subject factors were centered relative to the grand mean; this has the effect of disaggregating between- and within-person effects and to facilitate statistical inference on fixed effects at both levels (Wang and Maxwell, 2015). We first evaluated maximal models that included random intercepts and slopes for all fixed factors. All maximal models converged except for the model that tested interactions between treatment and stimulus expectancy on medium cued trials; in this case, we used the R package “buildmer” (Cesco Voeten, n.d.) to find the maximal feasible model and conduct step-wise elimination using likelihood ratio tests to find the most parsimonious model, which removed random slopes for the interaction term. We computed effect sizes (Cohen’s *d*) of linear mixed model results by dividing fixed effect coefficients by the square root of the sum of random factors for each model, as per Westfall et al. (2014).

Due to unforeseen technical issues with the scanner-compatible mouse, two participants were unable to select a response on the scale and were instead instructed to move the mouse to their rating and then stop scrolling. Because we enabled mousetracking for all participants (sampling rate 10 ms), we were able to impute scores for these participants by identifying where the mouse was on the scale when the mouse stopped moving. For all other participants, trials on which no response was made were excluded from analyses, as were trials during blocks when the thermode was not in proper contact with the skin. On average, there were 1.55 missing trials per participant (SD, 2.77; range, [0,12]) or 1.8% of total trials per participant.

### fMRI analyses

#### Preprocessing and single-trial analyses

Preprocessing was conducted in AFNI (Analysis of Functional NeuroImages; Cox, 1996) using the afni_proc.py command. Anatomical images were warped to MNI space using nonlinear warping implemented through AFNI's @SSwarper command and then skull-stripped. We used anatomical images to create subject-specific masks and identify the number of participants with valid data for each voxel, which was used during cluster-based small volumes correction, as well as to create a group mean anatomical image, which was used as the underlay in all figures.

Functional data were slice time and motion corrected and aligned to the echoplanar image of the second echo from the first three (i.e., baseline) runs that had the fewest outliers (determined using the 3dToutcount function) using the “lpc + ZZ” cost function (i.e., a local Pearson correlation with an additional optimization step, as recommended in Taylor et al., 2018) and either the “giant_move” option, which increases the search space for alignment to account for potential changes in head placement or orientation during the analgesic conditioning procedures, or the “ginormous move” option, in the case of three participants with a second localizer scan and one participant for whom anatomical scans were collected on a previous day. Functional data were then aligned to warped anatomical data, and the three echoes were then optimally combined using the AFNI program “@compute_OC_weights” which computes weights across echoes based on a system of equations relating TE and T2* (Posse et al., 1999; Kundu et al., 2012).

Following optimal combination, we used the custom MATLAB code available through Canlab Core Tools (https://github.com/canlab/CanlabCore) to generate trial-level estimates for use in single -trial analyses. Specifically, the hemodynamic response function (HRF) to heat was fit using a flexible basis set consisting of three curves shifted in time and customized for thermal pain responses based on previous studies (Lindquist et al., 2009; Atlas et al., 2010), allowing the HRF to vary across trials and voxels, in a model including six movement parameters per run from AFNI (as well as movement squared, movement lag 1 TR, and movement lag 1 TR squared) and nuisance covariates for a linear effect of time across the scan and for spikes on TRs where the average voxel value deviated from the moving average of the average voxel value of 20 FWHM TRs by >10 median absolute deviations. After fitting the HRF, the height, width, time-to-peak, and area under the curve (AUC) were computed for each voxel, and we computed variance inflation factors (VIFs) for each trial to determine whether trials coincided with spikes, motion, or other sources of noise, which could inflate trial estimates. Analyses presented here were conducted on smoothed AUC estimates, consistent with prior work (Atlas et al., 2010, 2012, 2014a). Images were smoothed using a 6 mm FWHM (twice the voxel size; Friston et al., 1996).

#### Participant-level design matrices

For each participant, voxelwise beta estimates and contrasts were generated using the custom MATLAB program “fitGLM.m,” available through Canlab Core Tools (https://github.com/canlab/CanlabCore), which implements a general linear model across single-trial estimates [see Atlas et al.( 2010) for complete details]. Consistent with prior work (Atlas et al., 2010, 2012, 2014a), we modeled autoregression with a factor of 1, and trials with VIFs that exceeded 2.5 were omitted from all analyses (*M* = 2.54 trials excluded, i.e., 2.98% of trials; SD = 2.48; range, [0,13]). For each participant, a first-level GLM design matrix was constructed across trials that modeled regressors for each condition [i.e., each combination of heat level (low, medium, high), cue (uncued, low, or high), and treatment (baseline, control, placebo)], leading to 18 regressors (e.g., high/high/baseline, high/high/control, high/high/placebo, etc.); we also included two regressors to model changes across trials during the placebo block and the control block. Second-level contrasts were computed across trials. Consistent with our preregistration, we contrasted responses to high versus low heat prior to the treatment expectancy manipulation to isolate the nociceptive network (pretreatment high > pretreatment low). We also constructed contrasts to measure the impact of cue-based (i.e., stimulus based) expectancies on medium trials (HM > LM) during each phase of the experiment [i.e., (baseline HM > baseline LM), (placebo HM > placebo LM), and (control HM > placebo LM)] and to measure treatment-based expectancy effects across all temperatures (control > placebo), within medium trials (medium during control blocks > medium during placebo blocks), and with and without cues [(uncued medium during control > uncued medium during placebo); (cued medium during control > cued medium during placebo)]. To evaluate main effects, we searched for regions that showed differential activation as a function of expected pain, i.e., differences between high pain expectancy and low pain expectancy [(HM + control) > (LM + placebo)]. We coded interactions in two ways: (1) we compared responses on crossed trials (i.e., cued trials under placebo vs treatment) to examine the combined impact of stimulus and treatment expectations on pain [(HM during placebo + LM during control) > (LM during placebo + HM during control)]; (2) we compared responses on uncrossed trials [i.e., uncued placebo and control blocks vs cue effects prior to treatment; (baseline HM > baseline LM) > (uncued medium during control > uncued medium during placebo)] to examine whether the impact of high or low pain expectations on experienced pain and brain responses depends on whether the expectations were derived from cues or treatment.

#### Robust regression

Voxelwise robust regression (Wager et al., 2005) was used to conduct second-level analyses across subject-level contrast maps. Robust regression has the strength of downweighting outliers and modeling main effects and interactions with subject-level covariates. To account for individual differences in the magnitude of placebo analgesia, we measured the association between each contrast and individual differences in placebo effects (operationalized as average pain rating across all temperatures on uncued placebo blocks minus average pain response across all temperatures on uncued control blocks). All robust regressions controlled for the counterbalanced order of cues and creams.

#### Voxelwise multilevel mediation analysis

We used multilevel mediation analysis to identify brain mediators of expectancy-based pain modulation, consistent with prior work (Atlas et al., 2010, 2022). Mediation was implemented with the M3 toolbox (Wager et al., 2009) to conduct voxelwise linear mixed models across single-trial estimates, and bootstrapping was used to estimate the significance of the mediation effect (Shrout and Bolger, 2002; Kenny et al., 2003). We used mediation analyses to examine pure expectancy effects on pain; thus our first mediation analysis examined stimulus-based expectancy effects on pain prior to the treatment expectancy manipulation, while our second mediation analysis examined treatment-based expectancy effects on pain on uncued trials. For consistency, we only modeled medium heat trials in both mediations, and trials with VIFs that exceeded 2.5 were omitted from analyses. Thus for both mediations, *X* was expectancy [(high > low) or (control > placebo)], *Y* was pain in response to medium heat, and M was the brain response to medium heat. This results in three interpretable brain maps: Path *a* is the effect of expectancy on brain response; Path *b* is the association between brain activation and pain controlling for expectancy; and Path a*b identifies voxels that mediate expectancy effects on pain, i.e., that explain significant variance in the behavioral expectancy effect. We evaluated within-subject mediation regardless of individual differences and also tested for moderated mediation by including subject-level individual differences in placebo analgesia based on average differences in pain between uncued control blocks and uncued placebo blocks. Moderated mediation also controlled for the order of cues and creams.

#### Small volumes correction

We preregistered three types of a priori regions of interest for the current analyses: (1) the NPS (Wager et al., 2013), (2) regions involved in expectancy-based pain modulation [i.e., those identified through prior meta-analysis of placebo and expectancy based on Atlas and Wager (2014)], and (3) the pain-processing network, i.e., brain regions that differentiated between high versus low stimulation in the current sample during baseline, prior to expectancy manipulation. To generate a combined mask of regions involved in expectancy and pain, we combined results of our prior meta-analysis (Atlas and Wager, 2014) with a mask of regions that differentiated between high and low heat stimulation based on mega-analysis (Atlas et al., 2010). Consistent with our preregistration, we used AFNI’s 3dttest++ program and the option -Clustsim (Cox et al., 2017) to implement nonparametric cluster-based FDR correction for multiple comparisons for tests within the a priori pain and expectancy network, with *p* < 0.001 as the cluster-defining threshold (Woo et al., 2014; Eklund et al., 2016). We performed FDR correction within pain-processing network regions and tested effects on the NPS using a standard threshold of *p* < 0.05.

In addition to reporting results within these preregistered networks, we report whole-brain FDR correction in the main manuscript and provide uncorrected whole-brain results in Extended Data for use in future voxelwise meta-analyses. Finally, we include tests of modulation of the stimulus intensity independent pain signature pattern (SIIPS; Woo et al., 2017), as this classifier has been shown to be modulated by expectancy and placebo in prior work (Woo et al., 2017; Botvinik-Nezer et al., 2024).

#### Preregistered hypotheses

We preregistered the following hypotheses: (1) relative to low pain expectancy, high pain expectancy will elicit increases in the pain-processing network [anterior and posterior insula, thalamus, ACC, secondary somatosensory cortex (SII), supplementary motor area (SMA), and cerebellum], limbic regions (amygdala, hypothalamus), and regions involved in prediction error/aversive learning (ventral striatum, caudate, putamen) and decreases in regions that show deactivation in response to high, relative to low, pain [precuneus, parahippocampal gyrus, VMPFC, orbitofrontal cortex (OFC)]; (2) relative to uncued trials, stimulus expectancies (high and low cues) will be associated with increased activation in regions involved with prediction error; (3) treatment expectancy would be associated with reductions in the pain-processing network during placebo relative to control and placebo-induced increases in regions involved in top–down control [dorsolateral PFC (DLPFC), ventrolateral PFC (VLPFC), OFC, PAG, rostrodorsal/pregenual/rostral ACC (rACC)]; (4) behavioral placebo responses (mean difference in pain reports on placebo relative to control and baseline trials) would be correlated with brain responses during pure treatment expectancy blocks and crossed blocks; (5) pain-processing networks, the NPS, and regions involved in value-based learning would mediate stimulus expectancy effects on pain but not treatment expectancy effects; and (6) we would observe positive interactions between stimulus and treatment expectancy in regions involved in top–down control (DLPFC/VLPFC/OFC) and negative interactions in pain-processing network regions including the insula. We also preregistered hypotheses regarding the effect of time (trial within condition), cued relative to uncued trials, and background PAG–rACC connectivity, which are addressed and reported in Additional preregistered analyses in the Results section.

## Results

### Expectations vary based on stimulus and treatment expectancy

**Figure 2.**
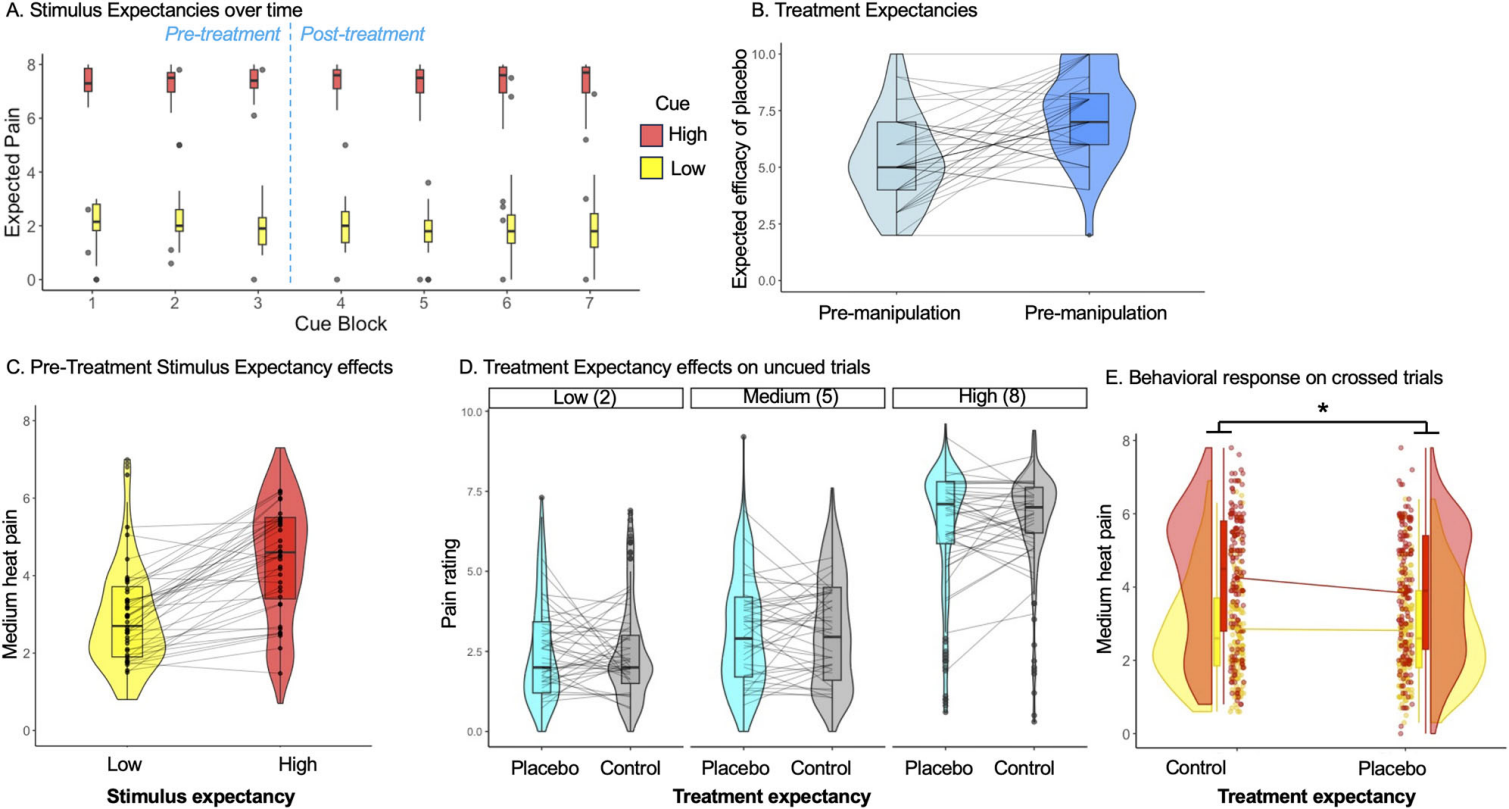
Expectancy effects on expectations and pain reports. ***A***, Participants expected higher pain with high pain cues (red) relative to low pain cues (yellow) across the entire study. ***B***, Participants expected moderate pain relief following verbal instructions about the analgesic cream (light blue). Expected pain relief increased following the analgesic conditioning phase, in which a reduced temperature was applied to placebo-treated sites (medium blue). ***C***, Prior to the treatment phase, nearly all participants reported lower pain when the same medium heat stimulus was preceded by low pain cues (yellow) relative to high pain cues (red). ***D***, On uncued trials, there was a main effect of heat intensity on pain, but no consistent effect of treatment expectancy across participants (cyan, placebo; gray, control). ***E***, Crossing cues and treatment expectancy on medium trials provides our direct test of interactions (see Table 1). We found a significant interaction, such that cue effects on pain were reduced under placebo administration (right) relative to control administration (left). There were no relationships between treatment expectancies and stimulus expectancies (see Extended Data Fig. 2-1).

We first tested whether conditioning and verbal instructions were sufficient to induce expectancies about pain by measuring subjective expectations. We evaluated whether pain-predictive cues influenced expectations by comparing participants’ expected pain as a function of cue, prior to the treatment manipulation. Participants expected more pain in response to high cues relative to low cues (Fig. 2*A*; *M*_High_ = 7.04; SD_High_ = 1.60; *M*_Low_ = 2.18; SD_Low_ = 1.23; main effect of cue, *F*_(1,6)_ = 206.91; *p* < 0.001). The magnitude of the effect was similar when we measured cue effects on medium trials across all blocks, regardless of treatment expectancy (*M*_High_ = 7.09; SD_High_ = 1.60; *M*_Low_ = 2.12; SD_Low_ = 1.33; main effect of cue, *F*_(1,4)_ = 32.0; *p* < 0.001; Fig. 2*A*).

10.1523/JNEUROSCI.0050-25.2025.f2-1 Figure 2-1 *Relationships between stimulus and treatment expectancy effects.* In exploratory analyses, we evaluated whether there were correlations between cue effects and treatment effects. A) *Cue-based expectancy effects.* Cue-based stimulus expectancy effects were consistent across individuals, as evident by correlations between pre-treatment (“Baseline”) cue effects (HM > LM) and cue effects on pain during control treatment (left) and placebo treatment (middle), as well as correlations in cue effects between placebo and control blocks (right). B) *Treatment based expectancy effects.* Treatment-based expectancy effects on pain (Control > Placebo) were correlated on cued and uncued medium heat trials (top left), but there were no associations across temperatures (top right), or were there any correlations with between treatment-based expectancy effects and cue-based stimulus expectancy effects, regardless of cues or temperatures (middle and bottom rows). Download Figure 2-1, TIF file.

Next, we evaluated whether our treatment manipulation influenced subjective expectations. Participants were first provided with verbal instructions about the analgesic cream. Following verbal instructions, participants expected moderate pain relief with placebo, relative to the control cream (*M*_premanipulation_ = 5.33; 95% CI = [4.70, 5.95]; *t*_(39)_ = 17.36; *p* < 0.001; Fig. 2*B*). Participants then underwent conditioning, in which temperatures were surreptitiously reduced on the placebo-treated sites. Following conditioning, participants expected significantly higher pain relief (*M*_postmanipulation_ = 7.24; CI = [6.61, 7.88]; *M*_postmanipulation_ − *M*_premanipulation_ = 1.91; CI = [1.04, 2.78]; *t*_(38)_ = 4.43; *p* < 0.001). Thus our combination of instructions and conditioning were sufficient to induce expectations based on both cue-based stimulus expectancies and placebo-based treatment expectancies. We next asked whether these expectancies in turn shaped pain and brain activation in response to noxious stimulation.

#### Pain is modulated by heat, stimulus expectancies, and interactions between treatment and stimulus expectancy

We used linear mixed models to examine the effect of heat level (high > medium > low), stimulus expectancy (high cue > low cue), and treatment expectancy (placebo > control) on subjective pain across all trials.

We first examined pain as a function of stimulus expectancy and heat level prior to the treatment manipulation. We observed a main effect of the heat level (*B* = 1.79; CI = [1.60, 1.99]; *p* < 0.001; Cohen's *d* = 1.05), such that stimulus temperature was positively associated with pain (high, *M* = 7.05; SD = 0.61; medium, *M* = 3.62; SD = 0.99; low, *M* = 2.00; SD = 0.74), a main effect of stimulus expectancy (*B* = 1.48; CI = [1.15, 1.82]; *p* < 0.001; Cohen's *d* = 0.86), and a significant stimulus expectancy ×  heat level interaction (*B* = 1.81; CI = [1.33, 2.29]; *p* < 0.001; Cohen's *d* = 1.05). When we tested effects on the critical medium heat trials that were crossed with high and low cues (i.e., testing cue-based expectancies while effectively holding heat constant), we observed a main effect of stimulus expectancy (*B* = 1.47; CI = [1.14, 1.81]; *p* < 0.001; Fig. 2*B*), such that participants reported higher pain when medium heat was preceded by high pain cues (*M* = 4.34; SD = 1.29) relative to low pain cues (M = 2.87; SD = 0.93), and the effect size of this difference was large (Cohen's *d* = 0.85). Results were similar when we examined relationships between stimulus intensity and pain across all trials. When we measured pain across all cued trials as a function of cue and heat intensity, we observed a main effect of heat (*B* = 1.74; CI = [1.57, 1.91]; *p* < 0.001; Cohen's *d* = 1.01), a main effect of cue (*B* = 1.3; CI = [1.04, 1.55]; *p* < 0.001; Cohen's *d* = 0.75), and a significant cue × heat interaction (*B* = 1.96; CI = [1.59, 2.33]; *p* < 0.001; Cohen's *d* = 1.14), driven by cue effects on medium heat trials (which were the only temperatures crossed with cues).

Next, we examined pain on trials following the treatment manipulation. We focused first on uncued trials to isolate pure effects of treatment expectancy, which complement our analysis of pure stimulus expectancy prior to the treatment manipulation. Comparisons between cued and uncued trials and relationships with counterbalancing order are reported in Supplementary Analyses. We observed a main effect of the heat level on pain on uncued trials (*B* = 2.04; CI = [1.87, 2.22]; *p* < 0.001; Cohen's *d* = 1.05; Fig. 2*D*), but no influence of treatment expectancy on pain, nor any heat × treatment expectancy interaction (all *p*'s > 0.2; all Cohen's *d* < 0.1). We note that participants did report moderate efficacy of treatment in post-task ratings (*M*_post-task_ = 5.59; 95% CI = [4.77, 6.42]; *t*_(36)_ = 13.8; *p* < 0.001; Cohen's *d* = 2.27), which reflected a small but significant reduction from pretask/postmanipulation expectations (*M*_post-task_ – *M*_postmanipulation_ = −1.63; CI = [−2.43, −0.82]; *t*_(35)_ = −4.08; *p* < 0.001; Cohen's *d* = 0.74). Although there were no main effects of treatment, there was substantial variability as is evident in individual slopes in Figure 2*D*, such that 21 participants reported placebo effects on uncued trials (less pain with placebo cream), while 19 reported nocebo effects on uncued trials (higher pain with placebo cream). We tested whether this variation was related to expected analgesic efficacy based on the manipulation (i.e., using postmanipulation expectation ratings) by including expected analgesia as a subject-level factor and testing for interactions. When we evaluated relationships with expected analgesia, we observed a marginal interaction between treatment and expected analgesia (*B* = −0.15; CI = [−0.31, 0.01]; *p* = 0.068; Cohen's *d* = 0.07), driven by increased pain on control runs in those who expected greater placebo efficacy (*B* = 0.19; *p* = 0.053; Cohen's *d* = 0.1). There was no relationship between expected efficacy and pain on placebo runs (all *p*'s > 0.4) nor between placebo analgesia and cue effects on pain prior to treatment (all *p*'s > 0.2). Due to the substantial individual differences in placebo analgesia, placebo analgesia was treated as a moderator in all neuroimaging analyses of treatment expectancy effects.

Finally, we examined responses on cued trials following the treatment manipulation to test for relationships between stimulus expectancy and treatment expectancy. We again observed a main effect of heat level (*B* = 1.62; CI = [1.40, 1.84]; *p* < 0.001; Cohen's *d* = 0.93), as well as a main effect of stimulus expectancy (*B* = 1.2; CI = [0.95, 1.46]; *p* = 0.001; Cohen's *d* = 0.69) and a heat level × stimulus expectancy interaction (*B* = 2.01; CI = [1.75, 2.27]; *p* < 0.001; Cohen's *d* = 1.15). We did not observe a main effect of treatment expectancy (*p* > 0.08; Cohen's *d* = −0.15). However, we observed significant interactions between treatment expectancy and heat (*B* = 0.35; CI = [0.08, 0.61]; *p* = 0.01; Cohen's *d* = 0.20), between treatment expectancy and stimulus expectancy (*B* = −0.39; CI = [−0.76, −0.02]; *p* = 0.04; Cohen's *d* = −0.22), and between treatment expectancy, stimulus expectancy, and heat level (*B* = 0.65; CI = [0.13, 1.18]; *p* = 0.015; Cohen's *d* = 0.37). To understand interactions, we evaluated expectancy effects within each heat level. Low and high heat trials were only crossed with treatment expectancy, while medium trials were evenly crossed with both stimulus expectancy and with treatment expectancy. Consistent with results on uncued trials, there were no main effects of treatment expectancy on pain in any condition (all *p*'s > 0.2). On medium trials (Table 1), we observed a significant main effect of stimulus expectancy (*B* = 1.21; CI = [0.95, 1.47]; *p* < 0.001; Cohen's *d* = 0.62) and a significant stimulus × treatment expectancy interaction (*B* = −0.4; CI = [−0.77, −0.003]; *p* = 0.036; Cohen's *d* = 0.20), driven by larger cue effects (HM–LM) on control blocks (*B* = 1.4; CI = [1.09, 1.71]; *p* < 0.001; Cohen's *d* = 0.76) than placebo blocks (*B* = 1.01; CI = [0.7, 1.33]; *p* < 0.001; Cohen's *d* = 0.57), as shown in Figure 2*E*. Thus placebo-based treatment expectancies reduced the impact of cue-based stimulus expectancies on subjective pain.

#### Effects of noxious stimulus intensity on pain and heat-evoked brain responses

**Figure 3.**
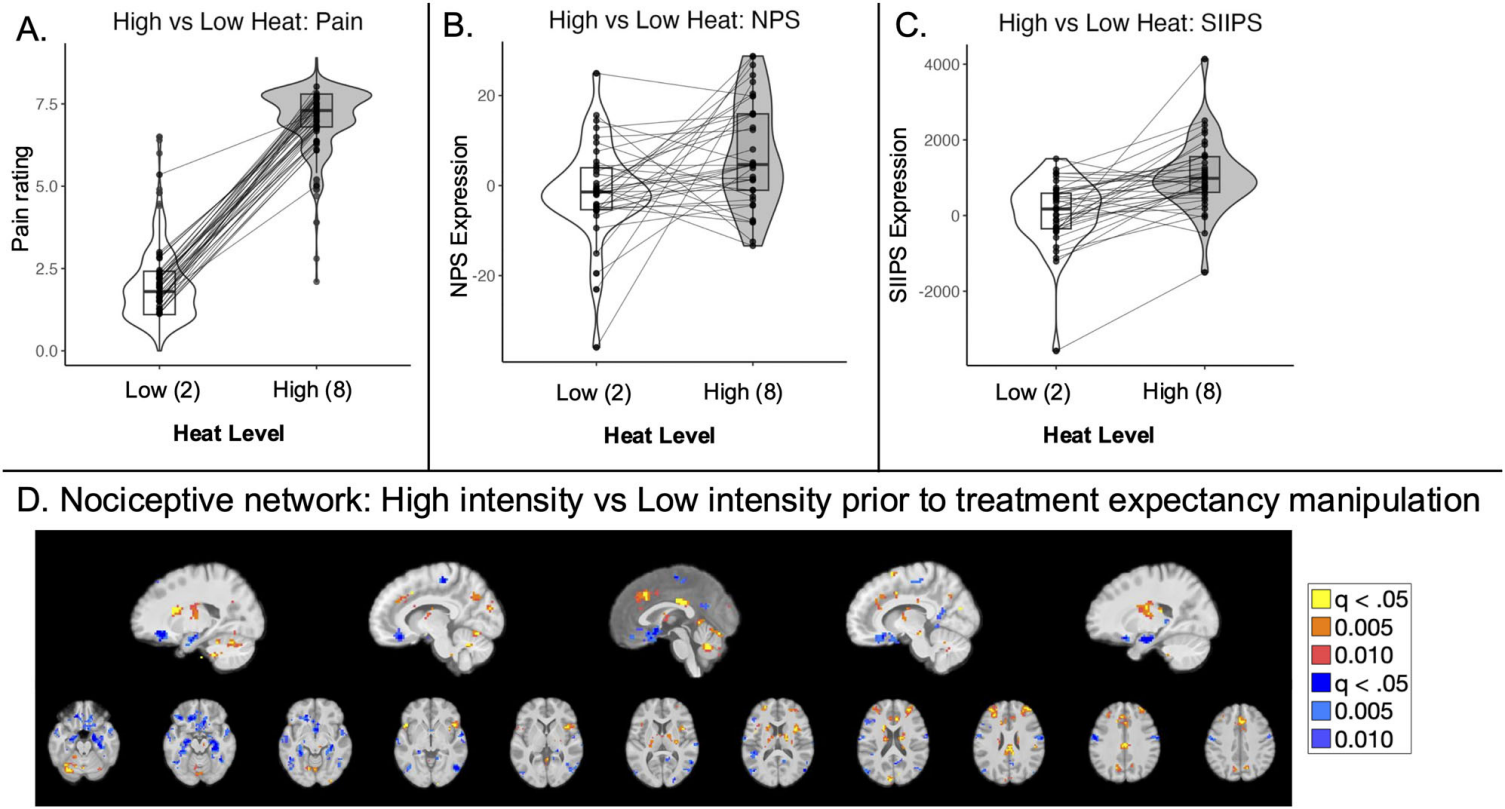
Stimulus intensity effects prior to treatment expectancy manipulation. We compared responses to temperatures calibrated to elicit low pain (Level 2) and high pain (Level 8) prior to the treatment expectancy manipulation to isolate effects of changes in noxious stimulus intensity. ***A***, All participants reported higher pain in response to high-intensity stimulation. ***B***, There was a significant effect of the heat level on expression of the NPS (Wager et al., 2013). ***C***, We also observed significant effects of the heat level on the SIIPS (Woo et al., 2017). ***D***, A voxelwise contrast between high- and low-intensity heat prior to the treatment expectancy manipulation was used to localize nociceptive network responses in the current sample (Table 2), consistent with our preregistration (https://aspredicted.org/IIX_EQG). See Extended Data Figure 3-1 for uncorrected voxelwise results and Extended Data Figures 3-2 and 3-3 for results across all blocks. Extended Data Figure 3-4 reports all preregistered analyses and hypotheses, how they were implemented in the current work, deviations from preregistration, and whether hypotheses were supported.

To isolate brain regions that were sensitive to noxious input, we contrasted high heat and low heat trials prior to the treatment manipulation. We observed a significant effect of the heat level on pattern expression for both NPS (*t*_(36)_ = 2.82; *p* = 0.008; CI = [2.32, 14.43]; Fig. 3*B*) and SIIPS (*t*_(36)_ = 5.56; *p* < 0.001; CI = [626.07, 1,345.78]; Fig. 3*C*). Voxelwise whole-brain correction (FDR *q* < 0.05) revealed positive associations (high heat > low heat) in the bilateral anterior insula/operculum, right SII, left middle insula, bilateral thalamus, rACC, dorsal ACC (dACC), posterior cingulate cortex, bilateral DLPFC, bilateral dorsomedial PFC (DMPFC), bilateral cerebellum, and right midbrain near the substantia nigra (Table 2). Negative associations (low heat > high heat) were present in the medial OFC (mOFC)/VMPFC, left VLPFC, bilateral middle and superior temporal gyrus, bilateral hippocampus, SMA, right motor cortex, bilateral occipital lobe, and bilateral primary somatosensory cortex (Table 2). Consistent with our preregistration, this contrast was used as a localizer in subsequent analyses to evaluate expectancy effects on responses within pain-processing regions. Uncorrected voxelwise results are reported in Extended Data Figure 3-1 for completeness and inclusion in future meta-analyses. Results were similar when we evaluated responses across all blocks, i.e., including trials following the treatment expectancy manipulation (Extended Data Figs. 3-2, 3-3).

10.1523/JNEUROSCI.0050-25.2025.f3-1 Figure 3-1 Effects of noxious stimulation on heat-evoked responses prior to treatment: Uncorrected results and correction within regions of interest. Download Figure 3-1, DOCX file.

10.1523/JNEUROSCI.0050-25.2025.f3-2 Figure 3-2 *Stimulus intensity effects across all runs.* Our main manuscript compares low and high heat stimulation prior to the treatment manipulation to isolate nociceptive networks. Results were similar when we measured effects across all runs. A) All subjects reported increases in pain as a function of stimulus intensity. B) There was a significant effect of stimulus intensity on NPS and SIIPS expression across the entire task. C) Regions that showed differences between high and low intensity stimulation were similar whether we included all runs in analyses (see Extended Data Figure 3-3 for complete results) or measured responses only prior to the treatment manipulation, as reported in the main manuscript. Download Figure 3-2, TIF file.

10.1523/JNEUROSCI.0050-25.2025.f3-3 Figure 3-3 Effects of noxious stimulation on heat-evoked responses across all blocks. Download Figure 3-3, DOCX file.

**Figure 4.**
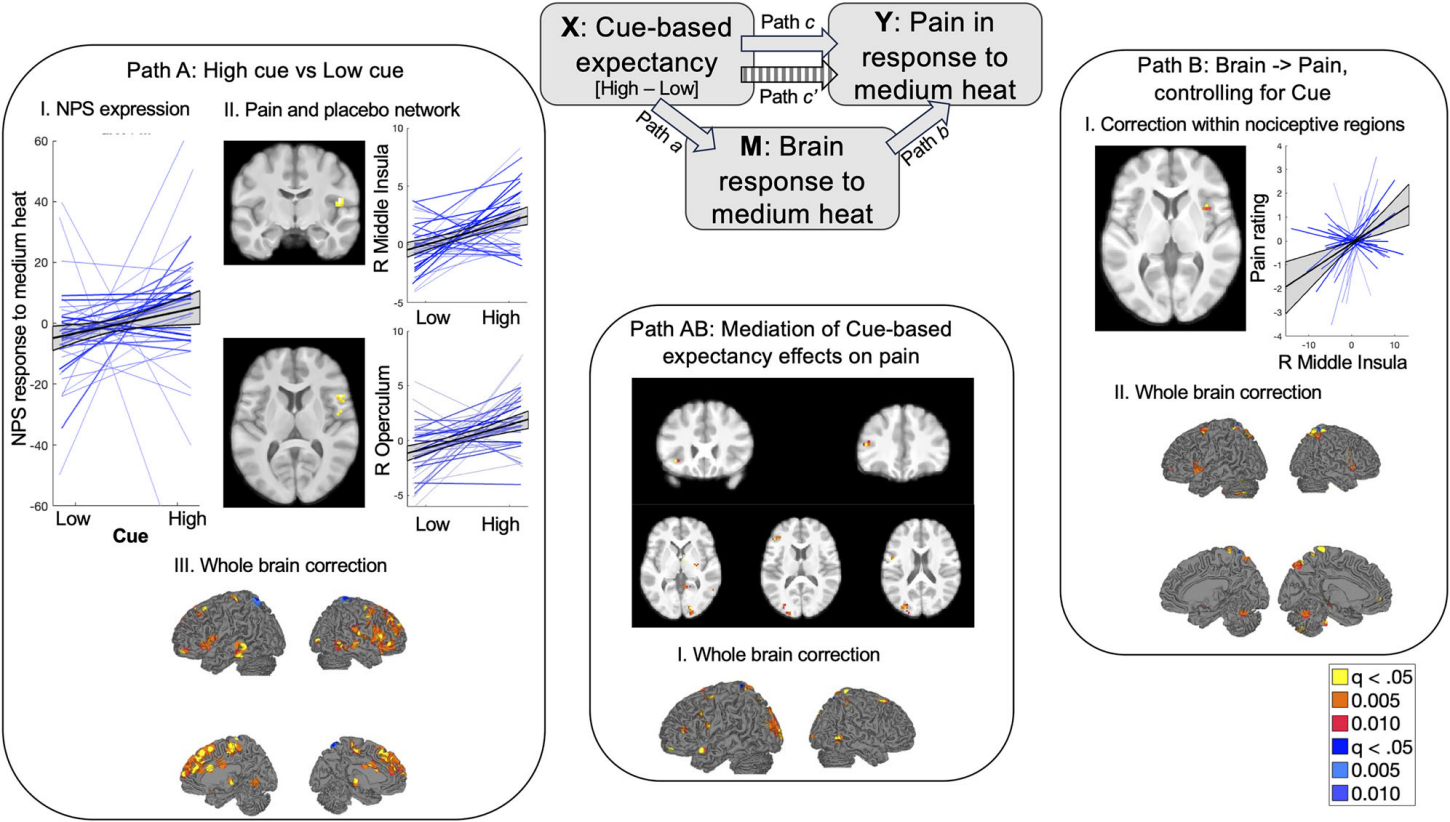
Brain mediators of cue-based stimulus expectancy effects in the absence of treatment expectancy. We used voxelwise multilevel mediation to isolate effects of cue-based stimulus expectancies on heat-evoked brain responses on medium heat trials. Top middle, Mediation path diagram. Cue (high > low) was treated as the input variable (*X*), pain on medium trials was treated as the dependent variable (*Y*), and we searched for voxelwise mediators of cue effects on pain. Left, Path A identifies effects of cues on heat-evoked brain activation. We observed significant Path a effects on the NPS (***I***) and on responses in the right dorsal posterior insula and operculum within regions involved in pain and placebo (***II***). Whole-brain correction revealed substantial cue effects in the bilateral insula, dACC, DLPFC, and other regions (Table 3). Bottom middle, Dynamic cue effects on pain were mediated by the DLPFC, lateral and ventrolateral PFC, caudate, and other regions (Table 3). We did not observe significant mediation by the NPS, SIIPS, or nociceptive networks. Right, Path B identifies regions that are associated with pain when controlling for cue. Within nociceptive regions, we observed positive associations in the right middle insula. Whole-brain correction identified associations in the striatum, anterior insula, and DMPFC, among other regions (Table 3). There were no associations with NPS or SIIPS expression. For complete results, see Table 3 and Extended Data Figure 4-1.

10.1523/JNEUROSCI.0050-25.2025.f3-4 Figure 3-4 Preregistered neuroimaging analyses and implementation. Download Figure 3-4, DOCX file.

**Table 2.** Effects of noxious stimulation on heat-evoked responses prior to treatment^a^

Contrast	Anatomical label	*x*	*y*	*z*	# of voxels	Volume (mm^3^)	Maximum *Z* statistic
High > low	L Cerebellum VI	−26	−64	−28	555	14,985	15.47
	R Cerebellum X	26	−38	−44	11	297	9.64
	R Cerebellum Crus 1	38	−62	−28	93	2,511	12.26
	L Cerebellum IV–V	−26	−32	−34	3	81	7.56
	L Cerebellum IV–V	−20	−44	−26	16	432	8
	R Midbrain (substantia nigra pars compacta)	10	−22	−14	8	216	7.67
	Area 44	−50	16	−4	29	783	11.98
	R Inferior Occipital Gyrus	32	−94	−10	4	108	7.93
	R IFG p. Opercularis	44	14	4	135	3,645	14.56
	Cerebellar Vermis 4/5	−2	−52	2	17	459	9.18
	Right thalamus/Caudate	16	−4	14	110	2,970	15.38
	Right thalamus/Insula	28	−16	8	19	513	8.52
	Thal: Premotor	−20	−14	14	71	1,917	7.57
	R Middle Frontal Gyrus	26	52	4	7	189	7.43
	L IFG p. Triangularis	−38	20	10	11	297	8.72
	L IFG p. Opercularis	−44	8	10	5	135	8.02
	L ACC	−4	22	28	244	6,588	13.08
	L Middle Frontal Gyrus	−32	46	26	141	3,807	13.22
	R Middle Frontal Gyrus	32	50	22	146	3,942	10.16
	L Cuneus	−8	−92	22	32	864	11.58
	Right Thalamus	20	−28	20	6	162	7.36
	Posterior Cingulate Cortex	2	−28	28	83	2,241	11.04
	R SupraMarginal Gyrus (SII)	52	−26	26	7	189	7.34
	L Precuneus	−10	−70	34	45	1,215	7.38
	R Precuneus	14	−64	34	16	432	7.78
	R SupraMarginal Gyrus	58	−34	50	35	945	8.41
	R Posterior-Medial Frontal (DMPFC)	14	10	68	23	621	8.75
Low > high	R Hippocampus/amygdala	38	−8	−22	556	15,012	20
	L Middle Temporal Gyrus	−50	8	−26	169	4,563	12.34
	L Hippocampus	−28	−22	−16	148	3,996	14.02
	L Mid Orbital Gyrus	−4	26	−14	376	10,152	13.64
	R Middle Temporal Gyrus	52	−64	−2	175	4,725	10.49
	L Middle Occipital Gyrus	−50	−74	4	148	3,996	9.08
	L Middle Temporal Gyrus	−64	−14	−8	19	513	8.84
	R Precuneus	10	−52	14	56	1,512	7.75
	R Hippocampus	22	−34	4	7	189	8.26
	R Superior Temporal Gyrus	68	−22	8	14	378	7.74
	R Superior Occipital Gyrus	22	−94	8	7	189	7.67
	L Superior Temporal Gyrus	−46	−40	16	30	810	7.55
	L Postcentral Gyrus	−56	−10	34	141	3,807	12.43
	L IFG p. Triangularis (DLPFC)	−46	26	20	46	1,242	8.55
	R Postcentral Gyrus	62	−8	28	71	1,917	10.75
	L IFG p. Triangularis	−38	14	26	7	189	7.62
	RPrecentral Gyrus	44	−16	56	94	2,538	10.2
	L Postcentral Gyrus	−38	−38	56	24	648	9.74
	L Superior Frontal Gyrus (DMPFC)	−22	32	56	12	324	8.97
	L Paracentral Lobule	−2	−26	56	87	2,349	14.4

a This table presents whole-brain FDR–corrected results using robust regression for the contrast (high heat > low heat) during the first three runs, prior to the treatment manipulation. See Extended Data Figure 3-1 for uncorrected results and correction within a priori regions involved in pain and placebo and Extended Data Figures 3-2 and 3-3 for results including treatment blocks.

#### Brain mediators of cue-based stimulus expectancy effects on pain

We next asked which brain regions mediated pure stimulus expectancy effects on pain by using mediation analysis restricted to medium heat trials prior to the treatment expectancy manipulation (see Fig. 4 for the path diagram). Path *a* of the analysis isolates stimulus expectancy effects on brain responses to medium heat, i.e., regions whose response to medium heat varies as a function of the cue that proceeded it. Small volumes corrected results are displayed in Figure 4 and Table 3; voxelwise uncorrected results are reported in Extended Data. We observed significant Path *a* effects on NPS expression (*a* = 1.93; *p* < 0.001), but not SIIPS expression (*p* > 0.6). Correction within regions involved in pain and placebo revealed positive Path *a* effects (HM > LM) in the right middle insula/SII and right operculum, and extracting from these clusters revealed that the right operculum also mediated cue effects on pain during baseline blocks. Correction within regions that differentiated between high- and low-intensity stimuli in the present sample further revealed positive Path *a* effects in the DMPFC and right preSMA (Table 3). Finally, whole-brain FDR correction additionally revealed positive Path *a* effects in the bilateral anterior insula, dACC, right SII, bilateral DLPFC, and bilateral subthalamic nuclei (Fig. 4, Table 3). There were no regions that showed negative Path *a* effects (i.e., low > high) based on correction within a priori regions or whole-brain search.

10.1523/JNEUROSCI.0050-25.2025.f4-1 Figure 4-1 Brain mediators of cue-based stimulus expectancies: Uncorrected results and correction within regions of interest. Download Figure 4-1, DOCX file.

**Table 3.** Brain mediators of cue-based stimulus expectancies^a^

Contrast	Anatomical label	*x*	*y*	*z*	# of voxels	Volume (mm^3^)	Maximum *Z* statistic
Path A positive	L Inferior Temporal Gyrus	−68	−32	−20	10	270	11.49
	R Insula Lobe extending to R DLPFC	40	26	−4	121	3,267	13.61
	R Middle Temporal Gyrus	58	−50	−4	41	1,107	13.93
	R Superior Temporal Gyrus	58	−22	−4	43	1,161	14.95
	L IFG p. Orbitalis	−38	52	−14	10	270	10.7
	Area Id1	−40	−14	−10	1	27	9.91
	R Lingual Gyrus/V1	16	−64	2	32	864	12.14
	L Middle Temporal Gyrus	−70	−38	−4	8	216	15.41
	L IFG p. Triangularis/Anterior insula	−44	16	2	58	1,566	11.51
	Bilateral subthalamic nuclei	2	−22	−2	16	432	10.36
	R IFG p. Opercularis/BA44	52	14	14	119	3,213	12.9
	R Caudate Nucleus	14	16	2	32	864	10.42
	R Middle Occipital Gyrus (Area hOc4lp)	40	−92	4	2	54	10.32
	R Pallidum/Putamen	22	4	4	10	270	12.75
	R Rolandic Operculum (Area OP3 [VS])	46	−10	14	107	2,889	12.47
	R Insula Lobe	34	22	8	34	918	14.3
	R Middle Frontal Gyrus (DLPFC)	32	44	28	89	2,403	10.86
	L Superior Medial Gyrus (DMPFC)	2	38	38	349	9,423	15.01
	R ACC (BA33)	4	16	22	14	378	19.81
	L MCC	−8	8	32	13	351	14.34
	R Superior Frontal Gyrus (DMPFC)	20	58	32	23	621	10.16
	R MCC	8	−2	34	26	702	10.51
	RPrecentral Gyrus	38	2	50	88	2,376	13.05
	R Posterior-Medial Frontal	8	−22	58	87	2,349	16.97
	R Superior Frontal Gyrus (DMPFC)	20	20	46	14	378	14.04
	L Middle Frontal Gyrus (DLPFC)	−38	26	50	13	351	11.03
	L Paracentral Lobule	−14	−26	68	13	351	13.7
Path A negative	None						
Path B positive	L Cerebellum VII	−26	−68	−44	25	675	17.01
	Left Pons	−14	−32	−34	7	189	10.13
	Left Temporal Pole	−62	−16	−34	1	27	11.14
	L Cerebellum VI	−8	−64	−26	24	648	9.94
	L Cerebellum VI	−22	−62	−22	7	189	11.18
	R Fusiform Gyrus	34	−46	−20	4	108	10.96
	R Putamen	16	8	−8	18	486	13.24
	L Anterior insula extending to putamen, inferior frontal gyrus	−22	26	−4	32	864	11.64
	Thal: Parietal	−20	−26	−4	9	243	11.1
	L Putamen extending to anterior insula, ventral striatum	−28	10	4	93	2,511	15.77
	L Superior Orbital Gyrus	−22	52	−2	11	297	12.3
	R Insula Lobe	38	4	10	32	864	13.29
	R IFG p. Opercularis (BA44)	50	8	20	30	810	10.07
	R Middle Frontal Gyrus (DMPFC)	28	10	44	5	135	13.15
	L Precuneus	−10	−74	52	13	351	11.62
	L Middle Frontal Gyrus (DMPFC)	−26	2	56	46	1,242	11.15
	R Superior Parietal Lobule (Area 7A SPL)	26	−58	68	43	1,161	32.75
	L Superior Parietal Lobule (Area 5L)	−20	−52	68	5	135	10.06
	R Postcentral Gyrus (BA1)	32	−44	70	9	243	28.93
	L Superior Parietal Lobule	−28	−50	70	1	27	10.12
	R Superior Parietal Lobule	20	−52	74	6	162	11.13
Path B negative	None						
Path AB positive	L Cerebellum IX	−2	−50	−38	7	189	10.05
	Cerebellar Vermis 9	2	−58	−34	2	54	22.57
	Left middle temporal gyrus	−58	10	−16	2	54	12.47
	L Middle Orbital Gyrus	−34	56	−14	1	27	10.05
	R Putamen	32	−4	−8	5	135	11.01
	L IFG p. Orbitalis	−38	26	−8	2	54	10.45
	R Middle Temporal Gyrus	58	−56	−2	7	189	11.26
	R Lingual Gyrus	8	−50	2	26	702	10.73
	R Calcarine Gyrus/V1	16	−92	2	24	648	12.05
	R putamen contiguous with thalamus and insula	28	−14	2	6	162	10.08
	Thal: Temporal	4	−8	2	1	27	10.86
	R BNST	4	4	2	1	27	10.09
	L Middle Occipital Gyrus/Area hOc3d [V3d]	−22	−98	14	31	837	10.95
	L Middle Occipital Gyrus/Area hOc4lp	−32	−86	20	30	810	9.92
	L IFG p. Triangularis (DLPFC)	−44	34	14	7	189	10.46
	L Precentral Gyrus/BA44 (DLPFC)	−50	2	20	5	135	11.54
	L SupraMarginal Gyrus (SII)	−46	−34	28	1	27	11.19
	L Precentral Gyrus (DLPFC)	−46	8	38	11	297	10.9
	L Superior Occipital Gyrus/Area hOc4d [V3A]	−14	−88	40	10	270	12.94
	R Superior Parietal Lobule/Area hIP3 IPS	34	−46	56	14	378	12.3
	L Precuneus/Area 7A SPL	−14	−68	58	3	81	11.62
	L Posterior-Medial Frontal	−2	14	64	32	864	10.76
	L Precuneus/Area 7A SPL	−8	−74	62	1	27	12.8
	L Precentral Gyrus	−38	−2	62	9	243	12.47
	R Superior Frontal Gyrus	28	20	62	10	270	12.34
	R Precuneus/Area 5L SPL	10	−56	70	11	297	14.36
Path AB neg	None						

a This table reports results of voxelwise multilevel mediation searching for mediators of the association between pain-predictive cue [X, (high > low)] and pain on medium heat trials prior to the treatment manipulation. Whole-brain FDR correction was implemented using the mediation-moderation toolbox (Wager et al., 2009 ); for correction within nociceptive regions and a priori regions involved in pain and placebo, as well as uncorrected results, see Extended Data Figure 4-1.

Path *b* isolates regions in which variations in trial-by-trial activation were associated with pain while controlling for cue. We did not observe any Path *b* effects on NPS or SIIPS expression (all *p*'s > 0.2) or when we corrected within regions based on prior studies of pain and placebo. However, we observed Path *b* effects in the right middle insula based on correction within nociceptive regions in the current sample (Fig. 4). Whole-brain FDR correction additionally identified the bilateral striatum (encompassing the ventral striatum and putamen), cerebellum, bilateral anterior insula, bilateral DMPFC, bilateral superior parietal lobule, and the right DLPFC (Table 3, Fig. 4). No regions were inversely related to pain when controlling for cue.

Finally, we identified voxelwise mediators of stimulus expectancy effects on pain. We did not observe significant mediation by the NPS or SIIPS (all *p*'s > 0.4) nor by regions within placebo or nociceptive networks. Whole-brain FDR correction revealed significant positive mediation by the left DLPFC, left lateral PFC, left ventrolateral PFC, right caudate, and other regions (Fig. 4, Table 3). There were no negative mediators.

#### Treatment-based expectancy effects on heat-evoked brain responses

**Figure 5.**
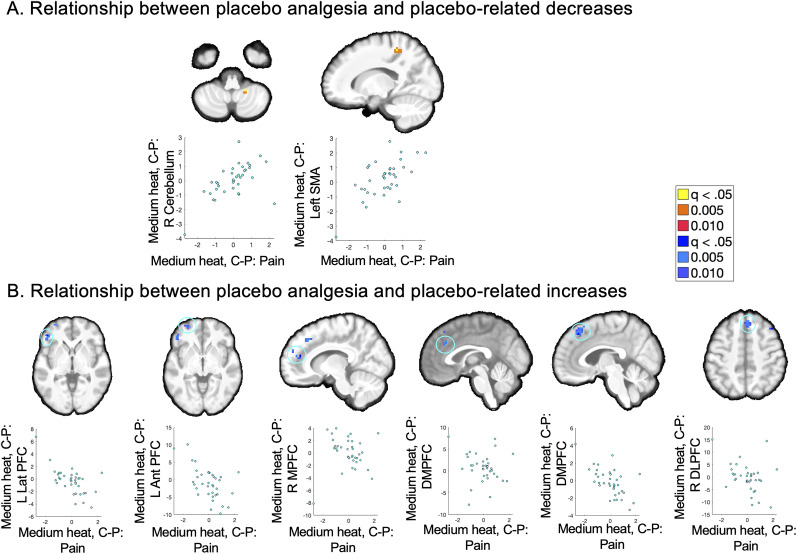
Treatment-based expectancy effects on medium heat trials. To isolate pure effects of treatment expectancy, we compared responses to uncued medium heat on placebo blocks with placebo blocks using robust regression, which controlled for order and evaluated associations with reported placebo analgesia. Only associations with placebo analgesia survived whole-brain multiple–comparison corrections (Table 4); see Extended Data Figure 5-1 for uncorrected results and main effects regardless of placebo analgesia. Results across all temperatures are reported in Extended Data Figures 5-2 and 5-3. ***A***, Relationship between placebo analgesia and placebo-related decreases. We observed positive associations between placebo analgesia and activation in the right cerebellum and precuneus, such that participants who reported larger reductions in pain on placebo blocks relative to control blocks also showed larger reductions in activation in these regions on placebo blocks relative to control blocks. ***B***, Relationship between placebo analgesia and placebo-related increases. We observed negative associations in frontal regions including rACC, VLPFC, DMPFC, and DLPFC, such that individuals who reported stronger placebo analgesia exhibited larger increases in activation in these regions on placebo blocks, relative to control blocks, consistent with downmodulation of pain.

We next examined treatment effects on pain-evoked brain responses. Consistent with our preregistration, the magnitude of the treatment expectancy effect on pain on uncued trials (control–placebo) was included as a between-subject moderator, which captures the substantial individual differences in placebo analgesia that we observed. We focus on analyses of placebo effects on uncued medium heat trials due to interactions with the heat level; analyses across temperatures are reported in Extended Data. Supporting the importance of individual differences, only the moderation effect survived whole-brain FDR correction. We observed positive associations in the right cerebellum and left precuneus (Fig. 5*A*; Table 4), driven by reductions with placebo (i.e., control > placebo) that scaled with the magnitude of placebo analgesia. Regions with negative associations, including the rACC, right DMPFC, left lateral PFC, left VLPFC, DMPFC, and right DLPFC (Fig. 5*B*; Table 4), were driven by placebo-related increases (i.e., placebo > control) that were correlated with the magnitude of placebo-based reductions in pain, consistent with modulatory effects and placebo-based downregulation. There were no relationships between the magnitude of placebo analgesia and treatment expectancy effects on signature pattern expression (all *p*'s > 0.3). Main effects and uncorrected results are reported in Extended Data.

10.1523/JNEUROSCI.0050-25.2025.f5-1 Figure 5-1 Treatment expectancy effects on brain responses to medium heat and associations with placebo analgesia. Download Figure 5-1, DOCX file.

10.1523/JNEUROSCI.0050-25.2025.f5-2 Figure 5-2 *Placebo effects across temperatures.* Our main analyses focus on placebo effects on uncued medium heat trials, to control for changes in temperature. Here, we present results on uncued trials across all temperatures. *Top:* The only effect that survived multiple comparisons correction was a main effect of Treatment Expectancy in the left caudate, driven by higher activation on placebo blocks, relative to control blocks. Uncorrected main effects (middle) and associations (bottom) are reported in Extended Data Figure 5-3. Download Figure 5-2, TIF file.

10.1523/JNEUROSCI.0050-25.2025.f5-3 Figure 5-3 Treatment expectancy effects across temperatures. Download Figure 5-3, DOCX file.

**Table 4.** Associations between placebo analgesia and treatment expectancy effects on brain responses^a^

Contrast	Anatomical label	*x*	*y*	*z*	# of voxels	Volume (mm^3^)	Maximum *Z* statistic
Positive association	R Cerebellum IX	20	−46	−50	4	108	16.43
	L Precuneus (Area 3A)	−16	−40	56	22	594	15.25
Negative association	L IFG p. Orbitalis	−46	38	−4	23	621	12.05
	L Superior Frontal Gyrus (AntPFC)	−32	62	−2	37	999	11.62
	R Superior Medial Gyrus (MPFC)	10	50	16	24	648	11.62
	L Superior Medial Gyrus (DMPFC)	−2	32	32	8	216	12.09
	R Superior Medial Gyrus (DMPFC)	4	32	50	51	1,377	13.79
	R Middle Frontal Gyrus (DLPFC)	44	26	50	3	81	11.6

a This table presents whole-brain FDR-corrected results of robust regression evaluating associations between pure treatment expectancy effects on heat-evoked activation [uncued medium trials, (control–placebo)] and the magnitude of placebo analgesia (controlling for the counterbalanced order). No regions survived correction within nociceptive regions or a priori regions involved in pain and placebo. See Extended Data Figure 5-1 for uncorrected results and main effects controlling for individual differences in placebo analgesia and Extended Data Figures 5-2 and 5-3 for results across all temperatures.

**Figure 6.**
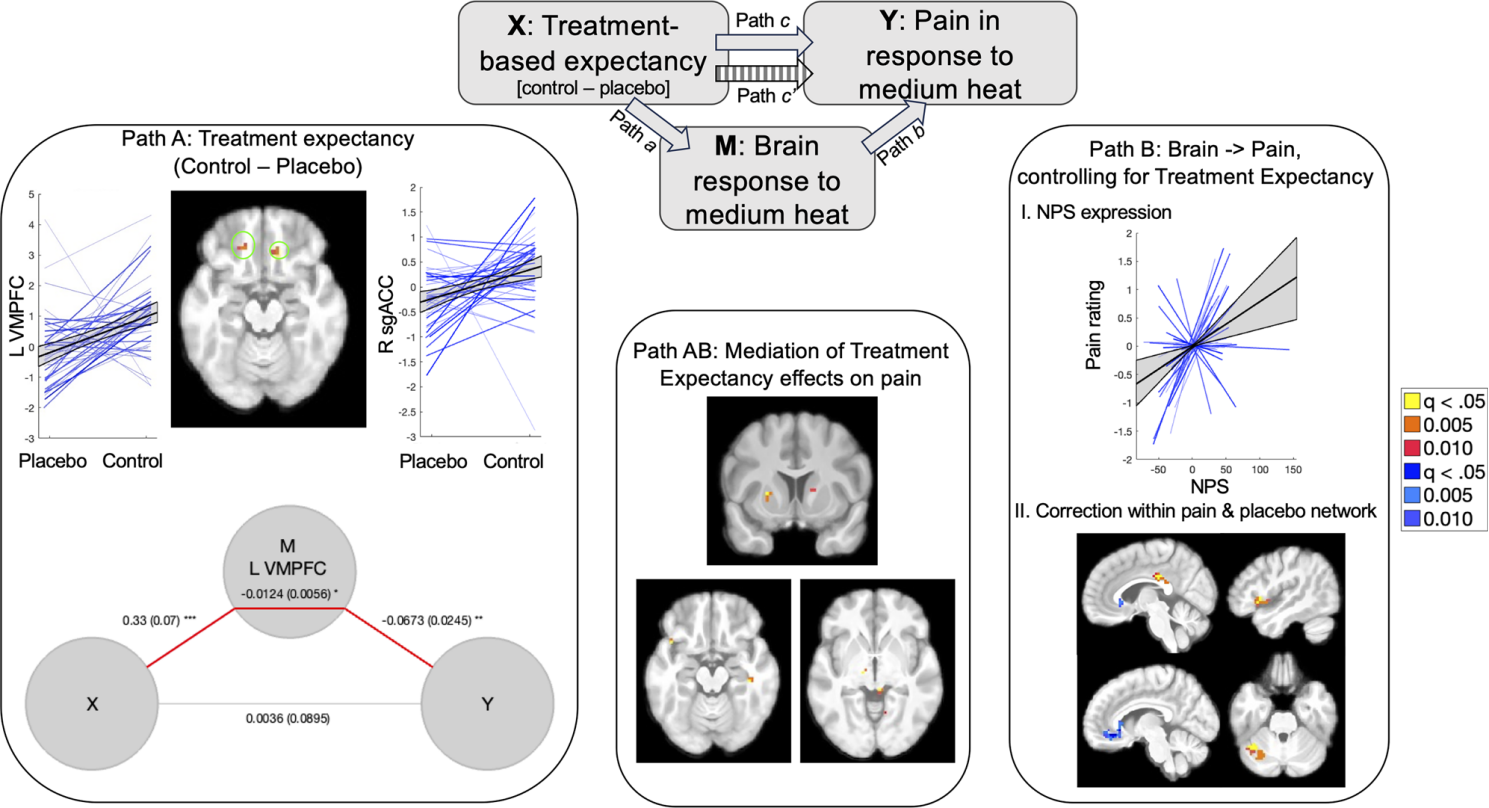
Mediation of treatment expectancy effects on pain. Top, Mediation framework. Consistent with our preregistration, we used voxelwise multilevel mediation to examine dynamic mediators of treatment expectancy effects (*X*, control > placebo) on pain in response to medium heat. We focused on uncued trials to isolate pure expectancy effects. Left, Path A: Treatment expectancy*.* Correction within regions involved in nociception revealed significant Path a effects (control > placebo) in the left VMPFC/OFC and right sgACC, driven by stronger activation during control blocks than placebo blocks. Extracting from the VMPFC cluster region revealed that it also mediated dynamic associations between treatment expectancy and subjective pain, as depicted in the path diagram. Additional results that survived whole-brain correction are reported in Table 5. Right, Path B: brain→pain controlling for treatment expectancy. Path b identifies regions whose activation is associated with pain when controlling for treatment expectancy. We observed significant Path b effects on NPS expression, as well as within nociceptive regions and a priori regions involved in pain and placebo (Table 5). Bottom, Path AB: mediation of treatment expectancy effects on pain. We observed significant mediation of dynamic associations between treatment expectancy and pain in the left putamen, right caudate, midbrain near the PAG, and other regions (Table 5). Participants who showed stronger treatment expectancy effects on these regions also showed stronger associations between activation and subjective pain, when controlling for expectancy. For uncorrected results, see Extended Data Figure 6-1.

We also evaluated placebo effects using mediation analysis on uncued medium heat trials, regardless of individual differences in placebo analgesia, which allowed us to search for mediators of treatment expectancy effects on medium uncued trials. As illustrated in Figure 6, Path *a* isolates the main effect of treatment expectancy (control > placebo). We did not observe Path *a* effects on the NPS, SIIPS, or within a priori regions involved in pain and placebo. However, we observed significant Path *a* effects within this sample's nociceptive network in left VMPFC and right mOFC/sgACC (Fig. 6*B*), driven by elevated activation for control administration relative to placebo administration (Table 5). When we extracted responses from each cluster, we observed that the left VMPFC also showed significant Path *b* effects and mediation. Whole-brain FDR correction additionally revealed positive Path *a* effects in the right VMPFC, left middle cingulate, and left posterior cingulate (Table 5); there were no regions that showed negative associations (i.e., increases with placebo).

10.1523/JNEUROSCI.0050-25.2025.f6-1 Figure 6-1 Voxel-wise mediators of treatment expectancy effects on pain: Uncorrected results. Download Figure 6-1, DOCX file.

**Table 5.** Voxelwise mediators of treatment expectancy effects on pain^a^

Correction	Contrast	Anatomical label	*x*	*y*	*z*	# of voxels	Volume (mm^3^)	Maximum *Z* statistic
Whole-brain FDR correction	Path A pos	L Cerebellum, contiguous with brainstem	−16	−28	−46	14	378	12.68
		L IFG p. Orbitalis (Area Fo3) /mOFC/sgACC	−22	22	−22	12	324	10.8
		R Rectal Gyrus (Area Fo2/SgACC)	10	32	−16	16	432	12.34
		R Fusiform Gyrus	34	−76	−16	6	162	10.95
		L Middle Orbital Gyrus	−26	34	−14	34	918	10.86
		R Rectal Gyrus/sgACC, contiguous with striatum	16	16	−14	4	108	11.72
		R Middle Frontal Gyrus/DLPFC	34	34	22	3	81	9.77
		L Posterior Cingulate Cortex	−10	−28	28	12	324	10.25
		L S1	−62	−32	44	19	513	10.8
	Path A neg	Nothing survives						
	Path B pos	L Cerebellum VII	−22	−74	−52	127	3,429	10.51
		R Cerebellum VIII	32	−70	−52	88	2,376	11.36
		R Cerebellum Crus 2	8	−88	−32	14	378	10.47
		L Cerebellum Crus 2	−8	−86	−28	52	1,404	9.8
		L Cerebellum VI	−38	−62	−26	108	2,916	11.1
		R Cerebellum Crus 1	40	−64	−28	36	972	18.67
		Area Fo2/mOFC	10	28	−28	16	432	12.26
		R Middle Orbital Gyrus (Area Fp1)/VMPFC	20	62	−16	19	513	11.14
		Thal: Prefrontal	10	−2	4	13	351	9.79
		R Precuneus	10	−68	28	150	4,050	10.74
		L Caudate Nucleus	−16	8	16	23	621	10.75
		R Rolandic Operculum (Area PFop (IPL))	58	−16	20	31	837	11.09
		R Putamen, contiguous with middle insula	28	2	14	23	621	10.07
		L Middle Frontal Gyrus (DLPFC)	−44	38	16	10	270	9.96
		R IFG p. Opercularis (Area 44)	58	10	16	11	297	18.85
		Posterior Cingulate Cortex	2	−28	28	93	2,511	11.04
		L Caudate	−20	−4	22	12	324	11.08
		R Middle Frontal Gyrus (DLPFC)	38	22	44	206	5,562	12.08
		L MCC	−2	4	34	37	999	12.26
		R Superior Frontal Gyrus	20	58	32	28	756	10.42
		L Inferior Parietal Lobule (Area hIP1 (IPS)	−38	−56	46	340	9,180	15.44
		R MCC	10	16	40	24	648	10.2
		R Inferior Parietal Lobule	46	−46	52	128	3,456	13.86
		L Posterior-Medial Frontal (DMPFC)	−8	14	58	306	8,262	12.98
		L Precuneus (Area 7A (SPL))	−8	−68	64	6	162	11.58
		R Postcentral Gyrus (Area 1)	28	−38	74	7	189	11.59
		L Superior Parietal Lobule	−26	−50	74	4	108	14.45
	Path B neg	Nothing survives						
	Path AB pos	L Cerebellum VIII	−32	−62	−56	3	81	10.67
		L Temporal Pole	−44	20	−16	3	81	9.92
		Midbrain surrounding PAG	14	−62	−2	6	162	10.27
		Thal: Premotor	4	−34	−4	3	81	9.93
		R Lingual Gyrus (Area hOc2 [V2])	−14	−16	−4	3	81	9.78
		L Putamen	−26	10	2	10	270	9.89
		R Calcarine Gyrus (Area hOc1 [V1])	26	−70	4	21	567	9.97
		R Caudate Nucleus	16	14	8	12	324	10.99
		R Calcarine Gyrus (Area hOc3d [V3d])	10	−74	16	28	756	10.67
		L Cuneus (Area hOc3d [V3d])	−10	−74	22	7	189	10.19
		L Superior Parietal Lobule (Area 7A (SPL))	−34	−64	52	4	108	11.81
		L Precuneus (Area 7P (SPL))	−4	−74	58	5	135	10.64
		L Superior Parietal Lobule (Area 7A (SPL))	−22	−68	68	1	27	21.08
	Path AB neg	Nothing survives						
FDR correction within nociceptive regions	Path A pos	R Rectal Gyrus/sgACC/mOFC	10	32	−16	6	162	9.66
		L Superior Orbital Gyrus (Area Fo3)/VMPFC	−22	34	−14	19	513	10.41
	Path A neg	Nothing survives						
	Path B pos	L Cerebellum VI	−34	−62	−26	68	1,836	11.1
		R Cerebellum Crus 1	38	−62	−28	20	540	10.51
		L Caudate	−20	10	16	7	189	10.75
		Posterior Cingulate Cortex	2	−26	28	51	1,377	11.04
		R Precuneus	14	−64	34	8	216	10.74
	Path B neg	Nothing survives						
	Path AB pos	Nothing survives						
	Path AB neg	Nothing survives						
Cluster correction within placebo ROIs	Path A pos	Nothing survives						
	Path A neg	Nothing survives						
	Path B pos	L Cerebellum VI	−38	−56	−28	15	405	10.61
		R Anterior Insula	50	16	−4	13	351	8.52
		R Putamen	26	2	14	9	243	10.07
		Posterior Cingulate Cortex	2	−26	28	21	567	9.84
	Path B neg	L ACC (Area s32)	−8	32	−10	15	405	8.3
	Path AB pos	Nothing survives						
	Path AB neg	Nothing survives						

a This table reports results of voxelwise multilevel mediation searching for mediators of the dynamic trial-by-trial association between treatment expectancy [*X*, (control > placebo)] and pain on uncued medium heat trials. Uncorrected results are reported in Extended Data Figure 6-1.

We observed positive Path *b* effects (positive associations between brain activation and pain, controlling for treatment) on the NPS, but not the SIIPS (Fig. 6*C*; Table 5). Correction within regions involved in pain and placebo revealed positive Path *b* effects in the left cerebellum, right anterior insula, and posterior cingulate and negative associations in the left VMPFC/sgACC (Fig. 6*C*). Correction within nociceptive regions in the current sample additionally revealed positive associations in the bilateral cerebellum, left caudate, and right occipital cortex. Whole-brain correction confirmed these findings and revealed several other regions (Table 5).

Finally, we searched for mediators of the trial-by-trial relationship between treatment expectancy and pain. We did not observe mediation by NPS or SIIPS nor within our a priori networks involved in pain and placebo or nociception. Whole-brain correction revealed that effects were positively mediated by the left anterior insula, bilateral striatum, and midbrain near the PAG, among other regions (Fig. 6*D*; Table 5). All regions were driven by the covariance between paths, meaning that individuals who showed stronger Path *a* effects on these regions also showed stronger Path *b* effects, thus contributing to the overall behavioral relationship between treatment expectancy and pain.

We also conducted a moderated mediation analysis that included a moderator for individual differences in placebo analgesia. Moderation did not survive multiple-comparison correction in any path; for uncorrected results, please see Extended Data.

#### Evaluating relationships between stimulus expectancies and treatment expectancies: main effects and interactions

Our main goal was to evaluate the relationship between cue-based stimulus expectancies and placebo-based treatment expectancies. We did this in two ways: (1) by focusing on trials in which stimulus expectancies were crossed with treatment expectancies and testing for formal interactions (consistent with our behavioral analysis) and (2) by testing whether pure stimulus expectancies (i.e., cue-based expectancy effects prior to treatment) and pure treatment expectancies (i.e., treatment expectancy effects on uncued trials) rely on shared or dissociable brain mechanisms.

**Figure 7.**
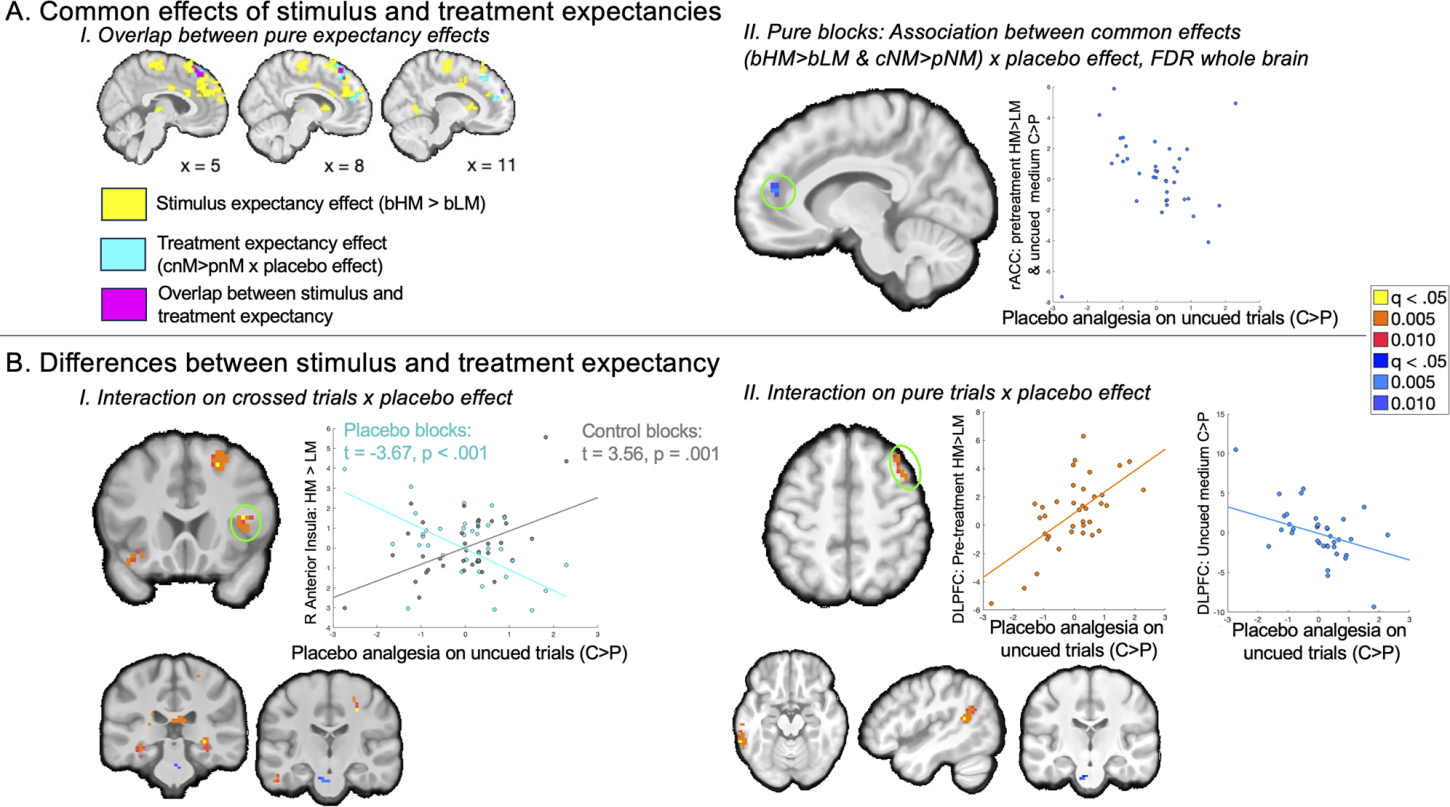
Stimulus expectancy versus treatment expectancy. ***A***, Common effects of stimulus and treatment expectancies. We tested whether there were any common effects of stimulus and treatment expectancies by evaluating blocks of pure stimulus expectancies (i.e., cue effects prior to the treatment manipulation) and pure treatment expectancies (i.e., comparisons between placebo and control on uncued runs that scaled with the magnitude of placebo analgesia). ***I***, The DMPFC (magenta) was the only region that showed overlapping activation between pure stimulus expectancies and pure treatment expectancies. ***II***, In addition to testing for overlap, we examined whether any regions responded similarly to expectations for high pain relative to low pain, whether driven by stimulus expectancies or treatment expectancies. Individuals with larger placebo responses showed stronger rACC activation with expectations for reduced pain with both types of expectancy. There were no main effects, and no other regions survived correction (see Extended Data Fig. 7-1 for complete results). ***B***, Differences between stimulus and treatment expectancy. We tested whether stimulus and treatment expectancies are associated with distinct brain mechanisms by evaluating responses on trials in which both factors were crossed as well as on pure trials. In both cases, only associations with individual differences in placebo analgesia survived multiple-comparison correction. ***I***, Consistent with behavioral interactions on crossed trials, individuals with larger placebo responses showed weaker cue effects on brain activation during placebo blocks, relative to control blocks, in the bilateral hippocampus, right insula, right dorsomedial prefrontal cortex, and other regions (warm regions; Table 6). We also observed stronger cue effects on pons activation (blue) and other regions on control blocks, relative to placebo blocks (Table 6). Responses in the right anterior insula (bottom) were driven by negative associations on placebo blocks and positive associations on control blocks. Uncorrected results are reported in Extended Data Fig. 7-2. ***II***, We also tested interactions by directly contrasting pure cue effects with pure placebo effects. The DLPFC showed interactions such that individuals with larger placebo responses showed stronger DLPFC activation in response to high pain expectations based on cues (HM > LM), but showed stronger DLPFC activation with expectations for pain relief based on treatment (placebo > control). Interactions were also observed in the TPJ, operculum, and pons (see Table 7 for complete results; uncorrected results are reported in Extended Data Fig. 7-3).

We first compared pure stimulus expectancy effects [i.e. (HM > LM) prior to the treatment manipulation] with pure treatment expectancy effects (i.e. [Control > Placebo] on uncued medium heat trials) to test whether these depended on shared or distinct mechanisms. When we searched for spatial overlap between main effects of stimulus expectancy and associations between treatment expectancy and placebo analgesia, the DMPFC extending to preSMA was the only region that was affected by both cues and treatment (Fig. 7*A*). In both cases, DMPFC responses were reduced with low pain expectations (i.e. following low cues or during placebo administration). To further investigate common effects, we evaluated whether any brain region was modulated by both stimulus expectancy and treatment expectancy during medium heat trials by contrasting high pain expectancy (high cues and uncued control blocks) with low pain expectancy (low cues and uncued placebo blocks); individual differences in placebo analgesia were treated as a potential moderator. Whole-brain correction revealed that the magnitude of common activation varied as a function of placebo analgesia in the rostral anterior cingulate (Fig. 7*A*), such that individuals with a larger placebo response showed stronger rACC activation with expectations for low pain, whether based on cues or placebo. Consistent with other analyses, there were no effects on NPS or SIIPS and main effects regardless of placebo analgesia did not survive correction; complete uncorrected results are reported in Extended Data.

10.1523/JNEUROSCI.0050-25.2025.f7-1 Figure 7-1 Associations between placebo analgesia and differences between high pain expectancy and low pain expectancy. Download Figure 7-1, DOCX file.

10.1523/JNEUROSCI.0050-25.2025.f7-2 Figure 7-2 Interactions on fully-crossed trials: Uncorrected results and correction within regions of interest. Download Figure 7-2, DOCX file.

10.1523/JNEUROSCI.0050-25.2025.f7-3 Figure 7-3 Associations between placebo analgesia and differences between stimulus and treatment expectancy effects: Uncorrected results. Download Figure 7-3, DOCX file.

Next, we tested for neural interactions between stimulus expectancy and treatment expectancy on crossed trials by asking whether cue effects differ under control or placebo [(cHM − cLM) > (pLM − pHM)]. Only moderation by individual differences in placebo analgesia survived multiple-comparison correction. We did not observe associations in NPS or SIIPS, nor did any regions survive corrections within a priori pain and placebo networks. Correction within nociceptive regions revealed positive associations between individual differences in placebo analgesia and stimulus × treatment expectancy interactions in the left parahippocampus and right superior temporal gyrus. Whole-brain FDR correction revealed positive associations in additional regions, including the bilateral hippocampus, right ventral anterior insula, left DLPFC, and other regions (Fig. 7*B*; Table 6). Most positive regions showed negative correlations between placebo analgesia and cue effects on placebo blocks, such that individuals with larger placebo responses showed weaker cue effects on brain responses during placebo administration, consistent with behavioral interactions (Table 6); some regions, such as the right anterior insula, were driven by both negative associations on placebo blocks and positive associations on control blocks (Fig. 7*Bi*). Negative associations were evident in the pons, right dorsal anterior insula, and the cerebellum driven by positive associations between placebo analgesia and cue effects on control blocks (Fig. 7*B*; Table 6).

**Table 6.** Associations between placebo analgesia and interactions on fully crossed trials^a^

Contrast	Anatomical label	*x*	*y*	*z*	# of voxels	Volume (mm^3^)	Max stat	Association between PA and (HM–LM) during control blocks^b^	Association between PA and (HM–LM) during placebo blocks^b^	Association between PA and (HM–LM) × (control–placebo)^b^
Positive associations	R Cerebellum Crus 1	46	−62	−38	6	162	10.72	*n.s.*	*n.s.*	*n.s.*
	R Cerebellum Crus 2	22	−80	−34	6	162	10.46	2.593*	−2.866**	4.242***
	L Superior Temporal Gyrus/Anterior Insula	−50	4	−14	100	2,700	12.88	2.476*	−3.078**	7.911***
	R Lingual Gyrus (Area hOc2 [V2])	10	−76	−2	467	12,609	11.27	4.865***	−2.755**	7.562***
	R Superior Temporal Gyrus/Anterior insula	46	2	−14	47	1,269	10.3	*n.s.*	−2.865**	4.073***
	L Hippocampus (DG)^c^	−34	−28	−14	21	567	11.63	3.898***	*n.s.*	5.536***
	R Fusiform/Posterior Hippocampus	38	−40	−8	82	2,214	13.29	4.770***	−3.538***	6.852***
	R Hippocampus	32	−28	−4	13	351	12.47	2.736*	−2.256*	2.816*
	L Middle Orbital Gyrus (Area Fp1/VMPFC)	−22	58	−10	14	378	11.42	*n.s.*	*n.s.*	−2.558*
	L Calcarine Gyrus (Area hOc1 [V1])	−10	−88	2	70	1,890	10.72	2.278*	−2.419*	3.684***
	R Superior Temporal Gyrus (Area TE 3)^c^	64	−10	−2	10	270	10.45	*n.s.*	*n.s.*	4.765***
	R Middle Occipital Gyrus (Area hOc4la)	50	−76	4	18	486	10.52	2.026*	*n.s.*	2.485*
	R IFG p. Triangularis	40	26	4	45	1,215	10.36	3.556***	−3.672***	6.093***
	L Precuneus	−22	−50	8	20	540	11.89	2.167*	*n.s.*	4.586***
	L SupraMarginal Gyrus	−50	−40	32	9	243	11.74	2.263*	−3.429**	4.076***
	R Superior Frontal Gyrus	22	20	52	47	1,269	11.23	*n.s.*	−3.258**	4.230***
	L Precentral Gyrus	−38	2	62	26	702	12.69	2.675*	*n.s.*	7.049***
	RPrecentral Gyrus	28	−26	62	15	405	10.61	3.309**	−3.047**	4.701***
Negative associations	R Cerebellum VIII	20	−52	−56	16	432	10.78	−2.148*	5.359***	−6.688***
	L Cerebellum VIII	−20	−58	−58	13	351	10.3	*n.s.*	5.968***	−4.756***
	R Inferior Temporal Gyrus	40	−8	−44	10	270	11.95	−3.316**	*n.s.*	−3.664***
	L Pons	−8	−20	−26	16	432	11.04	−2.164*	2.478*	−4.778***
	R Inferior Temporal Gyrus	52	−50	−22	9	243	12.39	*n.s.*	*n.s.*	−4.139***
	R Middle Orbital Gyrus	44	56	−10	4	108	11.49	*n.s.*	*n.s.*	−6.635***
	R Middle Frontal Gyrus	50	50	4	6	162	11.26	*n.s.*	2.312*	−2.448*
	R Insula Lobe (Area OP3 [VS])	38	−4	10	27	729	11.52	−2.647*	3.264**	*n.s.*
	R Middle Frontal Gyrus	20	58	28	18	486	11.08	*n.s.*	*n.s.*	−3.153**

a This table presents whole-brain FDR-corrected results of robust regression evaluating associations between the magnitude of placebo analgesia (controlling for the counterbalanced order) and interactions of treatment expectancy (control–placebo) and stimulus expectancy (high cue–low cue) on heat-evoked activation on medium trials that were crossed with both types of expectancy. No regions survived correction within a priori regions involved in pain and placebo. See Extended Data Figure 7-2 for uncorrected results and main effects controlling for individual differences in placebo analgesia.

b Columns report *t* statistics and *p* values denoting the significance of associations between placebo analgesia (PA) and stimulus expectancy effects (high cue–low cue) on brain activation based on robust regression. We separately report associations during control runs, placebo runs, or the difference between control and placebo to understand the source of the observed interactions. ***p < 0.001; **p < 0.01; *p < 0.05; *n.s.*, *p* > 0.05.

c Associations in these clusters survived small volumes correction within nociceptive networks in this sample.

Finally, we contrasted pure stimulus expectancy effects with pure treatment expectancy effects to isolate brain regions where neural responses differed based on whether expectations were driven by cues or treatment. Consistent with all other analyses of placebo effects, only moderation survived whole-brain correction, and there were no interactions on the NPS (*p* > 0.6). Whole-brain correction revealed positive associations in the right DLPFC (middle frontal gyrus), left inferior and middle temporal gyrus, and the right inferior parietal lobule (Fig. 7*B*; Table 7). In each of these regions, individuals with a larger placebo response showed stronger positive activation with cue-based expectations for higher pain (i.e., high cues, relative to low cues) and also showed stronger activation with treatment-induced expectations for lower pain (i.e., placebo, relative to control), as illustrated for DLPFC in Figure 7*Bii*. Negative associations were only observed in the pons (Fig. 7*Bii*; Table 7); this region showed negative associations between placebo analgesia and cue effects on activation (i.e., individuals with larger placebo responses showed larger increases in pons activation with low pain cues, relative to high pain cues), but no association between placebo response and placebo effects on activation during treatment blocks (Table 7).

**Table 7.** Associations between placebo analgesia and differences between stimulus and treatment expectancy effects^a^

Contrast	Anatomical Label	*x*	*y*	*z*	# of voxels	Volume (mm^3^)	Max stat	Association between PA and pure stimulus expectancy (high cue–low cue)^b^	Association between PA and pure treatment expectancy (control–placebo)^b^	Association between PA and (stimulus expectancy-treatment expectancy)^b^
Positive association	L Inferior Temporal Gyrus	−64	−40	−16	73	1,971	13.84	4.410***	−4.095***	4.957***
	L Middle Temporal Gyrus	−46	−56	20	43	1,161	12.11	*n.s.*	−3.388**	3.673***
	R Angular Gyrus (Area PGp (IPL))	52	−68	32	13	351	14.76	2.877**	−3.093**	4.480***
	R Middle Frontal Gyrus (DLPFC)	44	16	52	73	1,971	15.62	4.474***	−2.597*	4.574***
Negative association	L Pons	−8	−20	−32	4	108	12.12	−2.320*	*n.s.*	−3.767***

a This table presents whole-brain FDR–corrected results of robust regression evaluating associations between the magnitude of placebo analgesia (controlling for the counterbalanced order) and differences between pure treatment expectancy [(control–placebo] on uncued medium trials] and pure stimulus expectancy [(high cue–low cue) prior to treatment] on heat-evoked activation on medium trials. No regions survived correction within nociceptive regions or a priori regions involved in pain and placebo. See Extended Data Figure 7-3 for uncorrected results.

b Columns report *t* statistics and *p* values denoting the significance of associations between placebo analgesia (PA) and expectancy effects on brain activation based on robust regression. We separately report associations with pure stimulus expectancy effects [(high cue–low cue) prior to treatment), pure treatment expectancy effects [(control–placebo) on uncued trials], or the contrast between pure stimulus expectancy and pure treatment expectancy to understand the source of the observed interactions. ***p < 0.001; **p < 0.01; *p < 0.05; *n.s.*, *p* > 0.05.

### Additional preregistered analyses

#### Effects of time

**Figure 8.**
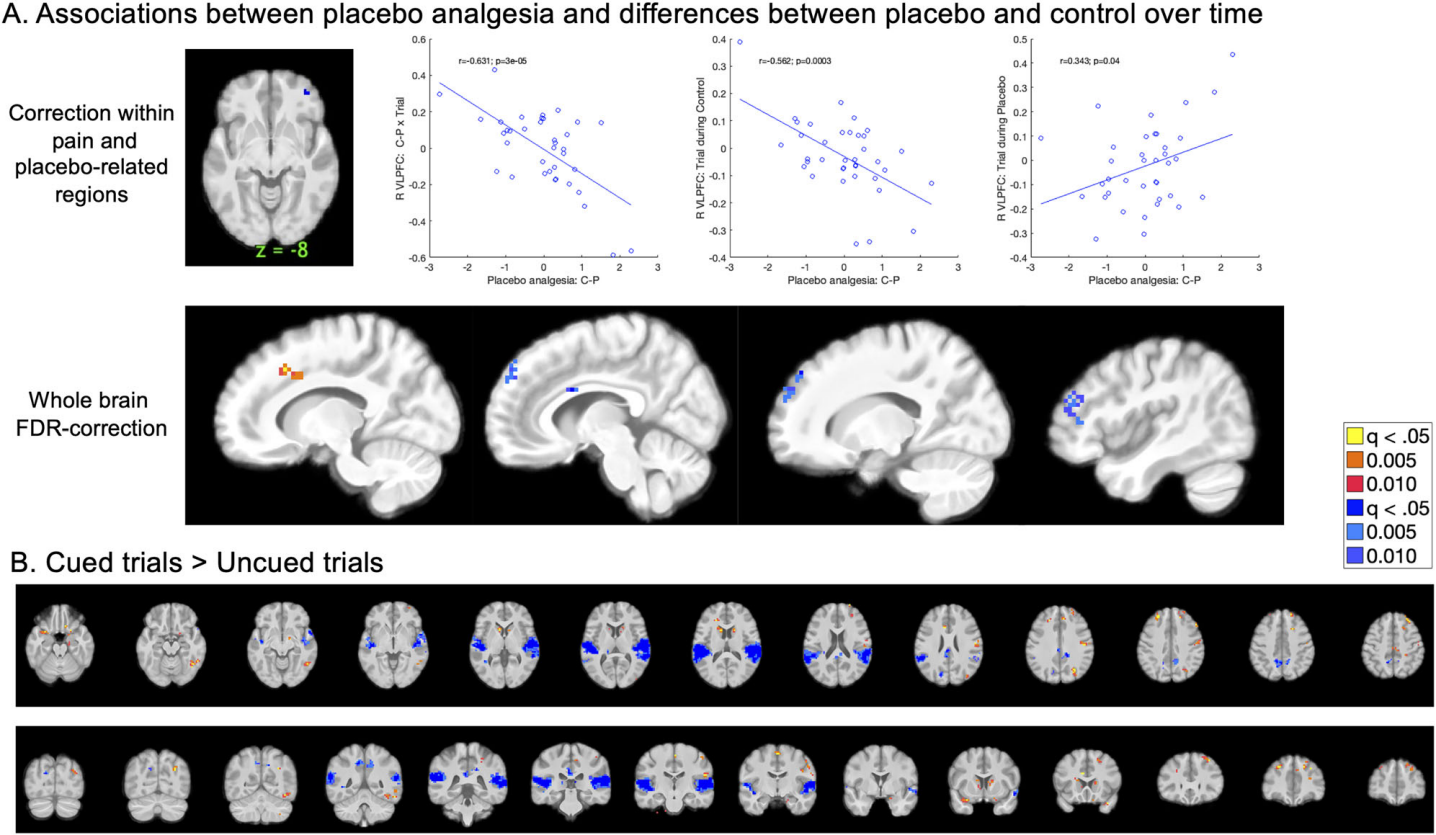
Additional preregistered analyses. ***A***, We hypothesized that regions involved in placebo would show decreases over time during control blocks but not placebo blocks. Here, we present robust results examining interactions between treatment expectancy (control > placebo) and time. Treatment × time interactions varied as a function of individual differences in placebo analgesia in the right VLPFC based on correction within regions involved in placebo analgesia (top row). Extracting responses from this region revealed that individuals who showed stronger placebo analgesia did show VLPFC reductions over time during control blocks (middle) as hypothesized and also showed increases in VLPFC activation over time during placebo blocks (right). Whole-brain correction revealed additional associations between placebo analgesia and differences over time in the left dACC, right dACC, right DMPFC, and right DLPFC. See Extended Data Figure 8-1 for associations with placebo analgesia as a function of treatment and time in these regions. Complete results are reported in Extended Data Figure 8-2. ***B***, There were robust differences in activation as a function of whether cues were presented prior to stimulation. Here we present FDR-corrected results comparing responses on medium trials preceded by cues relative to uncued medium trials. Complete results including uncorrected results are reported in Extended Data Figure 8-3.

We hypothesized that long-lasting opioid modulation might lead to changes in activation in placebo-related brain responses over time, such that there would be reductions in heat-evoked activation over time during control administration in regions involved in placebo analgesia, including DLPFC, OFC, PAG, rACC, ventral striatum, and caudate. We thus compared effects of time between placebo blocks and control blocks to test treatment expectancy × trial interactions. For each condition, a regressor was created to capture linear changes across trials during the four blocks corresponding to that condition (i.e., trials during either Runs 4–7 or 8–11, depending on the counterbalanced order), and we used robust regression to examine main effects and associations with placebo analgesia, when controlling for the treatment order. Only associations with placebo analgesia survived correction. When we compared effects of trial between placebo and control conditions within a priori regions involved in pain and placebo, we observed negative associations between changes over time and placebo analgesia in the right VLPFC. Extracting from this region revealed that individuals with larger placebo responses showed decreases over time during control blocks, as hypothesized, as well as larger increases over time during placebo blocks (Fig. 8*A*). Whole-brain FDR correction additionally revealed positive associations in the left dACC and negative associations in the right dACC, right DMPFC, and right DLPFC (Fig. 8*A*; Extended Data Fig. 8-1). Extracting from individual regions revealed that these interactions were primarily driven by associations between placebo analgesia and changes over time during placebo blocks, except for in the dACC which was influenced during both placebo and control blocks (Extended Data Fig. 8-2). No effects survived correction within nociceptive regions. Complete results, included uncorrected results, are reported in Extended Data Figure 8-3.

10.1523/JNEUROSCI.0050-25.2025.f8-1 Figure 8-1 *Relationship between placebo analgesia and changes over time.* Treatment x Time interactions varied as a function of individual differences in placebo analgesia in the dorsal anterior cingulate (top row), right DLPFC (second row), right DLPFC (third row), and right DMPFC (bottom row). Graphs illustrate associations between placebo analgesia and a) the difference in changes over time between placebo and control blocks (left), b) changes over time during control blocks (middle); and c) changes over time during placebo blocks (right). Download Figure 8-1, TIF file.

10.1523/JNEUROSCI.0050-25.2025.f8-2 Figure 8-2 Treatment expectancy effects on changes over time. Download Figure 8-2, DOCX file.

10.1523/JNEUROSCI.0050-25.2025.f8-3 Figure 8-3 Impact of cue presentation on brain response. Download Figure 8-3, DOCX file.

#### PAG–rACC background connectivity

We preregistered the hypothesis that communication between the PAG and rACC would be higher under placebo than control. We were specifically interested in background connectivity, i.e., correlations in residual, rather than task-based, activation. To evaluate this, we extracted timeseries data for each participant from masks of the PAG and rACC. The rACC mask was generated from rACC coordinates that showed significant opioid changes in connectivity with PAG under placebo analgesia in Eippert et al. (2009a), while the PAG mask was based on the Harvard Ascending Arousal Network Atlas (Edlow et al., 2012). For each timeseries, we regressed out the overall design matrix (cue, heat, scale/rating, TRs for scanner stabilization, and last 2 TRs for baseline measure) and nuisance parameters (motion, spikes, and average activation in white matter and ventricles), which provided estimates of the residual timeseries for each region and each participant. We then used linear mixed models to evaluate whether rACC–PAG background connectivity varied as a function of (1) treatment expectancy (placebo vs control), (2) individual differences in placebo analgesia, or (3) interactions between these effects. Our analysis of background connectivity between the PAG and rACC revealed a negative relationship between rACC and PAG residual activation (*B* = −0.05; *p* = 0.001). However, in contrast to our hypotheses, rACC–PAG connectivity did not differ as a function of treatment expectancy, placebo analgesia, or their interaction (all *p*'s > 0.5).

#### Impact of expectancy cues on brain activation

Consistent with our preregistration, we compared cued medium heat trials (across all blocks) with uncued trials (collapsing across placebo and control blocks) to test the hypothesis that stimulus expectancies would engage regions involved in prediction error. Cued trials were rated as slightly more painful than uncued trials (*B* = 0.40; *p* < 0.001). Consistent with our hypothesis, whole-brain FDR correction revealed positive effects (cued > uncued) in the right amygdala, bilateral caudate, and right mOFC/sgACC, regions that are involved in value-based learning (Fig. 8*B*). We also observed positive effects in bilateral DMPFC and negative effects (uncued > cued) in the bilateral auditory cortex extending into SII, the posterior cingulate, and the cuneus. Complete results are reported in Extended Data Figure 8-3.

#### Order effects

The robust regressions reported above report results controlling for the counterbalanced order (i.e., whether participants experienced testing on the placebo or control site first or underwent trials with or without cues first). In separate analyses, we evaluated whether the order impacted subjective pain. We separately analyzed responses on cued and uncued trials.

Pain ratings on cued trials were influenced by both types of counterbalancing. We observed a significant treatment expectancy × treatment order interaction (*B* = −0.31; CI = [−0.60, −0.02]; *p* = 0.039), driven by a main effect of treatment expectancy (*B* = −0.56; CI = [−0.89, −0.23]; *p* < 0.001) for participants who experienced testing on placebo sites first, but not those who rated trials on control sites first (*p* > 0.8). We also observed a significant stimulus expectancy × heat × cue order interaction (*B* = −0.29; CI = [−0.55, −0.03]; *p* = 0.03) and a significant heat × treatment expectancy × cue order interaction (*B* = −0.28; CI = [−0.54, −0.02]; *p* = 0.037). Post hoc tests revealed that there was a main effect of treatment expectancy for participants who first underwent testing without cues (*B* = −0.43; CI = [−0.82, −0.04]; *p* = 0.031), whereas there was no main effect of treatment expectancy for participants who first underwent cued trials (*p* > 0.6). Instead, participants who experienced cued trials first experienced interactions between heat and treatment expectancy (*B* = 0.63; CI = [0.25, 1.01]; *p* < 0.001), between stimulus expectancy and treatment expectancy (*B* = −0.75; CI = [−1.28, −0.22]; *p* = 0.005), and between heat, treatment, and stimulus expectancy (*B* = 0.78; CI = [0.03, 1.53]; *p* = 0.041). Evaluating tests separately within each heat level in participants who were tested with cues first revealed that there were no effects of treatment expectancy on low or high heat trials (all *p*'s > 0.3), but on medium trials there was a main effect of stimulus expectancy (*B* = 1.21; CI = [0.95, 1.47]; *p* < 0.001), as well as a marginal main effect of treatment expectancy (*B* = −0.28; CI = [−0.58, 0.05]; *p* = 0.097) and a marginal treatment expectancy × stimulus expectancy interaction (*B* = −0.4; CI = [−0.81, 0.00]; *p* = 0.053).

Pain ratings on uncued trials were influenced by the treatment order, as indicated by a heat × treatment expectancy × treatment order interaction (*B* = −0.29; CI = [−0.54, −0.05]; *p* = 0.017). There were no effects of the cue order or other interactions on uncued trials. Post hoc tests revealed no effects of treatment expectancy on uncued trials in participants who were tested on placebo sites first (all *p*'s > 0.3) but a significant heat × treatment expectancy interaction in participants who were tested on control sites first (*B* = 0.44; CI = [0.08, 0.81]; *p* = 0.017), driven by participants reporting more pain with placebo on high heat trials (*B* = 0.57; CI = [0.01, 1.13]; *p* = 0.047) but no effect of treatment expectancy on uncued low or medium heat trials (all *p*'s > 0.2). Thus, although the order influenced responses overall, there was no effect of the order on critical medium heat trials, whether we focused on cued or uncued responses.

## Discussion

We used a quasi-factorial design to measure how pain is modulated by cue-based expectations about noxious stimulus intensity and treatment-based expectations about pain relief, i.e., placebo analgesia. Pain was strongly influenced by stimulus expectancies in nearly every participant, whereas treatment expectancy effects varied across individuals, and there was no placebo effect across participants. Crossing stimulus expectancies with treatment expectancies revealed both dissociations and interactions. Here, we discuss implications of these findings.

### Behavioral and neural interactions

Pain was strongly influenced by predictive cues, but cue effects were reduced during placebo blocks. This interaction indicates that treatment expectancies can reduce the impact of stimulus expectancies, even when placebo analgesia varies across individuals. We observed similar interactions in brain regions, such that individuals with larger placebo responses exhibited weaker cue effects on responses in the pons, bilateral hippocampus, and bilateral anterior insula under placebo, relative to control. We also observed interactions when we directly compared pure stimulus expectancy effects prior to treatment with pure treatment expectancy effects on uncued trials. We again observed negative interactions in the pons, as well as the right DLPFC, left inferior temporal gyrus, and left temporoparietal junction. Preclinical work (Chen et al., 2024) indicates that the pons mediates conditioned placebo effects via projections from the rACC and opioidergic projections to the cerebellum. During baseline and placebo blocks, individuals with larger placebo responses showed stronger pons activation in response to high pain cues, relative to low pain cues, replicating prior work (Atlas et al., 2010). However, consistent with neural interactions, we observed the opposite pattern on control blocks (greater pons activation with low pain cues), and there was no association between placebo analgesia and treatment expectancy effects on pons activation. This is the first demonstration that this region (among others) plays a different role in pain depending on whether expectations are driven by cues or placebos. Future preclinical studies should compare cue-based expectations (e.g., pavlovian learning) with context-based expectations (e.g., conditioned place aversion) to determine whether similar dissociations underlie pain relief learning.

Building on interactions, we observed dissociations between placebo effects and cue effects when we measured each separately. Cues modulated NPS expression but not SIIPS expression, consistent with prior work (Woo et al., 2017), and we observed cue-based modulation of the right dorsal posterior insula, which might be specific to pain (Segerdahl et al., 2015). Placebo administration did not impact the NPS, consistent with preregistered hypotheses and prior studies (Zunhammer et al., 2018; Botvinik-Nezer et al., 2024). These findings suggest that cues modulate nociceptive pain while placebos act on other circuits. Surprisingly, although SIIPS expression was influenced by changes in heat intensity, neither cues nor placebo modulated SIIPS, and SIIPS did not predict pain when controlling for expectancy, contrasting prior work (Woo et al., 2017; Botvinik-Nezer et al., 2024). In exploratory analyses (https://osf.io/tr3z8/), we evaluated correlations between NPS and SIIPS expression across conditions. There were no correlations when we measured pure stimulus expectancy effects or pure treatment expectancy effects, whereas pattern expression was correlated for high versus low heat, when stimulus expectancies were crossed with treatment expectancies and when medium heat was preceded by low pain cues. Future research should further investigate dissociations between signature patterns.

### Common effects on modulatory regions

Prior work on expectancy-based pain modulation has implicated the rACC, DLPFC, and VMPFC, regions involved in cognitive control and emotion regulation. All three regions were impacted by both types of expectancy in this study. Individuals with larger placebo responses showed increased rACC activation with low pain expectancy (low cues or placebo). As mentioned above, the rACC is linked with opioid analgesia and descending pain modulation through connectivity with the PAG, pons, and cerebellum (Bingel et al., 2006; Eippert et al., 2009a; Chen et al., 2024). Although placebo analgesia was associated with cerebellum activation and the PAG mediated dynamic placebo effects on pain, placebo administration did not affect rACC–PAG background connectivity, in contrast to preregistered hypotheses.

Extending prior work on cue effects and placebo effects (Wager et al., 2004; Atlas et al., 2010), the DLPFC was sensitive to both cue-based stimulus expectancies and treatment expectancies. However, direct comparisons revealed interactions, such that DLPFC activation was associated with pain relief based on treatment expectancy and pain exacerbation based on cue expectancy. Given the DLPFC’s role in executive function, future work should test whether high cues and placebo administration engage greater cognitive control than low cues and control treatments, respectively. Finally, the VMPFC was implicated in our mediation analysis of placebo effects and was sensitive to both treatment and stimulus expectancies in uncorrected analyses. Together, these findings indicate that the rACC, DLPFC, and VMPFC are fundamental for expectancy-based pain modulation, whether expectations are driven by cues or treatment context.

### Mechanisms for dissociations

Our findings suggest that treatment expectancies modulate value and decision-making circuits, while stimulus expectancies impact nociceptive networks. We have hypothesized that these depend on separate neuromodulatory mechanisms (Atlas and Wager, 2012; Atlas, 2021), with placebos engaging tonic opioidergic descending modulation and cues relying on phasic dopaminergic prediction errors. Pharmacological antagonists reveal that placebo analgesia is not dependent on dopamine activity (Wrobel et al., 2014; Kunkel et al., 2024) but does depend on endogenous µ-opioid receptor activation (Levine et al., 1978; Eippert et al., 2009a). Associations between behavioral placebo effects and placebo-induced changes in rACC activation might be consistent with this, as might findings that placebo analgesia was correlated with changes over time in VLPFC and dACC during control blocks, which could reflect opioid effects that persist beyond the placebo manipulation and test phase. However, striatal responses also mediated placebo effects, and we did not observe increased rACC–PAG connectivity under placebo, which has been shown to be opioidergic (Eippert et al., 2009a). Further pharmacological investigations should isolate the involvement of dopamine and endogenous opioid activity in both processes. In addition, because we hypothesized that placebos could engage long-lasting pain modulation, we always tested pure cue-based expectancy effects before the treatment expectancy manipulation. Future studies should counterbalance the order to test potential carryover effects.

We note that behavioral interactions are inconsistent with complete dissociations. Interactions also contrast with our findings of additivity between stimulus expectancies, treatment expectancies, and opioid analgesic treatment during a balanced placebo design (Atlas et al., 2014b). However, the current study was larger than our previous study and incorporated analgesic conditioning, whereas balanced placebo designs rely purely on verbal information to induce treatment expectancy. Future studies should compare stimulus expectancies, treatment expectancies, and interactions in different experimental contexts.

### Outstanding questions and recommendations

Our study represents the first to directly compare stimulus and treatment expectancies on pain and pain-related responses. Our findings highlight the need for greater precision in pain research: many putative studies of placebo analgesia combine both types of manipulations (e.g., using cues to signal placebo administration) or only manipulate stimulus expectancies. Several questions and limitations can be addressed in future work. First, our use of a within-subject quasi-factorial design limited the number of trials per condition, likely reducing power. We included as many trials as we could safely administer, which also may have limited generalizability, as participants needed six temperature-responsive skin sites. Second, our study did not elicit a robust placebo effect across participants. One contributing factor to our weak placebo effects might be that we tested placebo effects across temperatures, whereas most studies use a single level of heat during the test phase (Price et al., 1999; Wager et al., 2004; Eippert et al., 2009a,b). When temperature varies, top–down expectations might compete with bottom–up information and diminish the strength of treatment expectations for some participants (Case et al., 2019). Another contributing factor could be that we used weaker conditioning to establish treatment expectations than stimulus expectations. Stronger conditioning might have elicited more robust placebo responses. A previous between-group study used matched conditioning to induce stimulus or treatment expectancies (Schenk et al., 2017) and found that stimulus expectancies induced larger striatal prediction errors and weaker expectancy effects during test. They concluded that placebo hyperalgesia suppresses error-driven learning. If this were true, we would expect that our use of larger differences to condition cue-based stimulus expectancies would lead to faster learning and “weaker” stimulus expectancy effects than treatment expectancy effects. Future studies should use the same conditioning levels in a within-subject design to differentiate between these possibilities while testing for interactions. Schenk et al. (2017) also used identical cues to signal treatment or stimulus expectancy, while we used different cues with different timescales to facilitate testing for interactions. Future work should use the same modalities to signal both types of expectancy and to compare interleaved versus blocked training.

Finally, our attempts to identify brain regions that respond to both stimulus and treatment expectancy rely on spatial conjunction analyses and statistical contrasts. Although only the DMPFC, rACC, and the VMPFC showed common activation, spatial overlap is not sufficient to determine that there are no differences in activation, as there may be distinguishing patterns within regions. Future studies that incorporate more trials for each condition should use multivoxel pattern analyses to see whether the patterns of expectancy effects within a given region can discriminate between types of expectancy.

## Conclusions

By crossing predictive cues with placebo administration, we were able to compare the effects of stimulus and treatment expectancies on pain and pain-related brain responses. We observed interactions in both outcomes, such that cue effects on pain and pain-related responses in several regions were reduced under placebo. We also observed dissociations in expectancy effects on brain responses, such that cues impacted nociceptive pain networks while treatments impacted evaluative and opioidergic regions. These findings emphasize the need to treat stimulus and treatment expectancies as separate processes, rather than lumping all expectancy effects together under the umbrella of placebo analgesia. Our findings also have clinical implications: When patients expect an analgesic treatment (treatment expectancy), information about an otherwise painful procedure is likely to matter less. However, even during analgesic treatments, stimulus expectancies modulate pain with large effect sizes and directly impact nociceptive processing. Thus, medical providers should consider the impact not only of the analgesic treatments that they provide but also how they describe painful procedures themselves. Pain relief and patient well-being can be maximized by addressing both types of expectations.

## Data Availability

Code and behavioral data are available at https://osf.io/tr3z8/. FMRI results are available on Neurovault at https://identifiers.org/neurovault.collection:20308.
