# Supplementary figures and images for: Isolating Brain Mechanisms of Expectancy Effects on Pain: Cue-Based Stimulus Expectancies versus Placebo-Based Treatment Expectancies
Source: J Neurosci. 2025 Jul 28;45(34):e0050252025. doi: 10.1523/JNEUROSCI.0050-25.2025 (PMC12369932; doi:10.1523/JNEUROSCI.0050-25.2025)

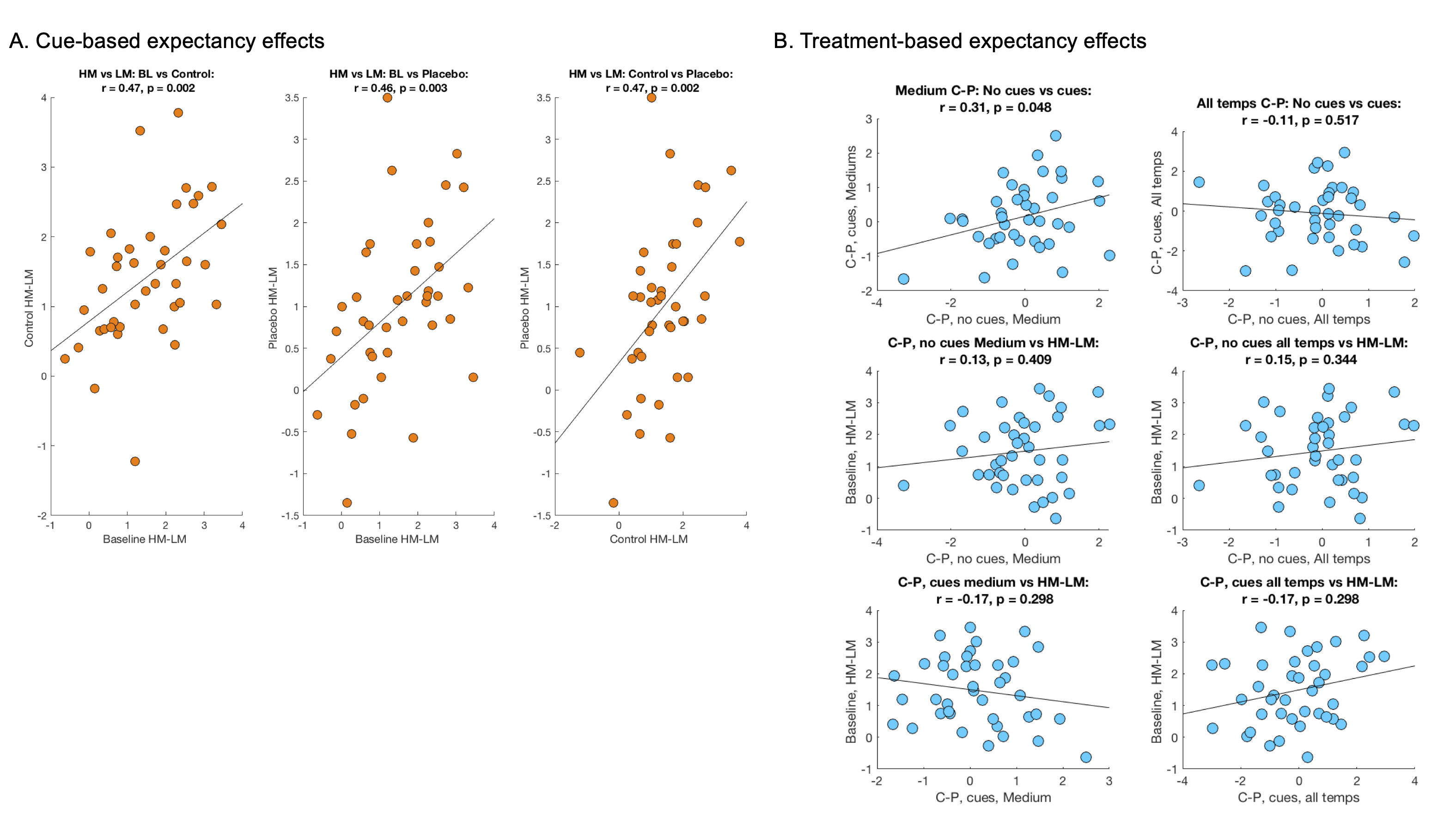

Supplement: Figure 2-1 — Relationships between stimulus and treatment expectancy effects. In exploratory analyses, we evaluated whether there were correlations between cue effects and treatment effects. A) Cue-based expectancy effects. Cue-based stimulus expectancy effects were consistent across individuals, as evident by correlations between pre-treatment (“Baseline”) cue effects (HM > LM) and cue effects on pain during control treatment (left) and placebo treatment (middle), as well as correlations in cue effects between placebo and control blocks (right). B) Treatment based expectancy effects. Treatment-based expectancy effects on pain (Control > Placebo) were correlated on cued and uncued medium heat trials (top left), but there were no associations across temperatures (top right), or were there any correlations with between treatment-based expectancy effects and cue-based stimulus expectancy effects, regardless of cues or temperatures (middle and bottom rows). Download Figure 2-1, TIF file. [file jneuro-45-e0050252025-s001.tif]

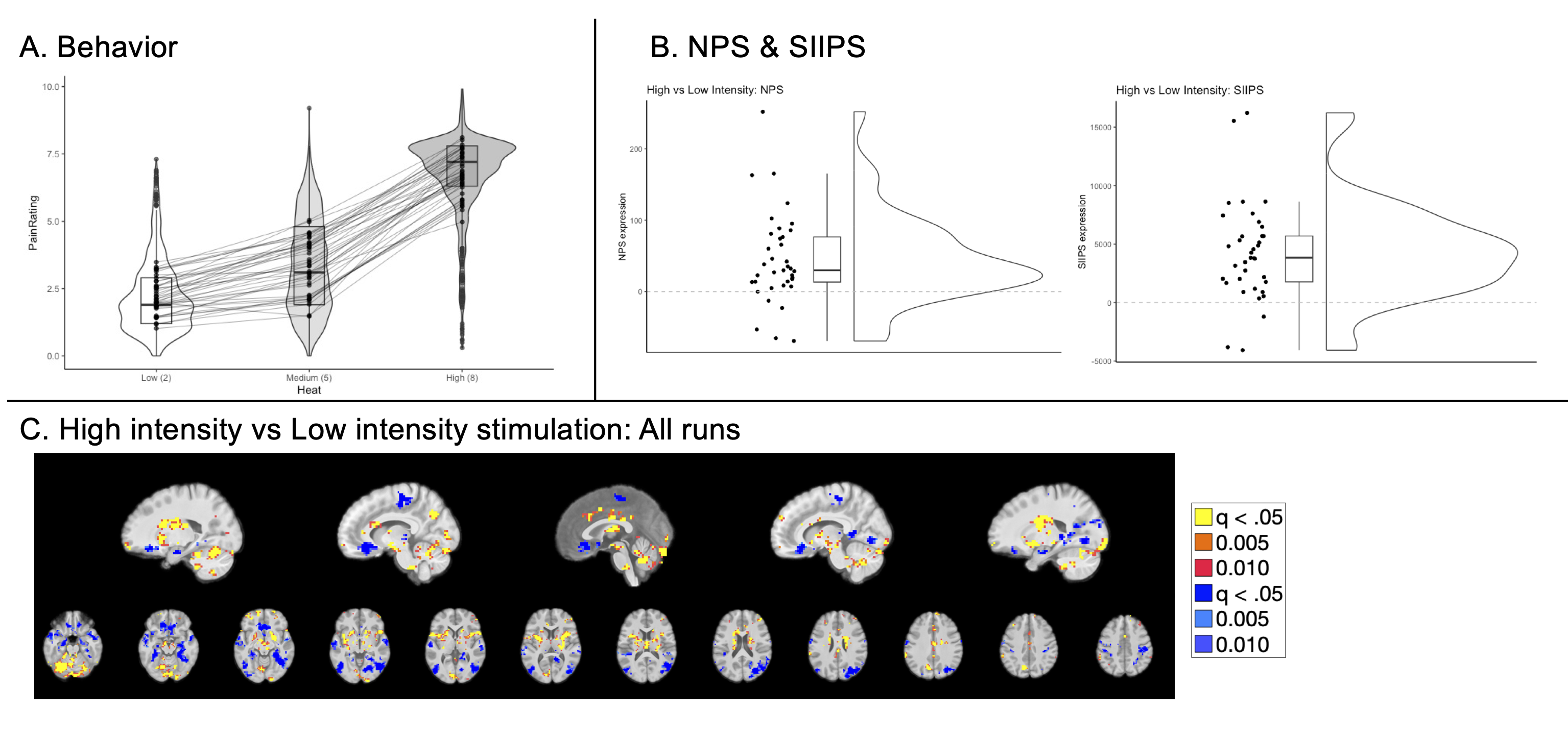

Supplement: Figure 3-2 — Stimulus intensity effects across all runs. Our main manuscript compares low and high heat stimulation prior to the treatment manipulation to isolate nociceptive networks. Results were similar when we measured effects across all runs. A) All subjects reported increases in pain as a function of stimulus intensity. B) There was a significant effect of stimulus intensity on NPS and SIIPS expression across the entire task. C) Regions that showed differences between high and low intensity stimulation were similar whether we included all runs in analyses (see Extended Data Figure 3-3 for complete results) or measured responses only prior to the treatment manipulation, as reported in the main manuscript. Download Figure 3-2, TIF file. [file jneuro-45-e0050252025-s003.tif]

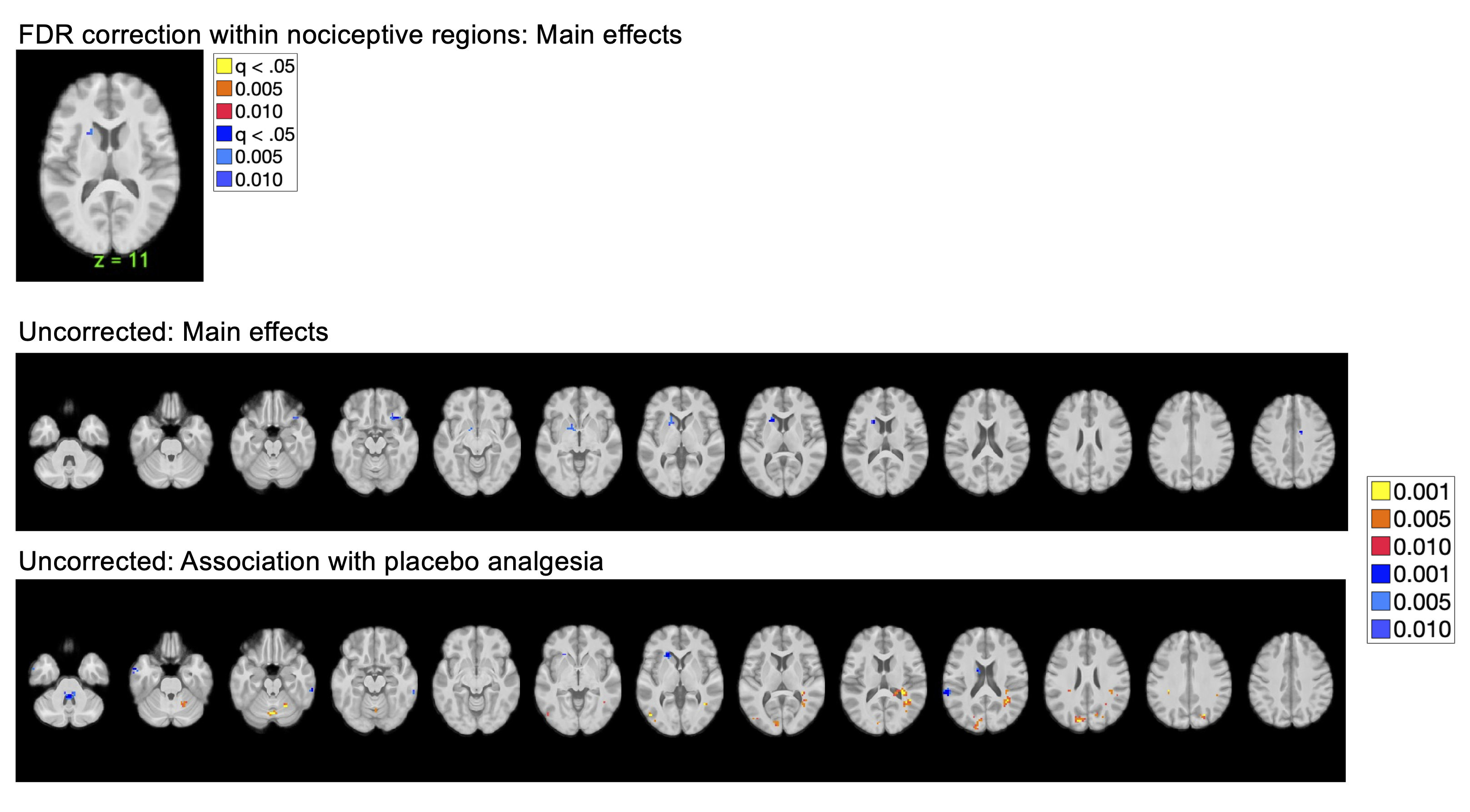

Supplement: Figure 5-2 — Placebo effects across temperatures. Our main analyses focus on placebo effects on uncued medium heat trials, to control for changes in temperature. Here, we present results on uncued trials across all temperatures. Top: The only effect that survived multiple comparisons correction was a main effect of Treatment Expectancy in the left caudate, driven by higher activation on placebo blocks, relative to control blocks. Uncorrected main effects (middle) and associations (bottom) are reported in Extended Data Figure 5-3. Download Figure 5-2, TIF file. [file jneuro-45-e0050252025-s008.tif]

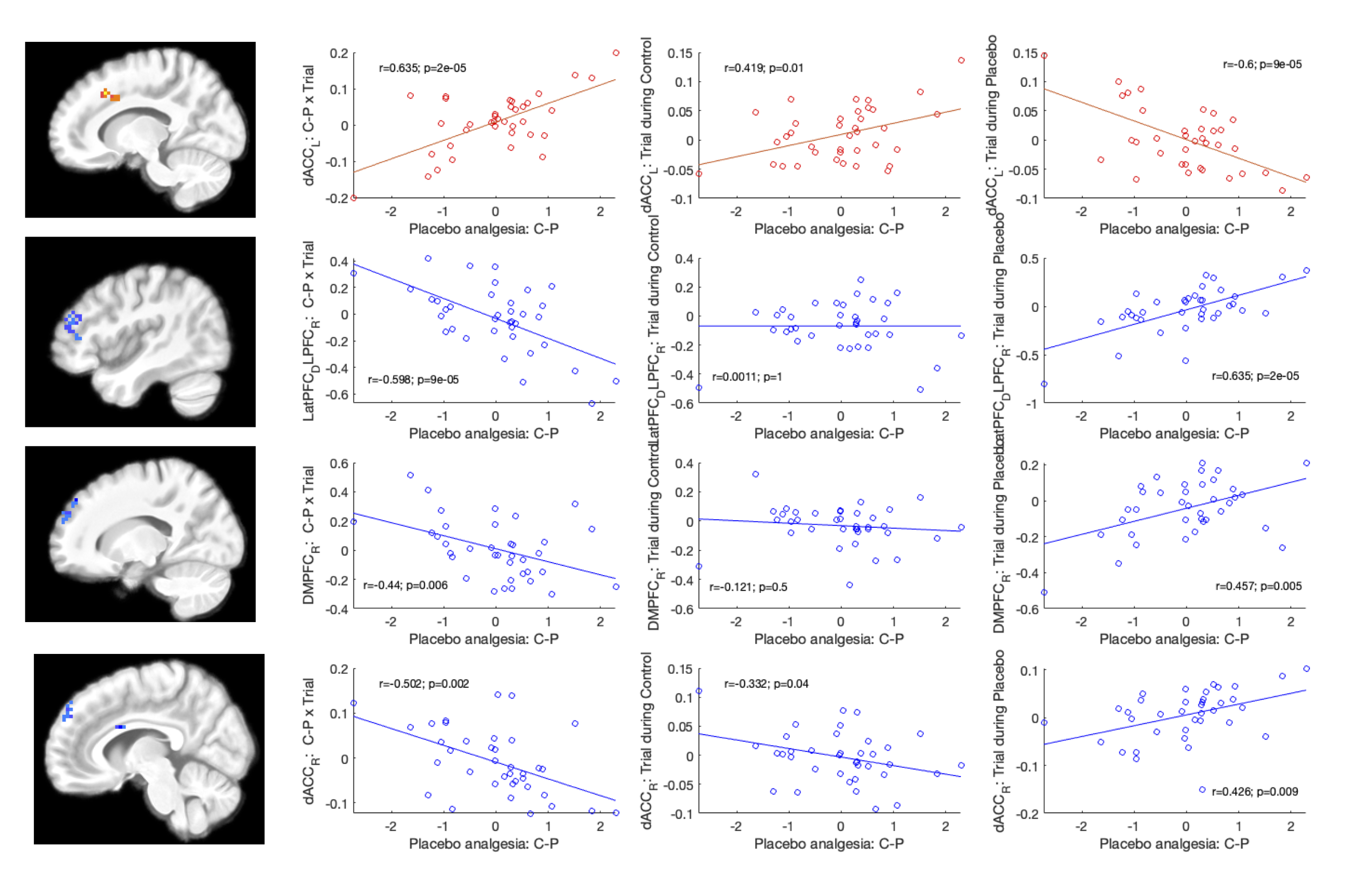

Supplement: Figure 8-1 — Relationship between placebo analgesia and changes over time. Treatment x Time interactions varied as a function of individual differences in placebo analgesia in the dorsal anterior cingulate (top row), right DLPFC (second row), right DLPFC (third row), and right DMPFC (bottom row). Graphs illustrate associations between placebo analgesia and a) the difference in changes over time between placebo and control blocks (left), b) changes over time during control blocks (middle); and c) changes over time during placebo blocks (right). Download Figure 8-1, TIF file. [file jneuro-45-e0050252025-s014.tif]
