# Supplementary material for: Isolating Brain Mechanisms of Expectancy Effects on Pain: Cue-Based Stimulus Expectancies versus Placebo-Based Treatment Expectancies
Source: J Neurosci. 2025 Jul 28;45(34):e0050252025. doi: 10.1523/JNEUROSCI.0050-25.2025 (PMC12369932; doi:10.1523/JNEUROSCI.0050-25.2025)
Supplement: Figure 3-1 — Effects of noxious stimulation on heat-evoked responses prior to treatment: Uncorrected results and correction within regions of interest. Download Figure 3-1, DOCX file. [file jneuro-45-e0050252025-s002.docx]

Extended Data Figure 3-1. Effects of noxious stimulation on heat-evoked responses prior to treatment: Uncorrected results and correction within regions of interest.^a^

| Analysis | Contrast | Anatomical label | x | y | z | # of voxels | Volume (mm^3^) | Max stat |
| --- | --- | --- | --- | --- | --- | --- | --- | --- |
| Whole brain cluster correction | High Heat > Low Heat | L Cerebellum Crus 1 | -34 | -64 | -28 | 106 | 2862 | 15.47 |
|  |  | L Cerebellum VIII | -4 | -62 | -32 | 49 | 1323 | 13.62 |
|  |  | R Anterior Insula Lobe | 46 | 16 | -2 | 31 | 837 | 14.56 |
|  |  | R Middle Frontal Gyrus (DLPFC) | 32 | 50 | 26 | 37 | 999 | 10.16 |
|  |  | Posterior Cingulate Cortex | 2 | -28 | 28 | 44 | 1188 | 11.04 |
|  | Low Heat > High Heat | R Medial Temporal Pole | 38 | 14 | -34 | 31 | 837 | 8.94 |
|  |  | R Hippocampus and amygdala | 28 | -16 | -20 | 144 | 3888 | 20 |
|  |  | L Superior Orbital Gyrus | -16 | 32 | -16 | 46 | 1242 | 13.64 |
|  |  | Area 25 (sgACC) | 2 | 14 | -14 | 35 | 945 | 11.4 |
|  |  | L Paracentral Lobule (Area 4a) | -4 | -26 | 58 | 33 | 891 | 14.4 |
| Pain placebo cluster correction | High Heat > Low Heat | L Cerebellum Crus 1 | -34 | -64 | -28 | 66 | 1782 | 15.47 |
|  |  | Cerebellar Vermis 8 | -4 | -62 | -28 | 33 | 891 | 13.62 |
|  |  | R Cerebellum Crus 1 | 38 | -56 | -32 | 14 | 378 | 12.26 |
|  |  | R Insula Lobe | 46 | 16 | -2 | 30 | 810 | 14.56 |
|  |  | R Caudate Nucleus / Thal: Prefrontal | 16 | -8 | 16 | 19 | 513 | 15.38 |
|  |  | R Insula Lobe | 38 | 10 | 10 | 13 | 351 | 8.93 |
|  |  | Posterior Cingulate Cortex | 2 | -26 | 28 | 39 | 1053 | 11.04 |
|  |  | R MCC, contiguous with DMPFC | 2 | 22 | 38 | 25 | 675 | 9.57 |
|  | Low Heat > High Heat | L Superior Orbital Gyrus (Area Fo1) | -14 | 34 | -20 | 11 | 297 | 13.64 |
| Uncorrected | High Heat > Low Heat | L Cerebellum VI | -26 | -64 | -28 | 555 | 14985 | 15.47 |
|  |  | R Cerebellum Crus 1 | 38 | -62 | -28 | 93 | 2511 | 12.26 |
|  |  | Area 44 | -50 | 16 | -4 | 29 | 783 | 11.98 |
|  |  | R IFG p. Opercularis | 44 | 14 | 4 | 135 | 3645 | 14.56 |
|  |  | R Thalamus, contiguous with caudate | 16 | -4 | 14 | 110 | 2970 | 15.38 |
|  |  | R Thalamus, contiguous with caudate | 28 | -16 | 8 | 19 | 513 | 8.52 |
|  |  | Thal: Premotor | -20 | -14 | 14 | 71 | 1917 | 7.57 |
|  |  | L ACC | -4 | 22 | 28 | 244 | 6588 | 13.08 |
|  |  | L Middle Frontal Gyrus | -32 | 46 | 26 | 141 | 3807 | 13.22 |
|  |  | R Middle Frontal Gyrus | 32 | 50 | 22 | 146 | 3942 | 10.16 |
|  |  | L Cuneus (Area hOc3d [V3d]) | -8 | -92 | 22 | 32 | 864 | 11.58 |
|  |  | Posterior Cingulate Cortex | 2 | -28 | 28 | 83 | 2241 | 11.04 |
|  |  | R SupraMarginal Gyrus (Area PF (IPL)) | 58 | -34 | 50 | 35 | 945 | 8.41 |
|  |  | R Posterior-Medial Frontal (DMPFC) | 14 | 10 | 68 | 23 | 621 | 8.75 |
|  | Low Heat > High Heat | R Hippocampus, contiguous with amgydala, temporal pole | 38 | -8 | -22 | 556 | 15012 | 20 |
|  |  | L Middle Temporal Gyrus | -50 | 8 | -26 | 169 | 4563 | 12.34 |
|  |  | L Hippocampus | -28 | -22 | -16 | 148 | 3996 | 14.02 |
|  |  | L Mid Orbital Gyrus (Area s32), contiguous with sgACC, VMPFC, VLPFC | -4 | 26 | -14 | 376 | 10152 | 13.64 |
|  |  | R Middle Temporal Gyrus | 52 | -64 | -2 | 175 | 4725 | 10.49 |
|  |  | L Middle Occipital Gyrus | -50 | -74 | 4 | 148 | 3996 | 9.08 |
|  |  | L Middle Temporal Gyrus (Area TE 3) | -64 | -14 | -8 | 19 | 513 | 8.84 |
|  |  | R Precuneus | 10 | -52 | 14 | 56 | 1512 | 7.75 |
|  |  | R Superior Temporal Gyrus (Area TE 3) | 68 | -22 | 8 | 14 | 378 | 7.74 |
|  |  | L Postcentral Gyrus (Area 4p ) | -56 | -10 | 34 | 141 | 3807 | 12.43 |
|  |  | L IFG p. Triangularis | -46 | 26 | 20 | 46 | 1242 | 8.55 |
|  |  | R Postcentral Gyrus | 62 | -8 | 28 | 71 | 1917 | 10.75 |
|  |  | RPrecentral Gyrus | 44 | -16 | 56 | 94 | 2538 | 10.2 |
|  |  | L Superior Frontal Gyrus | -22 | 32 | 56 | 12 | 324 | 8.97 |
|  |  | L Paracentral Lobule (Area 4a | -2 | -26 | 56 | 87 | 2349 | 14.4 |

^a^. This table presents results of robust regression for the contrast [High heat > Low heat] during the first three runs, prior to the treatment manipulation. See Table 2 in the main manuscript for results of whole-brain FDR-correction.
