# Supplementary material for: Isolating Brain Mechanisms of Expectancy Effects on Pain: Cue-Based Stimulus Expectancies versus Placebo-Based Treatment Expectancies
Source: J Neurosci. 2025 Jul 28;45(34):e0050252025. doi: 10.1523/JNEUROSCI.0050-25.2025 (PMC12369932; doi:10.1523/JNEUROSCI.0050-25.2025)
Supplement: Figure 3-3 — Effects of noxious stimulation on heat-evoked responses across all blocks. Download Figure 3-3, DOCX file. [file jneuro-45-e0050252025-s004.docx]

Extended Data Figure 3-3. Effects of noxious stimulation on heat-evoked responses across all blocks.^b^

| Analysis | Contrast | Anatomical Label | x | y | z | # of voxels | Volume (mm^3^) | Max stat |
| --- | --- | --- | --- | --- | --- | --- | --- | --- |
| Whole brain FDR | High Heat > Low Heat | R Cerebellum VIII | 26 | -74 | -58 | 5 | 135 | 6.22 |
|  |  | R Cerebellum VIII | 38 | -58 | -52 | 11 | 297 | 6.4 |
|  |  | L Cerebellum VIII | -34 | -56 | -52 | 14 | 378 | 8.09 |
|  |  | L Cerebellum VIII | -22 | -62 | -50 | 21 | 567 | 6.81 |
|  |  | Lobule IX Hem | -8 | -64 | -50 | 4 | 108 | 5.87 |
|  |  | Cerebellar Vermis 7, contiguous with bilateral cerebellum and occipital lobe | -4 | -64 | -26 | 1720 | 46440 | 16.76 |
|  |  | R Cerebellum Crus 2 | 38 | -80 | -40 | 12 | 324 | 7.49 |
|  |  | Pons | -8 | -22 | -46 | 3 | 81 | 5.79 |
|  |  | L Cerebellum Crus 2 | -32 | -86 | -40 | 4 | 108 | 8.75 |
|  |  | Lobule I IV Hem | 10 | -38 | -28 | 8 | 216 | 8.28 |
|  |  | L Cerebellum Crus 1 | -28 | -88 | -28 | 6 | 162 | 6.58 |
|  |  | R Inferior Temporal Gyrus | 56 | -52 | -28 | 3 | 81 | 6.28 |
|  |  | Lobule I IV Hem | 8 | -40 | -22 | 5 | 135 | 8.68 |
|  |  | R Putamen | 26 | -2 | 8 | 651 | 17577 | 12.72 |
|  |  | L Inferior Temporal Gyrus | -56 | -40 | -20 | 3 | 81 | 6.13 |
|  |  | R Middle Orbital Gyrus | 28 | 52 | -14 | 14 | 378 | 9.09 |
|  |  | L Superior Orbital Gyrus (Area Fp1) | -16 | 62 | -10 | 34 | 918 | 11.32 |
|  |  | Left Midbrain (substantia nigra) | -14 | -26 | -10 | 5 | 135 | 5.81 |
|  |  | R Putamen | 20 | 10 | -8 | 110 | 2970 | 12.88 |
|  |  | R Mid Orbital Gyrus (Area Fp1) | 8 | 62 | -14 | 3 | 81 | 6.39 |
|  |  | L Putamen, contiguous with insula, thalamus, operculum | -26 | 4 | 10 | 583 | 15741 | 14.36 |
|  |  | L Middle Orbital Gyrus | -38 | 56 | -10 | 20 | 540 | 9.81 |
|  |  | R Superior Orbital Gyrus (Area Fp1) | 22 | 64 | -10 | 8 | 216 | 8.63 |
|  |  | R Middle Frontal Gyrus | 50 | 50 | 2 | 33 | 891 | 11.01 |
|  |  | Cerebellar Vermis 4/5 | -2 | -50 | 2 | 5 | 135 | 5.72 |
|  |  | R Middle Frontal Gyrus | 40 | 46 | 16 | 47 | 1269 | 7.46 |
|  |  | L Thalamus | -8 | -8 | 4 | 3 | 81 | 7.74 |
|  |  | L Thalamus | -2 | -14 | 14 | 56 | 1512 | 8.54 |
|  |  | L Pregenual ACC | -8 | 34 | 8 | 8 | 216 | 7.51 |
|  |  | R Caudate Nucleus | 16 | 20 | 14 | 7 | 189 | 5.86 |
|  |  | R Rostral ACC | 8 | 32 | 20 | 5 | 135 | 6.35 |
|  |  | R Middle Frontal Gyrus (DLPFC) | 28 | 38 | 22 | 5 | 135 | 6.14 |
|  |  | L Middle Frontal Gyrus | -32 | 40 | 22 | 9 | 243 | 6.26 |
|  |  | Posterior Cingulate Cortex | -2 | -32 | 28 | 41 | 1107 | 7.88 |
|  |  | L SupraMarginal Gyrus (Area PF (IPL)) | -64 | -40 | 34 | 45 | 1215 | 10.89 |
|  |  | R MCC | 2 | -10 | 32 | 48 | 1296 | 9.99 |
|  |  | R Superior Medial Gyrus | 10 | 58 | 32 | 23 | 621 | 7.13 |
|  |  | L Precuneus | -10 | -70 | 34 | 66 | 1782 | 11.69 |
|  |  | R SupraMarginal Gyrus | 64 | -44 | 40 | 6 | 162 | 5.75 |
|  |  | L Middle Frontal Gyrus | -38 | 32 | 38 | 6 | 162 | 6.78 |
|  |  | L Angular Gyrus | -38 | -62 | 40 | 3 | 81 | 6.66 |
|  |  | R Middle Frontal Gyrus | 38 | 34 | 46 | 18 | 486 | 6.69 |
|  |  | L Superior Medial Gyrus | -8 | 46 | 50 | 3 | 81 | 5.95 |
|  | Low Heat > High Heat | L Middle Temporal Gyrus | -52 | -2 | -20 | 469 | 12663 | 12.4 |
|  |  | R Fusiform Gyrus | 34 | -50 | -4 | 1764 | 47628 | 18.47 |
|  |  | R Temporal Pole | 34 | -14 | -40 | 5 | 135 | 12.46 |
|  |  | R Inferior Temporal Gyrus | 46 | -22 | -28 | 7 | 189 | 7.06 |
|  |  | L Fusiform Gyrus | -32 | -32 | -16 | 144 | 3888 | 17.55 |
|  |  | L Rectal Gyrus (Area s32), contiguous with VMPFC, sgACC | -2 | 28 | -16 | 335 | 9045 | 13.44 |
|  |  | L Middle Occipital Gyrus | -46 | -74 | 2 | 311 | 8397 | 13.13 |
|  |  | L Lingual Gyrus | -16 | -50 | -4 | 4 | 108 | 6.45 |
|  |  | L Thalamus | -10 | -26 | -2 | 3 | 81 | 5.83 |
|  |  | L Thalamus | -14 | -28 | 4 | 5 | 135 | 6.78 |
|  |  | R Superior Temporal Gyrus | 50 | -34 | 10 | 19 | 513 | 8.69 |
|  |  | L Middle Occipital Gyrus | -38 | -82 | 14 | 4 | 108 | 6.45 |
|  |  | L Superior Temporal Gyrus | -44 | -38 | 16 | 34 | 918 | 9.94 |
|  |  | L Postcentral Gyrus (Area 1) | -62 | -10 | 34 | 66 | 1782 | 8.09 |
|  |  |  | 68 | -4 | 28 | 9 | 243 | 9.17 |
|  |  | L Middle Occipital Gyrus | -28 | -86 | 28 | 6 | 162 | 7.34 |
|  |  | L Inferior Parietal Lobule (Area 2) | -50 | -28 | 46 | 56 | 1512 | 7.57 |
|  |  | R Postcentral Gyrus (Area 4p) | 40 | -28 | 52 | 328 | 8856 | 12.85 |
|  |  | L Postcentral Gyrus | -34 | -16 | 44 | 4 | 108 | 6.17 |
|  |  | L Postcentral Gyrus (Area 2) | -34 | -38 | 52 | 76 | 2052 | 11.14 |
|  |  | L Paracentral Lobule (Area 4a) | -2 | -22 | 56 | 227 | 6129 | 14.96 |
|  |  | L Postcentral Gyrus (Area 1) | -56 | -16 | 46 | 3 | 81 | 5.79 |
|  |  | RPrecentral Gyrus | 52 | -14 | 46 | 4 | 108 | 6.74 |
| Uncorrected results | High Heat > Low Heat | Cerebellar Vermis 7, contiguous with midbrain and occipital lobe | -4 | -64 | -26 | 1720 | 46440 | 16.76 |
|  |  | R Putamen, contiguous with thalamus, caudate, and middle insula | 26 | -2 | 8 | 651 | 17577 | 12.72 |
|  |  | R Middle Orbital Gyrus | 28 | 52 | -14 | 14 | 378 | 9.09 |
|  |  | L Superior Orbital Gyrus / Area Fp1 | -16 | 62 | -10 | 34 | 918 | 11.32 |
|  |  | R Putamen | 20 | 10 | -8 | 110 | 2970 | 12.88 |
|  |  | L Putamen, contiguous with thalamus, caudate, and insula | -26 | 4 | 10 | 583 | 15741 | 14.36 |
|  |  | R Middle Frontal Gyrus | 40 | 46 | 16 | 47 | 1269 | 7.46 |
|  |  | L Thalamus | -2 | -14 | 14 | 56 | 1512 | 8.54 |
|  |  | L SupraMarginal Gyrus / Area PF (IPL) | -64 | -40 | 34 | 45 | 1215 | 10.89 |
|  |  | R MCC | 2 | -10 | 32 | 48 | 1296 | 9.99 |
|  |  | L Precuneus | -10 | -70 | 34 | 66 | 1782 | 11.69 |
|  | Low Heat > High Heat | L Middle Temporal Gyrus | -52 | -2 | -20 | 469 | 12663 | 12.4 |
|  |  | R Fusiform Gyrus, contiguous with occipital cortex, hippocampus, parahippocampal gyrus | 34 | -50 | -4 | 1764 | 47628 | 18.47 |
|  |  | L Fusiform Gyrus | -32 | -32 | -16 | 144 | 3888 | 17.55 |
|  |  | L Rectal Gyrus / Area s32, contiguous with VMPFC, sgACC, mOFC | -2 | 28 | -16 | 335 | 9045 | 13.44 |
|  |  | L Middle Occipital Gyrus / Area hOc4la | -46 | -74 | 2 | 311 | 8397 | 13.13 |
|  |  | R Superior Temporal Gyrus | 50 | -34 | 10 | 19 | 513 | 8.69 |
|  |  | L Superior Temporal Gyrus / Area PFcm (IPL) | -44 | -38 | 16 | 34 | 918 | 9.94 |
|  |  | L Postcentral Gyrus / Area 1 | -62 | -10 | 34 | 66 | 1782 | 8.09 |
|  |  | L Inferior Parietal Lobule / Area 2 | -50 | -28 | 46 | 56 | 1512 | 7.57 |
|  |  | R Postcentral Gyrus / Area 4p | 40 | -28 | 52 | 328 | 8856 | 12.85 |
|  |  | L Postcentral Gyrus / Area 2 | -34 | -38 | 52 | 76 | 2052 | 11.14 |
|  |  | L Paracentral Lobule / Area 4a | -2 | -22 | 56 | 227 | 6129 | 14.96 |

^b^. This table presents results using robust regression for the contrast [High heat > Low heat] across all blocks.
