# Supplementary material for: Isolating Brain Mechanisms of Expectancy Effects on Pain: Cue-Based Stimulus Expectancies versus Placebo-Based Treatment Expectancies
Source: J Neurosci. 2025 Jul 28;45(34):e0050252025. doi: 10.1523/JNEUROSCI.0050-25.2025 (PMC12369932; doi:10.1523/JNEUROSCI.0050-25.2025)
Supplement: Figure 3-4 — Preregistered neuroimaging analyses and implementation. Download Figure 3-4, DOCX file. [file jneuro-45-e0050252025-s005.docx]

Extended Data Figure 3-4. Preregistered neuroimaging analyses and implementation.

| **Preregistered analysis Number** | **Analysis Description** | **Preregistered hypothesis** | **Contrast / Implementation** | **Deviations and/or details not specified during preregistration** | **Was hypothesis supported?** |
| --- | --- | --- | --- | --- | --- |
| 1 | Treatment expectancy during heat pain: Placebo vs Control | P<C in PPN (Ains, Pins, Thal, SII, SMA, Cerebellum), amygdala, putamen | [Control > Placebo] implemented with robust regression; Path A in treatment expectancy mediation | Temperature and cue weren't specified; focusing on medium uncued trials to isolate pure placebo effect; response across temperatures reported in extended data | Only cerebellum showed a main effect; no correlations within PPN |
|  |  | P>C in regions involved in top down control (dlPFC/vlPFC/OFC, PAG, rdACC, pgACC/rACC) |  |  | Somewhat - all prefrontal, including DLPFC, but no PAG or OFC |
| 2 | Stimulus expectancy during heat pain | HM > LM in PPN/NPS (AIns, Pins, Thal, ACC, SII, SMA, Cerebellum), limbic regions (amygdala, hypothalamus), & regions involved in prediction error/aversive learning (ventral striatum, caudate, putamen) | HM vs LM | Treatment context wasn't specified; focusing on baseline only | Yes, based on Path A: Within PPN, saw DMPFC, PreSMA, mediation by CB; Whole brain included more of PPN, including Ains, SII, midIns, ACC, SII, SMA, Putamen, Caudate; no amygdala |
|  |  | LM > HM in regions that show deactivation in HvL contrast (precuneus, parahippocampal gyrus, vmPFC, OFC) |  |  | No (no regions survived whole brain correction) |
|  |  | (HM+LM)> nM in regions responsible for PE (VS, caudate, putamen) | [HM + LM] > nM | Uncued trials only present during treatment manipulation blocks | Yes (caud and amygdala) |
| 3 | Stimulus x treatment expectancy during heat pain (during crossover trials) | (P+HM+C+LM)<(P+LM+C+HM) in regions involved in top-down control (dlPFC/vlPFC/OFC, dlPFC) | (P+HM+C+LM) vs (P+LM+C+HM) | Temperature wasn't specified; focusing on medium trials to isolate pure expectancy effects | Somewhat (results from negative effect for rob5 x placbeo) - we do see VLPFC, DLPFC, DMPFC, but we also see these for positive effect |
|  |  | (P+HM+C+LM)>(P+LM+C+HM) in PPN/NPS (esp insula) |  |  | Somewhat (results from positive effect for rob5 x placbeo) - we see insula, SII, but lots of other regions, only parahipp and sup temp within HvL regions |
| 4 | Time (Trial within condition) as Pmod | (C+Early+P+Late)>(C+Late+P+Early) : Less activation of regions involved in placebo (dlPFC/vlPFC/OFC, dlPFC, PAG, rdACC, pgACC/rACC, ventral striatum, caudate) during heat in later relative to earlier control trials | [Control x Time] vs [Placebo x Time] |  | Yes in VLPFC and dACC if we consider association with placebo analgesia; main effects and order effects do not survive multiple comparisons correction |
| 5 | Behavioral placebo response (mean difference in pain on P relative to C+baseline trials, excluding conditioning) as continuous moderator | Placebo and interaction effects will correlate with behavioral placebo response | Placebo x behavior | Temperature wasn't specified; We didn't include baseline in control contrast | Yes, for all analyses related to treatment expectancy, the only results that survived FDR-correction involved correlations with placebo analgesia |
| 6a | Stimulus expectancy mediation | PPN/NPS and regions involved in PE / learning will be mediators of stim, but not treatment, modulation of pain | Mediation | We did not test for a formal interaction | Somewhat; we did not see mediation by NPS, SIIPS, or regions in nociceptive networks, but we did see mediation by caudate |
| 6b | Treatment expectancy mediation | shouldn't be mediated by PPN or regions involved in learning | Mediation |  | No, we observed mediation by striatum, insula, and PAG which are all implicated in learning |
| 6c | Interaction mediation | shouldn't be mediated by PPN or regions involved in learning | Mediation | Our software does not test for mediators of an interaction term; analysis not completed | N/A |
| 7 | Background connectivity | residual activity in rACC will correlate with residual activity in PAG more in placebo relative to control (greater background connectivity); specifically on volumes where resid. activity not diff. from baseline (avg. activity in last 2 TRs) | evaluate rACC-PAG connectivity under placebo and control after regressing out the design matrix (cue, heat, scale/rating, TRs for scanner stabilization,&last 2 TRs for baseline measure) and nuisance parameters |  | No; although there was a negative relationship between rACC and PAG residual activation, associations did not differ as a function of treatment expectancy |
