# Supplementary material for: Isolating Brain Mechanisms of Expectancy Effects on Pain: Cue-Based Stimulus Expectancies versus Placebo-Based Treatment Expectancies
Source: J Neurosci. 2025 Jul 28;45(34):e0050252025. doi: 10.1523/JNEUROSCI.0050-25.2025 (PMC12369932; doi:10.1523/JNEUROSCI.0050-25.2025)
Supplement: Figure 4-1 — Brain mediators of cue-based stimulus expectancies: Uncorrected results and correction within regions of interest. Download Figure 4-1, DOCX file. [file jneuro-45-e0050252025-s006.docx]

Extended Data Figure 4-1. Brain mediators of cue-based stimulus expectancies: Uncorrected results and correction within regions of interest.^c^

| Analysis | Contrast | Anatomical label | x | y | z | # of voxels | Volume (mm^3^) | Max stat |
| --- | --- | --- | --- | --- | --- | --- | --- | --- |
| FDR Correction within nociceptive regions | Path A pos | R Superior Frontal Gyrus (DMPFC) | 22 | 44 | 22 | 1 | 27 | 10.85 |
|  |  | L Superior Medial Gyrus (DMPFC) | 2 | 32 | 38 | 12 | 324 | 15.01 |
|  |  | R Posterior-Medial Frontal / PreSMA | 8 | -22 | 58 | 28 | 756 | 12.04 |
|  | Path A neg | Nothing survives |  |  |  |  |  |  |
|  | Path B pos | R Insula Lobe | 38 | 10 | 4 | 11 | 297 | 13.29 |
|  | Path B neg | Nothing survives |  |  |  |  |  |  |
|  | Path AB pos | Cerebellar Vermis 9 | 2 | -58 | -34 | 2 | 54 | 22.57 |
| Correction within Pain and Placebo Regions | Path A pos | R Rolandic Operculum / SII | 50 | -8 | 14 | 19 | 513 | 12.47 |
|  |  | R IFG p. Opercularis / BA44 | 52 | 14 | 8 | 10 | 270 | 11.74 |
|  | Path A neg | Nothing survives |  |  |  |  |  |  |
|  | Path B pos | Nothing survives |  |  |  |  |  |  |
|  | Path B neg | Nothing survives |  |  |  |  |  |  |
|  | Path AB pos | Nothing survives |  |  |  |  |  |  |
|  | Path AB neg | Nothing survives |  |  |  |  |  |  |
| Whole brain cluster correction | Path A pos | L Superior Medial Gyrus (DMPFC) | 2 | 32 | 50 | 79 | 2133 | 15.01 |
|  |  | R Posterior-Medial Frontal / PreSMA | 8 | -22 | 62 | 37 | 999 | 16.97 |
|  | Path A neg | Nothing survives |  |  |  |  |  |  |
|  | Path B pos | L Putamen, contiguous with caudate | -20 | 14 | 4 | 24 | 648 | 12.5 |
|  |  | L Superior Frontal Gyrus (DMPFC) | -22 | 4 | 56 | 18 | 486 | 11.15 |
|  | Path B neg | Nothing survives |  |  |  |  |  |  |
|  | Path AB pos | L Middle Occipital Gyrus (Area hOc3d [V3d]) | -20 | -100 | 10 | 8 | 216 | 10.95 |
|  |  | L Middle Occipital Gyrus | -28 | -86 | 20 | 12 | 324 | 9.92 |
|  |  | L Posterior-Medial Frontal | -2 | 14 | 68 | 14 | 378 | 10.76 |
|  | Path AB neg | Nothing survives |  |  |  |  |  |  |
| Uncorrected | Path A pos | R Cerebellum IX | 10 | -58 | -56 | 12 | 324 | 9.22 |
|  |  | L Superior Orbital Gyrus (Area Fo3 ) | -16 | 32 | -26 | 8 | 216 | 9.56 |
|  |  | L Inferior Temporal Gyrus | -68 | -32 | -20 | 10 | 270 | 11.49 |
|  |  | R Insula Lobe | 40 | 26 | -4 | 121 | 3267 | 13.61 |
|  |  | R Middle Temporal Gyrus | 58 | -50 | -4 | 41 | 1107 | 13.93 |
|  |  | R Superior Temporal Gyrus | 58 | -22 | -4 | 43 | 1161 | 14.95 |
|  |  | L IFG p. Orbitalis | -38 | 52 | -14 | 10 | 270 | 10.7 |
|  |  | Cerebellar Vermis 3 | 4 | -44 | -8 | 30 | 810 | 9.38 |
|  |  | R Lingual Gyrus (Area hOc1 [V1]) | 16 | -64 | 2 | 32 | 864 | 12.14 |
|  |  | L Middle Temporal Gyrus | -70 | -38 | -4 | 8 | 216 | 15.41 |
|  |  | L IFG p. Triangularis | -44 | 16 | 2 | 58 | 1566 | 11.51 |
|  |  | Midbrain surrounding PAG | 2 | -22 | -2 | 16 | 432 | 10.36 |
|  |  | R IFG p. Opercularis (BA44) | 52 | 14 | 14 | 119 | 3213 | 12.9 |
|  |  | R Caudate Nucleus | 14 | 16 | 2 | 32 | 864 | 10.42 |
|  |  | R Pallidum | 22 | 4 | 4 | 10 | 270 | 12.75 |
|  |  | R Rolandic Operculum (Area OP3 [VS]) / dpInsula / SII | 46 | -10 | 14 | 107 | 2889 | 12.47 |
|  |  | R Insula Lobe | 34 | 22 | 8 | 34 | 918 | 14.3 |
|  |  | R Middle Frontal Gyrus | 32 | 44 | 28 | 89 | 2403 | 10.86 |
|  |  | L Superior Medial Gyrus | 2 | 38 | 38 | 349 | 9423 | 15.01 |
|  |  | R ACC (Area 33 ) | 4 | 16 | 22 | 14 | 378 | 19.81 |
|  |  | L MCC | -8 | 8 | 32 | 13 | 351 | 14.34 |
|  |  | R Superior Frontal Gyrus | 20 | 58 | 32 | 23 | 621 | 10.16 |
|  |  | R MCC | 8 | -2 | 34 | 26 | 702 | 10.51 |
|  |  | L IFG p. Opercularis (Area 44 ) | -46 | 8 | 28 | 7 | 189 | 9.4 |
|  |  | R Inferior Parietal Lobule (Area PGa (IPL)) | 52 | -52 | 38 | 19 | 513 | 8.12 |
|  |  | RPrecentral Gyrus | 38 | 2 | 50 | 88 | 2376 | 13.05 |
|  |  | L Angular Gyrus (Area PGa (IPL)) | -52 | -64 | 38 | 15 | 405 | 9.75 |
|  |  | R MCC | 10 | 20 | 38 | 11 | 297 | 8.88 |
|  |  | R Middle Frontal Gyrus | 46 | 14 | 44 | 11 | 297 | 9.62 |
|  |  | R Posterior-Medial Frontal | 8 | -22 | 58 | 87 | 2349 | 16.97 |
|  |  | R Superior Frontal Gyrus | 20 | 20 | 46 | 14 | 378 | 14.04 |
|  |  | L Middle Frontal Gyrus | -38 | 26 | 50 | 13 | 351 | 11.03 |
|  |  | R Superior Frontal Gyrus | 22 | 22 | 64 | 3 | 81 | 8.86 |
|  | Path A neg | R Hippocampus, amygdala | 22 | -8 | -16 | 23 | 621 | 8.65 |
|  |  | L Middle Temporal Gyrus | -52 | -16 | -8 | 24 | 648 | 8.42 |
|  |  | L Posterior Cingulate Cortex | -22 | -34 | 28 | 12 | 324 | 8.23 |
|  |  | L Cuneus | 2 | -92 | 32 | 9 | 243 | 8.71 |
|  | Path B pos | L Cerebellum VII | -26 | -68 | -44 | 25 | 675 | 17.01 |
|  |  | Pons | -2 | -34 | -40 | 15 | 405 | 8.46 |
|  |  | L Pons | -14 | -32 | -34 | 7 | 189 | 10.13 |
|  |  | L Cerebellum VI | -8 | -64 | -26 | 24 | 648 | 9.94 |
|  |  | R Cerebellum VI | 28 | -56 | -26 | 13 | 351 | 8.25 |
|  |  | L Cerebellum VI | -22 | -62 | -22 | 7 | 189 | 11.18 |
|  |  | L Olfactory cortex | -22 | 8 | -14 | 9 | 243 | 8.89 |
|  |  | R Putamen | 16 | 8 | -8 | 18 | 486 | 13.24 |
|  |  | L Anterior Insula | -22 | 26 | -4 | 32 | 864 | 11.64 |
|  |  | Thal: Parietal | -20 | -26 | -4 | 9 | 243 | 11.1 |
|  |  | L Putamen | -28 | 10 | 4 | 93 | 2511 | 15.77 |
|  |  | L Superior Orbital Gyrus | -22 | 52 | -2 | 11 | 297 | 12.3 |
|  |  | R Insula Lobe | 38 | 4 | 10 | 32 | 864 | 13.29 |
|  |  | R IFG p. Opercularis (BA44) | 50 | 8 | 20 | 30 | 810 | 10.07 |
|  |  | L Rolandic Operculum | -44 | 2 | 16 | 16 | 432 | 9.14 |
|  |  | R ACC | 16 | 34 | 16 | 9 | 243 | 9.49 |
|  |  | L Superior Parietal Lobule (Area 7A (SPL)) | -20 | -70 | 46 | 13 | 351 | 9.37 |
|  |  | L Middle Frontal Gyrus | -26 | 2 | 56 | 46 | 1242 | 11.15 |
|  | Path B neg | R Fusiform Gyrus | 44 | -28 | -26 | 7 | 189 | 8.45 |
|  |  | R Operculum | 38 | -8 | -10 | 15 | 405 | 7.46 |
|  |  | R Precuneus | 8 | -52 | 10 | 82 | 2214 | 9.55 |
|  |  | R Middle Temporal Gyrus | 52 | -58 | 14 | 66 | 1782 | 8.78 |
|  |  | L Middle Occipital Gyrus (Area PGp (IPL)) | -40 | -74 | 28 | 24 | 648 | 8.3 |
|  |  | R Precuneus | 2 | -50 | 46 | 22 | 594 | 8.53 |
|  |  | L Paracentral Lobule (Area 4a ) | -2 | -34 | 58 | 65 | 1755 | 9.38 |
|  | Path AB pos | R Cerebellum IX | 14 | -40 | -46 | 7 | 189 | 8.88 |
|  |  | R Putamen | 32 | -4 | -8 | 5 | 135 | 11.01 |
|  |  | R Middle Temporal Gyrus | 58 | -56 | -2 | 7 | 189 | 11.26 |
|  |  | R Lingual Gyrus | 8 | -50 | 2 | 26 | 702 | 10.73 |
|  |  | R Calcarine Gyrus (Area hOc1 [V1]) | 16 | -92 | 2 | 24 | 648 | 12.05 |
|  |  | L Middle Occipital Gyrus (Area hOc3d [V3d]) | -22 | -98 | 14 | 31 | 837 | 10.95 |
|  |  | L Middle Occipital Gyrus (Area hOc4lp) | -32 | -86 | 20 | 30 | 810 | 9.92 |
|  |  | L Precentral Gyrus / BA44 | -50 | 2 | 20 | 5 | 135 | 11.54 |
|  |  | L Precentral Gyrus | -46 | 8 | 38 | 11 | 297 | 10.9 |
|  |  | L Superior Occipital Gyrus (Area hOc4d [V3A]) | -10 | -88 | 40 | 6 | 162 | 12.94 |
|  |  | L Posterior-Medial Frontal | -2 | 14 | 64 | 32 | 864 | 10.76 |
|  |  | L Precentral Gyrus | -38 | -2 | 62 | 9 | 243 | 12.47 |
|  |  | R Superior Frontal Gyrus | 28 | 20 | 62 | 10 | 270 | 12.34 |
|  | Path AB neg | R IFG p. Orbitalis | 38 | 32 | -20 | 7 | 189 | 8.32 |
|  |  | R Middle Temporal Gyrus (Area PGa (IPL)) | 58 | -56 | 10 | 12 | 324 | 9.79 |
|  |  | L SupraMarginal Gyrus (Area PF (IPL)) | -64 | -38 | 32 | 13 | 351 | 7.93 |
|  |  | L Middle Cingulate Cortex | -16 | -14 | 34 | 4 | 108 | 8.08 |

^c^. This table reports results of voxel-wise multilevel mediation searching for mediators of the association between pain-predictive cue (X: [High > Low]) and pain on medium heat trials prior to the treatment manipulation. For whole-brain FDR correction, see Table 3 in the main manuscript.
