# Supplementary material for: Isolating Brain Mechanisms of Expectancy Effects on Pain: Cue-Based Stimulus Expectancies versus Placebo-Based Treatment Expectancies
Source: J Neurosci. 2025 Jul 28;45(34):e0050252025. doi: 10.1523/JNEUROSCI.0050-25.2025 (PMC12369932; doi:10.1523/JNEUROSCI.0050-25.2025)
Supplement: Figure 5-1 — Treatment expectancy effects on brain responses to medium heat and associations with placebo analgesia. Download Figure 5-1, DOCX file. [file jneuro-45-e0050252025-s007.docx]

Extended Data Figure 5-1. Treatment expectancy effects on brain responses to medium heat and associations with placebo analgesia.^d^

| Contrast | Anatomical label | x | y | z | # of voxels | Volume (mm^3^) | maxstat |
| --- | --- | --- | --- | --- | --- | --- | --- |
| Positive main effect | L Cerebellum Crus 1^e^ | -34 | -58 | -32 | 32 | 864 | 12.49 |
|  | L Middle Occipital Gyrus (Area hOc3v [V3v]) | -20 | -100 | -2 | 28 | 756 | 8.73 |
|  | R Precuneus | 10 | -56 | 40 | 16 | 432 | 8.28 |
|  | L S1 | -64 | -28 | 44 | 22 | 594 | 7.75 |
| Negative main effect | R DLPFC / IFG | 34 | 14 | 22 | 14 | 378 | 7.65 |
|  | L Middle Frontal Gyrus | -50 | 16 | 38 | 17 | 459 | 8.4 |
| Positive association | R Cerebellum IX | 20 | -46 | -50 | 4 | 108 | 16.43 |
|  | R Hippocampus | 20 | -22 | -14 | 5 | 135 | 10.6 |
|  | R ParaHippocampal Gyrus | 32 | -44 | -4 | 12 | 324 | 8.56 |
|  | L Precuneus (Area 3a) | -16 | -40 | 56 | 22 | 594 | 15.25 |
| Negative association | Lobule IX Hem | -2 | -62 | -50 | 34 | 918 | 10.2 |
|  | L Middle Temporal Gyrus | -64 | -34 | -14 | 7 | 189 | 8.75 |
|  | R Mid Orbital Gyrus (Area Fp1) | 14 | 62 | -10 | 30 | 810 | 11.49 |
|  | L IFG p. Orbitalis | -46 | 38 | -4 | 23 | 621 | 12.05 |
|  | R Superior Orbital Gyrus (Area Fp1) | 32 | 58 | -4 | 42 | 1134 | 11.1 |
|  | L Superior Frontal Gyrus (Area Fp1) | -32 | 62 | -2 | 37 | 999 | 11.62 |
|  | R Superior Medial Gyrus | 10 | 50 | 16 | 24 | 648 | 11.62 |
|  | L Superior Frontal Gyrus | -14 | 56 | 16 | 15 | 405 | 9.24 |
|  | L Superior Frontal Gyrus | -20 | 56 | 26 | 15 | 405 | 9.57 |
|  | L Superior Medial Gyrus | -2 | 32 | 32 | 8 | 216 | 12.09 |
|  | R IFG p. Opercularis | 46 | 16 | 38 | 56 | 1512 | 10.7 |
|  | R Superior Medial Gyrus | 4 | 32 | 50 | 51 | 1377 | 13.79 |
|  | R Postcentral Gyrus (Area 1) | 52 | -20 | 46 | 22 | 594 | 8.54 |

^d^. This table presents uncorrected results of robust regression evaluating pure treatment expectancy effects on heat-evoked activation (uncued medium trials: [Control-Placebo]). We report main effects and associations with the magnitude of placebo analgesia (controlling for counterbalanced order). Whole-brain FDR-corrected results for associations with placebo analgesia are reported in Table 4 in the main manuscript.

^e^. This cluster was the only region that survived correction for multiple comparisons a) within nociceptive regions and b) within *a priori* regions involved in pain and placebo.
