# Supplementary material for: Isolating Brain Mechanisms of Expectancy Effects on Pain: Cue-Based Stimulus Expectancies versus Placebo-Based Treatment Expectancies
Source: J Neurosci. 2025 Jul 28;45(34):e0050252025. doi: 10.1523/JNEUROSCI.0050-25.2025 (PMC12369932; doi:10.1523/JNEUROSCI.0050-25.2025)
Supplement: Figure 5-3 — Treatment expectancy effects across temperatures. Download Figure 5-3, DOCX file. [file jneuro-45-e0050252025-s009.docx]

Extended Data Figure 5-3. Treatment expectancy effects across temperatures.^f^

| Analysis | Contrast | Anatomical Label | x | y | z | # of voxels | Volume (mm^3^) | Max stat |
| --- | --- | --- | --- | --- | --- | --- | --- | --- |
| Whole brain FDR | Main effect pos (C>P) | Nothing survives |  |  |  |  |  |  |
|  | Main effect neg (P>C) | Nothing survives |  |  |  |  |  |  |
|  | Positive Association | R Superior Temporal Sulcus | 38 | -52 | 16 | 66 | 1782 | 14.57 |
|  | Negative Association | Nothing survives |  |  |  |  |  |  |
| Correction within nociceptive regions | Main effect pos (C>P) | Nothing survives |  |  |  |  |  |  |
|  | Main effect neg (P>C) | L Caudate Nucleus | -20 | 16 | 14 | 4 | 108 | 11.31 |
|  | Positive Association | Nothing survives |  |  |  |  |  |  |
|  | Negative Association | Nothing survives |  |  |  |  |  |  |
| Uncorrected | Main effect pos (C>P) | Nothing survives |  |  |  |  |  |  |
|  | Main effect neg (P>C) | R Insula Lobe / Area Fo3 , contiguous with VLPFC | 34 | 22 | -20 | 25 | 675 | 10.16 |
|  |  | L Putamen / Caudate | -16 | 14 | 2 | 53 | 1431 | 11.31 |
|  |  | R dACC | 14 | -2 | 38 | 5 | 135 | 7.68 |
|  | Positive Association | R Cerebellum VI | 22 | -56 | -26 | 21 | 567 | 11.82 |
|  |  | Cerebellar Vermis 6 | -2 | -70 | -22 | 19 | 513 | 10.49 |
|  |  | L Middle Occipital Gyrus / Area hOc4la | -46 | -76 | 4 | 18 | 486 | 8.43 |
|  |  | R Occipital Cortex | 44 | -56 | -2 | 7 | 189 | 9.93 |
|  |  | L Superior Occipital Gyrus / Area hOc4d [V3A] | -14 | -86 | 20 | 62 | 1674 | 9.16 |
|  |  | R Occipital Cortex | 38 | -52 | 16 | 66 | 1782 | 14.57 |
|  |  | R Superior Occipital Gyrus | 22 | -76 | 32 | 29 | 783 | 10.59 |
|  |  | L Inferior Parietal Lobule / Area PFt (IPL) | -52 | -34 | 46 | 4 | 108 | 9.35 |
|  | Negative Association | Cerebellar Vermis 10 | 2 | -44 | -34 | 28 | 756 | 10.14 |
|  |  | L Middle Temporal Gyrus | -58 | 2 | -28 | 10 | 270 | 9.4 |
|  |  | R Inferior Temporal Gyrus | 64 | -34 | -20 | 7 | 189 | 8.58 |
|  |  | L Superior Temporal Gyrus / Area PF (IPL) | -64 | -34 | 20 | 20 | 540 | 9.89 |
|  |  | L Caudate Nucleus | -14 | -2 | 20 | 7 | 189 | 7.42 |

^f^. This table presents results of robust regression evaluating associations between pure treatment expectancy effects on heat-evoked activation on uncued trials across all temperatures ( [Control-Placebo]) and the magnitude of placebo analgesia (controlling for counterbalanced order).
