# Supplementary material for: Isolating Brain Mechanisms of Expectancy Effects on Pain: Cue-Based Stimulus Expectancies versus Placebo-Based Treatment Expectancies
Source: J Neurosci. 2025 Jul 28;45(34):e0050252025. doi: 10.1523/JNEUROSCI.0050-25.2025 (PMC12369932; doi:10.1523/JNEUROSCI.0050-25.2025)
Supplement: Figure 6-1 — Voxel-wise mediators of treatment expectancy effects on pain: Uncorrected results. Download Figure 6-1, DOCX file. [file jneuro-45-e0050252025-s010.docx]

Extended Data Figure 6-1. Voxel-wise mediators of treatment expectancy effects on pain: Uncorrected results.^g^

| Contrast | Anatomical label | x | y | z | voxels | volume_mm3 | maxstat |
| --- | --- | --- | --- | --- | --- | --- | --- |
| Path A pos | R Cerebellum Crus 2 | 44 | -46 | -46 | 3 | 81 | 8.93 |
|  | L IFG p. Orbitalis (Area Fo3) | -22 | 22 | -22 | 12 | 324 | 10.8 |
|  | R Fusiform Gyrus (Area hOc4v [V4(v)]) | 34 | -76 | -16 | 6 | 162 | 10.95 |
|  | L Inferior Temporal Gyrus (Area FG4) | -50 | -46 | -16 | 8 | 216 | 9.43 |
|  | L Middle Orbital Gyrus (Area Fo3) | -26 | 34 | -14 | 34 | 918 | 10.86 |
|  | L Posterior Cingulate Cortex | -10 | -28 | 28 | 12 | 324 | 10.25 |
|  | L SMA | -20 | -52 | 40 | 15 | 405 | 8.56 |
|  | L Superior Parietal Lobule | -62 | -32 | 44 | 19 | 513 | 10.8 |
|  | L MCC (Area 5M (SPL)) | -8 | -32 | 44 | 11 | 297 | 9.55 |
|  | L Superior Parietal Lobule | -56 | -46 | 52 | 23 | 621 | 9.17 |
| Path A neg | L Middle Temporal Gyrus | -56 | -38 | -4 | 11 | 297 | 8.63 |
|  | L Middle Frontal Gyrus | -52 | 20 | 40 | 19 | 513 | 8.95 |
| Path B pos | L Cerebellum VII | -22 | -74 | -52 | 127 | 3429 | 10.51 |
|  | R Cerebellum VIII | 32 | -70 | -52 | 88 | 2376 | 11.36 |
|  | Lobule VIIb Hem | -8 | -80 | -46 | 7 | 189 | 7.48 |
|  | R Cerebellum Crus 2 | 8 | -88 | -32 | 14 | 378 | 10.47 |
|  | L Cerebellum VI | -38 | -62 | -26 | 108 | 2916 | 11.1 |
|  | R Cerebellum Crus 1 | 40 | -64 | -28 | 36 | 972 | 18.67 |
|  | Area Fo2 / sgACC / OFC | 10 | 28 | -28 | 16 | 432 | 12.26 |
|  | R Middle Orbital Gyrus (Area Fp1) | 20 | 62 | -16 | 19 | 513 | 11.14 |
|  | L Putamen, contiguous with ventral striatum | -22 | 14 | -4 | 24 | 648 | 9.54 |
|  | R Anterior Insula / Operculum | 50 | 16 | -4 | 52 | 1404 | 8.52 |
|  | R Middle Temporal Gyrus | 52 | -40 | 8 | 21 | 567 | 8.09 |
|  | R Precuneus | 10 | -68 | 28 | 150 | 4050 | 10.74 |
|  | L Caudate Nucleus | -16 | 8 | 16 | 23 | 621 | 10.75 |
|  | R Rolandic Operculum (Area PFop (IPL)) | 58 | -16 | 20 | 31 | 837 | 11.09 |
|  | R Putamen, contiguous with R Middle Insula | 28 | 2 | 14 | 23 | 621 | 10.07 |
|  | R IFG p. Opercularis (Area 44) | 58 | 10 | 16 | 11 | 297 | 18.85 |
|  | Posterior Cingulate Cortex | 2 | -28 | 28 | 93 | 2511 | 11.04 |
|  | L Caudate | -20 | -4 | 22 | 12 | 324 | 11.08 |
|  | RPrecentral Gyrus | 52 | 4 | 28 | 10 | 270 | 8.17 |
|  | R Middle Frontal Gyrus | 38 | 22 | 44 | 206 | 5562 | 12.08 |
|  | L MCC | -2 | 4 | 34 | 37 | 999 | 12.26 |
|  | L Superior Medial Gyrus | 2 | 32 | 32 | 38 | 1026 | 8.2 |
|  | R Superior Frontal Gyrus | 20 | 58 | 32 | 28 | 756 | 10.42 |
|  | L Inferior Parietal Lobule (Area hIP1 (IPS)) | -38 | -52 | 46 | 261 | 7047 | 15.44 |
|  | R MCC | 10 | 16 | 40 | 24 | 648 | 10.2 |
|  | R Inferior Parietal Lobule (Area hIP2 (IPS)) | 46 | -46 | 50 | 108 | 2916 | 13.86 |
|  | RPrecentral Gyrus | 56 | 8 | 44 | 9 | 243 | 8.94 |
|  | L Posterior-Medial Frontal / DMPFC | -8 | 14 | 58 | 306 | 8262 | 12.98 |
| Path B neg | R Middle Temporal Gyrus | 58 | 2 | -26 | 23 | 621 | 8.86 |
|  | L Temporal Pole | -46 | 22 | -26 | 19 | 513 | 8.61 |
|  | L rACC, contiguous with sgACC, VMPFC, MPFC | -10 | 38 | -4 | 223 | 6021 | 8.87 |
|  | L IFG p. Orbitalis | -34 | 38 | -10 | 28 | 756 | 7.61 |
|  | R Posterior Hippocampus | 34 | -40 | -4 | 38 | 1026 | 8.48 |
|  | R Middle Occipital Gyrus (Area hOc4la) | 40 | -80 | 4 | 99 | 2673 | 8.51 |
|  | R Superior Medial Gyrus (Area Fp2) | 8 | 58 | 4 | 51 | 1377 | 9.02 |
| Path AB pos | L Cerebellum IX | -20 | -44 | -52 | 12 | 324 | 9.2 |
|  | Lobule VIIIb Verm | -2 | -64 | -46 | 14 | 378 | 9.09 |
|  | L Cerebellum Crus 1 | -38 | -44 | -40 | 4 | 108 | 7.83 |
|  | R Ventral Striatum / extended amygdala | 8 | 2 | -8 | 5 | 135 | 9.38 |
|  | L Putamen | -26 | 10 | 2 | 10 | 270 | 9.89 |
|  | R Calcarine Gyrus (Area hOc1 [V1]) | 26 | -70 | 4 | 21 | 567 | 9.97 |
|  | R Caudate Nucleus | 16 | 14 | 8 | 12 | 324 | 10.99 |
|  | R Superior Medial Gyrus (Area Fp1) | 10 | 62 | 14 | 21 | 567 | 8.33 |
|  | R Middle Frontal Gyrus | 34 | 56 | 14 | 10 | 270 | 9.3 |
|  | R Calcarine Gyrus (Area hOc3d [V3d]) | 10 | -74 | 16 | 28 | 756 | 10.67 |
|  | R Cuneus | 16 | -82 | 38 | 29 | 783 | 9.54 |
| Path AB neg | L Precuneus | -2 | -58 | 22 | 30 | 810 | 8.2 |
|  | L Superior Medial Gyrus | -8 | 32 | 50 | 4 | 108 | 8.13 |
|  | R Precuneus | 14 | -44 | 56 | 15 | 405 | 8.4 |

^g^. This table reports results of voxel-wise multilevel mediation searching for mediators of the dynamic trial-by-trial association between treatment expectancy (X: [Control > Placebo]) and pain on uncued medium heat trials.
