# Supplementary material for: Isolating Brain Mechanisms of Expectancy Effects on Pain: Cue-Based Stimulus Expectancies versus Placebo-Based Treatment Expectancies
Source: J Neurosci. 2025 Jul 28;45(34):e0050252025. doi: 10.1523/JNEUROSCI.0050-25.2025 (PMC12369932; doi:10.1523/JNEUROSCI.0050-25.2025)
Supplement: Figure 7-1 — Associations between placebo analgesia and differences between high pain expectancy and low pain expectancy. Download Figure 7-1, DOCX file. [file jneuro-45-e0050252025-s011.docx]

Extended Data Figure 7-1. Associations between placebo analgesia and differences between high pain expectancy and low pain expectancy.^h^

| Analysis | Contrast | Anatomical Label | x | y | z | # of voxels | Volume (mm^3^) | Max stat |
| --- | --- | --- | --- | --- | --- | --- | --- | --- |
| Whole brain FDR | Main effect pos | Nothing survives |  |  |  |  |  |  |
|  | Main effect neg | Nothing survives |  |  |  |  |  |  |
|  | Pos association with placebo | Nothing survives |  |  |  |  |  |  |
|  | Neg association with placebo | L Superior Medial Gyrus / rostral ACC | -14 | 46 | 14 | 12 | 324 | 14.09 |
| FDR within pain placebo | Main effect pos | Nothing survives |  |  |  |  |  |  |
|  | Main effect neg | Nothing survives |  |  |  |  |  |  |
|  | Pos association with placebo | Nothing survives |  |  |  |  |  |  |
|  | Neg association with placebo | Nothing survives |  |  |  |  |  |  |
| FDR within nociceptive network | Main effect pos | Nothing survives |  |  |  |  |  |  |
|  | Main effect neg | Nothing survives |  |  |  |  |  |  |
|  | Pos association with placebo | L Olfactory cortex / Area s24 | -4 | 20 | -14 | 12 | 324 | 11.89 |
|  | Neg association with placebo | Nothing survives |  |  |  |  |  |  |
| Uncorrected | Main effect pos | R Superior Orbital Gyrus / Area Fp1 (VMPFC) | 16 | 58 | -10 | 19 | 513 | 8.6 |
|  |  | R ACC / DMPFC | 4 | 38 | 28 | 21 | 567 | 8.38 |
|  |  | R Superior Frontal Gyrus (DMPFC) | 20 | 26 | 62 | 7 | 189 | 9.92 |
|  | Main effect neg | R Hippocampus / Amygdala (LB) | 26 | -4 | -20 | 25 | 675 | 9.72 |
|  |  | L Temporal Pole | -56 | 8 | -16 | 13 | 351 | 8.11 |
|  |  | L Angular Gyrus | -38 | -56 | 32 | 14 | 378 | 8.83 |
|  |  | L Superior Frontal Gyrus (DMPFC) | -16 | 14 | 56 | 6 | 162 | 9.22 |
|  | Pos association with placebo | L Inferior Temporal Gyrus / Area FG4 | -46 | -34 | -20 | 35 | 945 | 11.06 |
|  |  | R ParaHippocampal Gyrus / CA1 (Hippocampus) | 34 | -20 | -20 | 11 | 297 | 8.68 |
|  |  | L Inferior Temporal Gyrus | -56 | -56 | -20 | 23 | 621 | 8.25 |
|  |  | L Calcarine Gyrus / Area hOc2 [V2] | -4 | -94 | -10 | 21 | 567 | 11.98 |
|  |  | R Hippocampus | 22 | -22 | -14 | 9 | 243 | 8.87 |
|  |  | L Olfactory cortex / Area s24 (sgACC) | -4 | 20 | -14 | 13 | 351 | 11.89 |
|  |  | L Superior Temporal Gyrus | -64 | -44 | 14 | 16 | 432 | 7.76 |
|  |  | R SupraMarginal Gyrus / Area PFcm (IPL) | 58 | -34 | 28 | 39 | 1053 | 8.44 |
|  |  | R TPJ | 38 | -38 | 26 | 16 | 432 | 9.53 |
|  |  | R Cuneus | 14 | -70 | 32 | 24 | 648 | 8.82 |
|  |  | RPrecentral Gyrus | 22 | -26 | 62 | 18 | 486 | 9.27 |
|  | Neg association with placebo | L Cerebellum VIII | -26 | -64 | -56 | 23 | 621 | 10.51 |
|  |  | L Inferior Temporal Gyrus | -50 | 2 | -38 | 38 | 1026 | 9.17 |
|  |  | L Putamen | -26 | 2 | -4 | 12 | 324 | 7.66 |
|  |  | L Superior Medial Gyrus / rACC / pgACC | -14 | 46 | 14 | 12 | 324 | 14.09 |

^h^. This table presents results of robust regression evaluating associations between the magnitude of placebo analgesia (controlling for counterbalanced order) and differences between High Pain Expectancy and Low Pain Expectancy ([HM > LM] prior to treatment and Control > Placebo on uncued trials) on heat-evoked activation on medium trials. Figure 7A.II. depicts activation in the cluster that survives whole-brain FDR correction.
