# Supplementary material for: Isolating Brain Mechanisms of Expectancy Effects on Pain: Cue-Based Stimulus Expectancies versus Placebo-Based Treatment Expectancies
Source: J Neurosci. 2025 Jul 28;45(34):e0050252025. doi: 10.1523/JNEUROSCI.0050-25.2025 (PMC12369932; doi:10.1523/JNEUROSCI.0050-25.2025)
Supplement: Figure 7-2 — Interactions on fully-crossed trials: Uncorrected results and correction within regions of interest. Download Figure 7-2, DOCX file. [file jneuro-45-e0050252025-s012.docx]

Extended Data Figure 7-2. Interactions on fully-crossed trials: Uncorrected results and correction within regions of interest.^i^

| Analysis | Contrast | Anatomical label | x | y | z | # of voxels | Volume (mm^3^) | Max stat |
| --- | --- | --- | --- | --- | --- | --- | --- | --- |
| Correction within nociceptive regions | Positive main effect | Nothing survives |  |  |  |  |  |  |
|  | Negative main effect | Nothing survives |  |  |  |  |  |  |
|  | Positive associations | L Fusiform Gyrus / parahippocampal gyrus | -34 | -32 | -16 | 4 | 108 | 11.63 |
|  |  | R Superior Temporal Gyrus (Area TE 3) | 64 | -8 | -2 | 1 | 27 | 10.45 |
|  | Negative associations | Nothing survives |  |  |  |  |  |  |
| Uncorrected | Positive main effect | R Cerebellum Crus 2 | 22 | -80 | -34 | 5 | 135 | 7.49 |
|  |  | L Cerebellum Crus 1 | -26 | -74 | -26 | 30 | 810 | 9.21 |
|  | Negative main effect | L Temporal Pole, contiguous with VLPFC | -44 | 22 | -16 | 7 | 189 | 7.86 |
|  |  | MPFC | 2 | 68 | 14 | 29 | 783 | 7.91 |
|  | Positive associations | L Cerebellum Crus 1 | -40 | -58 | -38 | 18 | 486 | 8.94 |
|  |  | L Superior Temporal Gyrus, contiguous with Anterior Insula | -50 | 4 | -14 | 100 | 2700 | 12.88 |
|  |  | R Lingual Gyrus (Area hOc2 [V2]), contiguous with cerebellum | 10 | -76 | -2 | 467 | 12609 | 11.27 |
|  |  | R Superior Temporal Gyrus, contiguous with Anterior Insula | 46 | 2 | -14 | 47 | 1269 | 10.3 |
|  |  | L Hippocampus, contiguous with Parahippocampal Gyrus | -34 | -28 | -14 | 21 | 567 | 11.63 |
|  |  | Posterior Hippocampus | 38 | -40 | -8 | 82 | 2214 | 13.29 |
|  |  | R Hippocampus | 32 | -28 | -4 | 13 | 351 | 12.47 |
|  |  | L Calcarine Gyrus (Area hOc1 [V1]) | -10 | -88 | 2 | 70 | 1890 | 10.72 |
|  |  | L Middle Temporal Gyrus | -64 | -40 | 4 | 30 | 810 | 10.18 |
|  |  | R Superior Temporal Gyrus (Area TE 3) | 64 | -10 | -2 | 10 | 270 | 10.45 |
|  |  | L Middle Occipital Gyrus (Area hOc1 [V1]) | -10 | -106 | 2 | 12 | 324 | 8.29 |
|  |  | R Middle Occipital Gyrus (Area hOc4la) | 50 | -76 | 4 | 18 | 486 | 10.52 |
|  |  | R IFG p. Triangularis | 40 | 26 | 4 | 45 | 1215 | 10.36 |
|  |  | L Middle Temporal Gyrus (Area hOc4la) | -50 | -70 | 4 | 15 | 405 | 8.91 |
|  |  | L Middle Temporal Gyrus / SII | -46 | -50 | 16 | 51 | 1377 | 9.12 |
|  |  | L Precuneus | -22 | -50 | 8 | 20 | 540 | 11.89 |
|  |  | R Rostral ACC | 4 | 22 | 4 | 15 | 405 | 9.19 |
|  |  | Posterior Cingulate Cortex | 4 | -32 | 16 | 30 | 810 | 13.09 |
|  |  | L IFG p. Triangularis (Area 45) | -52 | 20 | 22 | 32 | 864 | 10.16 |
|  |  | R Middle Frontal Gyrus | 38 | 50 | 28 | 17 | 459 | 8.71 |
|  |  | L Precuneus | -10 | -56 | 46 | 16 | 432 | 8.62 |
|  |  | R Superior Frontal Gyrus | 22 | 20 | 52 | 47 | 1269 | 11.23 |
|  |  | L Precentral Gyrus | -28 | -22 | 58 | 18 | 486 | 8.88 |
|  |  | L Precentral Gyrus | -38 | 2 | 62 | 26 | 702 | 12.69 |
|  |  | RPrecentral Gyrus | 28 | -26 | 62 | 15 | 405 | 10.61 |
|  | Negative associations | R Cerebellum VIII | 20 | -52 | -56 | 16 | 432 | 10.78 |
|  |  | L Cerebellum VIII | -20 | -58 | -58 | 13 | 351 | 10.3 |
|  |  | R Middle Orbital Gyrus | 44 | 56 | -10 | 4 | 108 | 11.49 |
|  |  | R Insula Lobe (Area OP3 [VS]) | 38 | -4 | 10 | 27 | 729 | 11.52 |
|  |  | R Superior Medial Gyrus (Area Fp1 ) | 10 | 68 | 16 | 7 | 189 | 8.39 |
|  |  | R Middle Frontal Gyrus | 20 | 58 | 28 | 18 | 486 | 11.08 |

^i^. This table presents results of robust regression evaluating associations between the magnitude of placebo analgesia (controlling for counterbalanced order) and interactions of Treatment Expectancy ([Control-Placebo]) and Stimulus Expectancy ([High Cue-Low Cue]) on heat-evoked activation on medium trials that were crossed with both types of expectancy. See Table 6 in the main manuscript for whole brain FDR-corrected results.
