# Supplementary material for: Isolating Brain Mechanisms of Expectancy Effects on Pain: Cue-Based Stimulus Expectancies versus Placebo-Based Treatment Expectancies
Source: J Neurosci. 2025 Jul 28;45(34):e0050252025. doi: 10.1523/JNEUROSCI.0050-25.2025 (PMC12369932; doi:10.1523/JNEUROSCI.0050-25.2025)
Supplement: Figure 7-3 — Associations between placebo analgesia and differences between stimulus and treatment expectancy effects: Uncorrected results. Download Figure 7-3, DOCX file. [file jneuro-45-e0050252025-s013.docx]

Extended Data Figure 7-3. Associations between placebo analgesia and differences between stimulus and treatment expectancy effects: Uncorrected results.^j^

| Contrast | Anatomical label | x | y | z | # of voxels | Volume (mm^3^) | Max stat |
| --- | --- | --- | --- | --- | --- | --- | --- |
| Positive main effect | R Rolandic Operculum, contiguous with SII and dpInsula | 52 | -8 | 16 | 30 | 810 | 7.62 |
|  | L DMPFC, contiguous with L DMPFC | -26 | 28 | 28 | 9 | 243 | 7.88 |
| Negative main effect | R Cerebellum Crus 1 | 26 | -70 | -34 | 7 | 189 | 8.72 |
| Positive association | Lobule IX Hem | -4 | -64 | -52 | 19 | 513 | 10.82 |
|  | L Cerebellum, contiguous with brainstem | -8 | -46 | -32 | 11 | 297 | 7.84 |
|  | L Inferior Temporal Gyrus | -64 | -40 | -16 | 73 | 1971 | 13.84 |
|  | R Inferior Temporal Gyrus | 64 | -34 | -16 | 15 | 405 | 8.36 |
|  | Bilateral Mid Orbital Gyrus (Area Fp2), VMPFC | 4 | 58 | -4 | 207 | 5589 | 10.92 |
|  | R Temporal Pole | 52 | 8 | -14 | 7 | 189 | 8.6 |
|  | R Middle Temporal Gyrus | 62 | -20 | -8 | 32 | 864 | 10.94 |
|  | L IFG p. Orbitalis (latPFC/VLPFC) | -44 | 44 | -4 | 51 | 1377 | 10.24 |
|  | L Middle Temporal Gyrus | -58 | -40 | 4 | 9 | 243 | 9.67 |
|  | R IFG p. Triangularis | 46 | 38 | 10 | 14 | 378 | 10.08 |
|  | L Middle Temporal Gyrus | -46 | -56 | 20 | 43 | 1161 | 12.11 |
|  | R Superior Temporal Gyrus (Area PFm (IPL)) | 56 | -44 | 22 | 17 | 459 | 9.87 |
|  | R Middle Frontal Gyrus | 44 | 16 | 52 | 73 | 1971 | 15.62 |
|  | R Superior Frontal Gyrus | 20 | 20 | 50 | 33 | 891 | 8.26 |
|  | L Inferior Parietal Lobule (Area PFm (IPL)) | -50 | -56 | 50 | 3 | 81 | 9.52 |
|  | L Middle Frontal Gyrus | -34 | 10 | 62 | 52 | 1404 | 10.29 |
| Negative association | R Cerebellum Crus 1 | 38 | -76 | -22 | 43 | 1161 | 9.28 |
|  | L Calcarine Gyrus (Area hOc1 [V1]) | 2 | -86 | -10 | 41 | 1107 | 9.81 |
|  | R ParaHippocampal Gyrus | 34 | -40 | -8 | 27 | 729 | 8.55 |
|  | L Temporal Cortex | -44 | -44 | -8 | 14 | 378 | 9.18 |
|  | R Occipital Lobe | 34 | -58 | 20 | 15 | 405 | 10.08 |
|  | L Occipital Lobe | -26 | -50 | 32 | 26 | 702 | 9.55 |
|  | L SMA | -16 | -50 | 46 | 38 | 1026 | 9.03 |

^j^. This table presents uncorrected voxelwise results of robust regression evaluating associations between the magnitude of placebo analgesia (controlling for counterbalanced order) and differences between pure Treatment Expectancy ([Control-Placebo] on uncued medium trials) and pure Stimulus Expectancy ([High Cue-Low Cue] prior to treatment) on heat-evoked activation on medium trials. No regions survived correction within nociceptive regions or *a priori* regions involved in pain and placebo. See Table 7 in the main manuscript for whole brain FDR-corrected results.
