# Supplementary material for: Isolating Brain Mechanisms of Expectancy Effects on Pain: Cue-Based Stimulus Expectancies versus Placebo-Based Treatment Expectancies
Source: J Neurosci. 2025 Jul 28;45(34):e0050252025. doi: 10.1523/JNEUROSCI.0050-25.2025 (PMC12369932; doi:10.1523/JNEUROSCI.0050-25.2025)
Supplement: Figure 8-2 — Treatment expectancy effects on changes over time. Download Figure 8-2, DOCX file. [file jneuro-45-e0050252025-s015.docx]

Extended Data Figure 8-2. Treatment expectancy effects on changes over time.^k^

| Analysis | Contrast | Anatomical Label | x | y | z | # of voxels | Volume (mm^3^) | maxstat |
| --- | --- | --- | --- | --- | --- | --- | --- | --- |
| Whole brain correction | Main effect pos | Nothing survives |  |  |  |  |  |  |
|  | Main effect neg | Nothing survives |  |  |  |  |  |  |
|  | Positive Association | L dACC | -14 | 16 | 38 | 28 | 756 | 12.85 |
|  | Negative Association | R Middle Frontal Gyrus | 44 | 40 | 20 | 53 | 1431 | 12.32 |
|  |  | R Superior Frontal Gyrus | 16 | 52 | 38 | 77 | 2079 | 13.32 |
|  |  | R dACC | 8 | 10 | 26 | 3 | 81 | 12.36 |
| Correction within placebo regions | Main effect pos | Nothing survives |  |  |  |  |  |  |
|  | Main effect neg | Nothing survives |  |  |  |  |  |  |
|  | Positive Association | Nothing survives |  |  |  |  |  |  |
|  | Negative association | Right middle orbitofrontal gyrus / VLPFC / lateral OFC | 40 | 50 | -8 | 10 | 270 | 9.83 |
| Uncorrected | Main effect pos | R Superior Temporal Gyrus / Area TE 3 | 68 | -32 | 8 | 18 | 486 | 10.82 |
|  | Main effect neg | R Superior Orbital Gyrus / Area Fo3 / mOFC | 20 | 26 | -20 | 11 | 297 | 8.64 |
|  |  | L Precentral Gyrus | -44 | 4 | 22 | 25 | 675 | 11.39 |
|  | Positive Association | R Cerebellum | 16 | -92 | -20 | 14 | 378 | 11.37 |
|  |  | L IFG p. Orbitalis | -38 | 34 | -16 | 6 | 162 | 9.15 |
|  |  | L Calcarine Gyrus / Area hOc1 [V1] | 2 | -88 | -8 | 36 | 972 | 9.39 |
|  |  | R Putamen, contiguous with Caudate | 20 | 10 | 8 | 122 | 3294 | 10.6 |
|  |  | R Putamen | 32 | -10 | -8 | 19 | 513 | 9.55 |
|  |  | L Putamen | -22 | 8 | -8 | 33 | 891 | 8.54 |
|  |  | Thal: Parietal | 26 | -22 | 2 | 22 | 594 | 9.61 |
|  |  | R Insula Lobe | 46 | -4 | -2 | 12 | 324 | 8.44 |
|  |  | L IFG p. Opercularis | -44 | 10 | 2 | 11 | 297 | 8.6 |
|  |  | Pregenual ACC | 2 | 38 | 2 | 13 | 351 | 8.43 |
|  |  | L Middle Temporal Gyrus | -40 | -68 | 8 | 19 | 513 | 8.78 |
|  |  | L SupraMarginal Gyrus / Area PFop (IPL) | -56 | -28 | 20 | 6 | 162 | 7.5 |
|  |  | R Superior Occipital Lobe | 26 | -62 | 34 | 8 | 216 | 7.77 |
|  |  | L dACC | -14 | 16 | 38 | 28 | 756 | 12.85 |
|  |  | L Posterior-Medial Frontal | -4 | 8 | 46 | 20 | 540 | 8.23 |
|  | Negative Association | R Cerebellum Crus 2 | 40 | -74 | -44 | 35 | 945 | 10.15 |
|  |  | L Cerebellum Crus 1 | -22 | -88 | -22 | 6 | 162 | 10.33 |
|  |  | L Inferior Temporal Gyrus | -62 | -32 | -22 | 19 | 513 | 9.39 |
|  |  | R Middle Temporal Gyrus | 62 | -28 | -8 | 110 | 2970 | 11.89 |
|  |  | L Middle Temporal Gyrus | -64 | -32 | -8 | 29 | 783 | 10.59 |
|  |  | L IFG p. Orbitalis, contiguous with VLPFC, latPFC, anterior Insula | -40 | 38 | -4 | 139 | 3753 | 9.88 |
|  |  | R Middle Orbital Gyrus / VLPFC / latPFC | 40 | 46 | -8 | 78 | 2106 | 9.83 |
|  |  | R Superior Orbital Gyrus / Area Fo3 / VLPFC / OFC | 20 | 46 | -14 | 6 | 162 | 8.97 |
|  |  | L Inferior Occipital Gyrus / Area hOc3v [V3v] | -20 | -94 | -8 | 4 | 108 | 9.38 |
|  |  | L Middle Temporal Gyrus | -68 | -40 | 2 | 8 | 216 | 9.65 |
|  |  | L Middle Frontal Gyrus | -32 | 44 | 2 | 13 | 351 | 7.72 |
|  |  | R Middle Frontal Gyrus / Area Fp1 | 26 | 56 | 4 | 13 | 351 | 9.99 |
|  |  | R Middle Frontal Gyrus | 44 | 40 | 20 | 53 | 1431 | 12.32 |
|  |  | L IFG p. Triangularis / Area 45 | -50 | 32 | 20 | 93 | 2511 | 9.37 |
|  |  | L Middle Frontal Gyrus | -38 | 58 | 14 | 19 | 513 | 8.66 |
|  |  | R Superior Temporal Gyrus / Area PGa (IPL) | 64 | -50 | 22 | 9 | 243 | 8.6 |
|  |  | R Superior Frontal Gyrus | 16 | 52 | 38 | 77 | 2079 | 13.32 |
|  |  | R Middle Occipital Gyrus / Area PGp (IPL) | 40 | -68 | 32 | 19 | 513 | 8.29 |
|  |  | L Middle Frontal Gyrus | -50 | 20 | 44 | 18 | 486 | 10.19 |
|  |  | R Middle Frontal Gyrus | 38 | 28 | 50 | 14 | 378 | 10.01 |
|  |  | R S1 | 22 | -34 | 52 | 14 | 378 | 8.93 |
|  |  | R Postcentral Gyrus / Area 1 | 52 | -20 | 56 | 18 | 486 | 9.25 |
|  |  | L Middle Frontal Gyrus | -44 | 14 | 52 | 12 | 324 | 9.38 |
|  |  | R Middle Frontal Gyrus | 34 | 10 | 56 | 23 | 621 | 7.26 |

^k^. This table presents results of robust regression evaluating differences across time as a function of treatment expectancy ([Control x time - Placebo x time]) and associations with the magnitude of placebo analgesia (controlling for counterbalanced order).
