# Supplementary material for: Isolating Brain Mechanisms of Expectancy Effects on Pain: Cue-Based Stimulus Expectancies versus Placebo-Based Treatment Expectancies
Source: J Neurosci. 2025 Jul 28;45(34):e0050252025. doi: 10.1523/JNEUROSCI.0050-25.2025 (PMC12369932; doi:10.1523/JNEUROSCI.0050-25.2025)
Supplement: Figure 8-3 — Impact of cue presentation on brain response. Download Figure 8-3, DOCX file. [file jneuro-45-e0050252025-s016.docx]

Extended Data Figure 8-3. Impact of cue presentation on brain response.^l^

| Analysis | Contrast | Anatomical Label | x | y | z | # of voxels | Volume (mm^3^) | Max stat |
| --- | --- | --- | --- | --- | --- | --- | --- | --- |
| Whole brain FDR | Cues > No cues | R Cerebellum VIII | 32 | -56 | -46 | 5 | 135 | 7.18 |
|  |  | R Temporal Pole | 32 | 20 | -32 | 6 | 162 | 7.75 |
|  |  | R ParaHippocampal Gyrus / CA1 (Hippocampus)) / Amygdala | 20 | -14 | -22 | 6 | 162 | 7.56 |
|  |  | L Temporal Pole | -34 | 10 | -22 | 14 | 378 | 7.05 |
|  |  | R ParaHippocampal Gyrus / amygdala | 20 | 8 | -20 | 16 | 432 | 7.32 |
|  |  | R Rectal Gyrus (Area Fo2) | 10 | 22 | -22 | 4 | 108 | 9.09 |
|  |  | R Inferior Temporal Gyrus | 50 | -56 | -14 | 64 | 1728 | 10.06 |
|  |  | R Superior Temporal Gyrus | 56 | -26 | 10 | 1106 | 29862 | 20.72 |
|  |  | L Lingual Gyrus (Area hOc4v [V4(v)]) | -26 | -86 | -14 | 3 | 81 | 7.05 |
|  |  | R Extended Amygdala | 16 | -4 | -10 | 4 | 108 | 7.39 |
|  |  | R Superior Orbital Gyrus (Area Fp1) | 32 | 62 | -8 | 5 | 135 | 7.28 |
|  |  | L Caudate Nucleus | -10 | 14 | 2 | 14 | 378 | 10.3 |
|  |  | R Caudate Nucleus | 14 | 14 | 10 | 24 | 648 | 8.25 |
|  |  | Area hOc4lp | 44 | -88 | 4 | 3 | 81 | 8.98 |
|  |  | Rostral ACC | -20 | 34 | 14 | 10 | 270 | 8.14 |
|  |  | L Caudate Nucleus | -14 | 16 | 14 | 12 | 324 | 8.92 |
|  |  | R DMPFC | 28 | 44 | 16 | 8 | 216 | 7.51 |
|  |  | R Middle Occipital Gyrus | 32 | -74 | 32 | 35 | 945 | 11.63 |
|  |  | L rdACC | -10 | 20 | 26 | 4 | 108 | 8.14 |
|  |  | L Superior Frontal Gyrus | -22 | 34 | 32 | 7 | 189 | 7.87 |
|  |  | R MCC / MPFC / DMPFC | 8 | 38 | 32 | 9 | 243 | 8.66 |
|  |  | R Middle Frontal Gyrus | 28 | 50 | 34 | 17 | 459 | 8.06 |
|  |  | L Superior Frontal Gyrus | -28 | 44 | 38 | 21 | 567 | 9.77 |
|  |  | R Superior Frontal Gyrus | 20 | 46 | 40 | 27 | 729 | 7.1 |
|  |  | R Middle Frontal Gyrus | 52 | 26 | 38 | 7 | 189 | 10.53 |
|  |  | R Middle Frontal Gyrus | 22 | 16 | 46 | 14 | 378 | 7.41 |
|  |  | R Postcentral Gyrus (Area 3b) | 26 | -38 | 50 | 7 | 189 | 8.05 |
|  |  | R Posterior-Medial Frontal (Area 4a) | 2 | -20 | 58 | 29 | 783 | 12.2 |
|  |  | R Middle Frontal Gyrus | 32 | 32 | 50 | 21 | 567 | 8.5 |
|  |  | R Superior Frontal Gyrus | 26 | 26 | 58 | 3 | 81 | 7.96 |
|  | No Cues > Cues | R Superior Temporal Gyrus | 56 | -26 | 10 | 1106 | 29862 | 20.72 |
|  |  | L Superior Temporal Gyrus | -52 | -28 | 10 | 741 | 20007 | 24.65 |
|  |  | L Thalamus | -16 | -32 | 8 | 8 | 216 | 8.22 |
|  |  | Posterior Cingulate Cortex | -2 | -34 | 22 | 57 | 1539 | 11.78 |
|  |  | L Superior Occipital Gyrus | -16 | -76 | 28 | 22 | 594 | 8.44 |
|  |  | R MCC | 10 | -46 | 38 | 60 | 1620 | 14.55 |
|  |  | L Precuneus | -10 | -52 | 44 | 45 | 1215 | 9.74 |
| Correction within bHvL PPN | Cues > No cues | R Inferior Temporal Gyrus (Area FG4) | 46 | -52 | -14 | 22 | 594 | 8.39 |
|  | No cues > Cues | R Temporal Pole (Area TE 3) | 56 | 10 | -14 | 2 | 54 | 8.91 |
|  |  | R Superior Temporal Gyrus (Area TE 3) | 68 | -22 | 8 | 14 | 378 | 14.74 |
|  |  | R Superior Temporal Gyrus (Area TE 1.0) | 56 | -8 | 2 | 3 | 81 | 9.65 |
|  |  | L Superior Temporal Gyrus (Area PFcm (IPL)) | -46 | -38 | 14 | 20 | 540 | 12.85 |
|  |  | Posterior Cingulate Cortex | -4 | -32 | 28 | 18 | 486 | 9.71 |
| Uncorrected | Cues > No cues | R Inferior Temporal Gyrus | 50 | -56 | -14 | 64 | 1728 | 10.06 |
|  |  | R Superior Temporal Gyrus, contiguous with middle insula, dorsal posterior insula | 56 | -26 | 10 | 1106 | 29862 | 20.72 |
|  |  | L Caudate Nucleus | -10 | 14 | 2 | 14 | 378 | 10.3 |
|  |  | R Caudate Nucleus | 14 | 14 | 10 | 24 | 648 | 8.25 |
|  |  | R Middle Occipital Gyrus | 32 | -74 | 32 | 35 | 945 | 11.63 |
|  |  | L Superior Frontal Gyrus | -28 | 44 | 38 | 21 | 567 | 9.77 |
|  |  | R Superior Frontal Gyrus | 20 | 46 | 40 | 27 | 729 | 7.1 |
|  |  | R Posterior-Medial Frontal / Area 4a | 2 | -20 | 58 | 29 | 783 | 12.2 |
|  |  | R Middle Frontal Gyrus | 32 | 32 | 50 | 21 | 567 | 8.5 |
|  | No cues > Cues | R Superior Temporal Gyrus, contiguous with middle insula, dorsal posterior insula | 56 | -26 | 10 | 1106 | 29862 | 20.72 |
|  |  | L Superior Temporal Gyrus | -52 | -28 | 10 | 741 | 20007 | 24.65 |
|  |  | Posterior Cingulate cortex | -2 | -34 | 22 | 57 | 1539 | 11.78 |
|  |  | R MCC | 10 | -46 | 38 | 60 | 1620 | 14.55 |
|  |  | L Precuneus | -10 | -52 | 44 | 45 | 1215 | 9.74 |

^l^. This table presents results of robust regression evaluating medium heat as a function of whether or not stimulation was preceded by a stimulus expectancy cue.
